# Effect of Water on a Hydrophobic Deep Eutectic Solvent

**DOI:** 10.1021/acs.jpcb.1c08170

**Published:** 2022-01-09

**Authors:** Henri Kivelä, Mikko Salomäki, Petteri Vainikka, Ermei Mäkilä, Fabrizio Poletti, Stefano Ruggeri, Fabio Terzi, Jukka Lukkari

**Affiliations:** † Department of Chemistry, University of Turku, FI-20014 Turku, Finland; ‡ Department of Physics and Astronomy, University of Turku, FI-20014 Turku, Finland; § Turku University Centre for Surfaces and Materials (MatSurf), FI-20014 Turku, Finland; ∥ Doctoral School for Chemical and Physical Sciences, University of Turku, FI-20014 Turku, Finland; ⊥ Electrochemical Sensors Group, Department of Chemical and Geological Sciences, University of Modena and Reggio Emilia, Via Giuseppe Campi, 103, I-41125 Modena, Italy

## Abstract

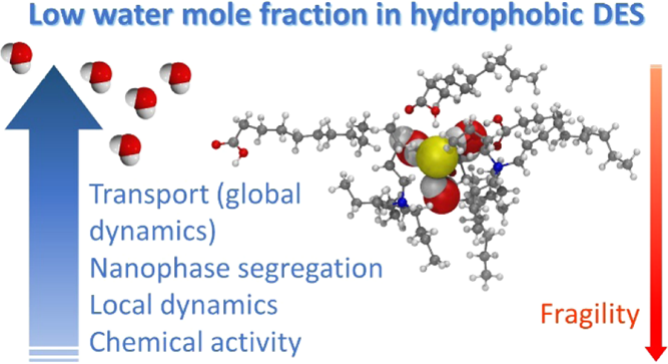

Deep eutectic solvents (DESs) formed by hydrogen bond donors and acceptors are a promising new class of solvents. Both hydrophilic and hydrophobic binary DESs readily absorb water, making them ternary mixtures, and a small water content is always inevitable under ambient conditions. We present a thorough study of a typical hydrophobic DES formed by a 1:2 mole ratio of tetrabutyl ammonium chloride and decanoic acid, focusing on the effects of a low water content caused by absorbed water vapor, using multinuclear NMR techniques, molecular modeling, and several other physicochemical techniques. Already very low water contents cause dynamic nanoscale phase segregation, reduce solvent viscosity and fragility, increase self-diffusion coefficients and conductivity, and enhance local dynamics. Water interferes with the hydrogen-bonding network between the chloride ions and carboxylic acid groups by solvating them, which enhances carboxylic acid self-correlation and ion pair formation between tetrabutyl ammonium and chloride. Simulations show that the component molar ratio can be varied, with an effect on the internal structure. The water-induced changes in the physical properties are beneficial for most prospective applications but water creates an acidic aqueous nanophase with a high halide ion concentration, which may have chemically adverse effects.

## Introduction

Deep eutectic solvents (DESs) are eutectic mixtures based on Brønsted or Lewis acids that are liquid at or near the room temperature,^1−3^ although a precise definition of a DES requires a more subtle analysis.^3,4^ DESs differ from the well-known ionic liquids (ILs), which are liquid electrolytes formed of cationic and anionic species. DESs are divided into four to five classes, of which the type III solvents, consisting of hydrogen bond donors (HBDs) and hydrogen bond acceptors (HBAs), are the most studied. The hydrogen bond donors and acceptors are small organic acids, amines, alcohols, amides, and tetra alkyl ammonium halides, generally present in the ratio of 1:1 or 2:1.^1,2,5,6^ Hydrogen bonding between the HBD and the anion of the salt stabilizes the solvent structure and causes a considerable depression of the freezing point. These solvents are considered excellent choices for sustainable development and green chemistry because they are often nontoxic, biocompatible and biodegradable, low-cost, and easily prepared in a pure state from readily available components.^1−6^ They are prepared either by heating the mixture until it liquefies and cooling it back to room temperature, by adding water to the mixture and freeze-drying it, or by grinding the mixture to form a paste that liquefies with time.^7,8^ They differ from most ionic liquids, which may require complex chemical synthesis and expensive reagents, and are often toxic and not biodegradable. Type III DESs have a wide range of applications as organic reaction media, in metal and natural product extraction, catalysis, nanotechnology and material preparation, gas and metal dissolution, metal electrodeposition, and other electrochemical applications.^1−3,6,9−14^ Among them, natural deep eutectic solvents (NADESs) are highly biocompatible because they consist of natural compounds or primary metabolites and often contain also water.^10,15,16^ In fact, the survival of animals in extreme conditions has been attributed to the formation of NADESs in their cells.^17^

The physicochemical properties of DESs vary substantially depending on the components.^1,2,13^ They generally have very low volatility, relatively high viscosity, and low ionic conductivity.^2,6,13^ Their surface tension is usually higher than that of molecular organic solvents and their polarity is similar to common HBD-type solvents (alcohols, amines).^2,13,18^ The properties of DESs are often discussed using the hole theory of liquids to rationalize, especially, the transport properties in these solvents,^19−22^ although other theoretical frameworks, including the classical regular solution theory, have also been applied.^23−29^ NMR and other spectroscopic techniques yield information about the average molecular-level structure and interactions,^2^ and pulsed field gradient NMR has been used to measure the self-diffusion coefficients of the DES components.^23,30,31^ Detailed molecular-level information can be obtained by X-ray or neutron scattering, quantum mechanical calculations, and molecular dynamics simulations.^8,32−45^

The type III DESs can be hydrophilic or hydrophobic. Hydrophilic DESs are formed from small quaternary ammonium salts, e.g., choline chloride, and small HBDs, typically urea, ethylene glycol, glycerol, or small organic acids, and are well characterized in the literature. They have relatively low viscosity, reasonable conductivity, and a wide potential window, which makes them suitable as solvents in electrochemical applications. They are also highly hygroscopic, and the water content needs to be quantified as it significantly affects their physical properties.^46−51^ On the other hand, the so-called hydrophobic DESs have only recently been reported.^3,52,53^ They typically consist of long-chain fatty acids and quaternary ammonium salts with long alkyl chains or small organic molecules (menthol, thymol, ibuprofen, organic acids) and have been shown to be promising solvents for the extraction of natural compounds and metals from aqueous solutions.^53−58^ They have higher viscosity and lower conductivity than hydrophilic DESs but we have recently demonstrated their use as solvents in electrochemistry.^56^ Their properties are less studied but their formation, vapor pressure, density, and viscosity have been characterized and discussed using theoretical fluid models and molecular dynamics simulations.^25,26,33,59−65^

**Scheme 1:**
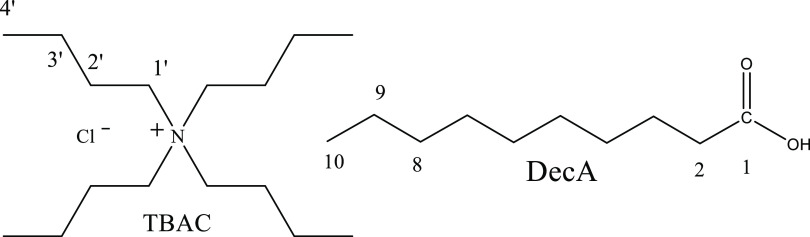
Structures of the DES Components and the Numbering of the Atoms

Depending on the water concentration, the DES–water mixtures can be divided into two categories, called water-in-DES and DES-in-water or association and hydration regimes.^36,37,51,66^ Most work on the effect of water in DESs has focused on solvents with a high water mole fraction. With typical hydrophilic DESs, the addition of 1 mole equivalent of water brings about changes in the structure but the transition between the regimes takes place only at a very high water content (>40–80 mol %).^44,45,67,68^ Hydrophilic DESs usually mix with water in any ratio, but hydrophobic DESs tend to phase-separate upon contact with an aqueous phase, which greatly limits their achievable water content.^56,61,62,69−72^ However, they can absorb water vapor from ambient, which inevitably leads to small water fractions in the solvent when working in ambient atmosphere.^56,73^ In most applications, it would be desirable to be able to work under ambient conditions, and therefore, the effects of very small amounts of water are especially interesting. In this work, we focus on a simple prototypical hydrophobic DES consisting of a 1:2 molar ratio of tetrabutyl ammonium chloride (TBAC) and decanoic acid (DecA) (Scheme 1) as a model for hydrophobic DESs. This is one of the first reported hydrophobic DESs, and it has been studied for the extraction of organic material from aqueous solutions and for electrochemical and fluorescence applications.^52,56,74^ We present, to the best of our knowledge, the first comprehensive detailed study of this DES, focusing especially on the effect of a very small water content and its implications for applications, using a wide spectrum of techniques, including molecular dynamics simulations, spectroscopic techniques, especially multinuclear NMR (pulsed field gradient, relaxation times), differential scanning calorimetry (DSC), and by measurements of density, viscosity, and electrical conductivity.

## Experimental Section

### Materials and Preparation

Tetrabutyl ammonium chloride (TBAC, Tokyo Chemical Industry, purity ≥98%) and decanoic acid (DecA, Sigma-Aldrich, purity ≥98%) were used as received but TBAC was stored under a dry argon atmosphere in a glovebox (mBraun UNIlab Pro). Ultrapure water was obtained by distilling reverse osmosis water twice in quartz vessels (Distilon 2DQ system, Bhanu Scientific Instruments). The DES was prepared from a 1:2 mole ratio of TBAC and DecA by heating the mixture at 65 °C for 3 h under stirring and allowing it to cool down to room temperature (Figure S1). Dry samples were prepared in a glovebox. DESs saturated with water (“wet” DES samples) were heated in a water bath in an ambient atmosphere and then equilibrated with water vapor at different temperatures for ca. 12 h. For details, see the Supporting Information (SI1).^56^

### Characterization

The water content was measured by Karl Fischer titration (Mettler-Toledo DL32 KF Coulometer, periodically calibrated with Hydranal-Eichstandard 5.0, Riedel de Haën). For the details on the measurement of density, viscosity, conductivity, surface tension, thermal properties, laser scattering, IR, and Raman spectra, see SI. The NMR measurements were carried out with a Bruker Avance-III 500 MHz NMR spectrometer and processed using TOPSPIN 3.5 software (Bruker). A stimulated echo ^1^H NMR method was used for the self-diffusion coefficients (see SI2–5).

### Molecular Modeling

The TBAC–DecA mixtures (1:2 mole ratio) with five different water contents were modeled at different temperatures. Some simulations were performed for 1:1 and 1:3 mole ratios to study the effect of the HBA:HBD ratio on clustering within the DES. Two force fields were utilized in this study, OPLS-AA and GROMOS54a7. The atom–atom radial distribution functions (RDFs), potentials of mean force (PMF), the Z-density profiles, and self-diffusion coefficients were calculated from the results, and a cluster analysis of the chloride–carboxylic acid hydrogen-bonding network was carried out (see SI6 and SI Appendix).

## Results and Discussion

### Water Saturation

Attempts to equilibrate the DES phase in direct contact with an aqueous solution led to the extraction of a large part of TBAC into the aqueous phase as an ion pair, often resulting in phase separation.^56,62^ Only a small fraction of DecA is extracted but it causes a drop of pH in the aqueous phase. Gentle heating will recover the liquid DES phase, but increased scattering at long wavelengths implies a nonhomogeneous phase due to micelles formed, and phase separation takes place again upon cooling. Therefore, the water content in DES was adjusted by equilibrating the DES phase with water vapor at different temperatures (from +25 to +60 °C), which also models the natural process of water contamination under ambient conditions. The process is energetically favorable because of the formation of hydrogen bonds but entropically unfavorable (Figure S10). This behavior is typical for the dissolution of gasses in liquids and suggests that the process is self-limiting. The highest attainable water levels were ca. 2.6% (w/w), corresponding to a maximum water mole fraction of ca. 0.24 (see SI1), which is little less than the previously reported saturation water content for this DES.^52^ Laser scattering studies indicated that the solution was homogeneous after equilibration with water vapor.

### Thermal Behavior

**Figure 1:**
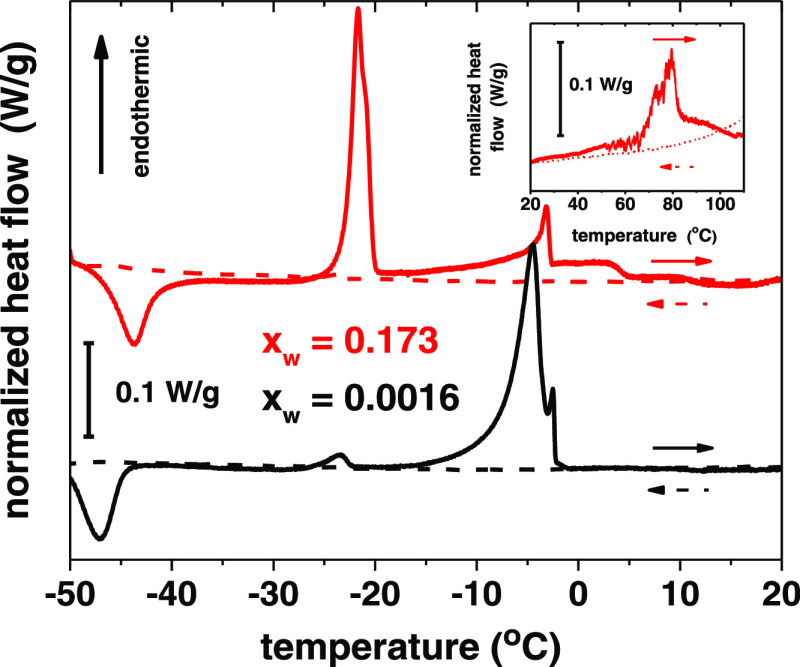
DSC curves of a dry and wet DES (water mole fractions shown) during heating (1 °C/min after 10 min at −50 °C, solid lines) and cooling (5 °C/min after 10 min at +120 °C, dashed lines). Curves are shifted for clarity. The inset shows the endothermic process with the wet sample at a high temperature.

DESs with two different water contents were subjected to thermal analysis using DSC (Figures 1 and S11). In both cases, an exothermic process takes place below −40 °C upon warming and can be attributed to cold crystallization.^50^ Two cold crystallization phenomena have been previously reported for the same DES but the lower one is below the achievable temperature range of our instrument (ca. −60 °C).^52^ Cold crystallization is a diffusion-dependent process, which implies the formation of a metastable vitrified phase upon cooling.^75^ Upon further heating, two endothermic processes are observed, with both samples at approximately the same temperatures. With the dry DES, the first endothermic peak is much smaller than the second, but the situation is reversed with the wet solvent, and we assign these peaks to two different melting processes in the DESs. The melting point of reline, a hydrophilic deep eutectic solvent, has been shown to decrease linearly with the water content,^50^ and the major endothermic peak of the TBAC–DecA DES also shifts downwards by ca. 10 °C per increase of 0.1 in the water mole fraction (Figure S12). Water in the DES turns the binary system into a ternary mixture that deviates from the ternary eutectic composition. Upon heating a solid sample, the temperature of the ternary eutectic point is reached first. With the wet DES, a relatively large part of the solid melts to liquid with the ternary eutectic composition. The system then follows the cotectic boundary line between the two two-phase regions, and a further increase in temperature leads to a gradual enrichment of the liquid phase with the majority component in the remaining binary solid until the melting of the binary eutectic is observed. The dry sample contains only a very small amount of liquid ternary eutectic composition, and the second endothermic peak is larger. In the wet sample, there is a level shift at ca. +4 °C, reported also previously,^52^ and a much smaller one at ca. +12 °C. These features are hardly observable with the dry DES, which approximates a true binary eutectic mixture in behavior, and might be attributed to a structural rearrangement of the solvent due to, e.g., changes in the hydration of the components. An endothermic process is observed only with the wet DES at temperatures above 50 °C due to the corrosion of the aluminum substrate by the acidic aqueous concentrated chloride solution in the sample,^76^ and the increased reactivity of the solvent has to be considered in possible applications. On the cooling cycle, no processes were observed in either case, which indicates supercooling to a metastable state.

### Molecular-Level Structure of the DES

Molecular dynamics simulations can yield detailed information about the structure and behavior of the DESs at the molecular level. However, the TBAC–DecA-based DES differs from most others because the TBA^+^ cation cannot form hydrogen bonds. This limits the number of possible interactions, and to the best of our knowledge, very few studies have dealt with similar DESs as in this work, however showing that hydrogen-bonding interactions between halide or the hydroxyl group and the carbonyl group were important.^61,77^ In general, the calculation of density provides a straightforward method to test the choice of the intermolecular potentials in the force field. In our case, the calculated and observed densities of the DES were well in accordance (maximum error ca. 2%; Figure S13), and the calculated density decreased linearly with temperature. The small effect (below 1%) of the water content on the observed density could not be reproduced in simulations. It would have required considerably larger computational systems and resources, which were not considered necessary in this work.

Atom–atom radial distribution functions (*g*_AB_(*r*), RDFs) are powerful tools to investigate the structure at the molecular level (see SI6 for all calculated RDFs and their detailed analysis). They also allow the calculation of the average coordination number of atom B around atom A by the cumulative integral. In the case of DES, water self-correlation allows a comparison with the known self-correlation function in pure water and the state of water in these two environments. The calculated water oxygen–water oxygen (OW–OW) and water oxygen–water hydrogen (OW–HW) radial distribution functions (Figures S14–S17) display a maximum at ca. 2.7 Å, which is assigned to the hydrogen-bonded water molecule and some smaller structures beyond it. On the other hand, the OW–HW function displays two maxima, the first at 1.75 Å and the second, broader, at ca. 3 Å, assigned to the nearest and the other hydrogen in the neighboring water molecule, respectively, in accordance to experimental data.^78^ In pure water, the average coordination number is 4.5 but it was much smaller, around 1, in our simulations. A similar low water self-coordination number has been reported for hydrated malicine (1:1 of choline chloride: malic acid), in which case, nanoscale water clusters and transient wormlike aggregates were suggested.^79^

**Figure 2:**
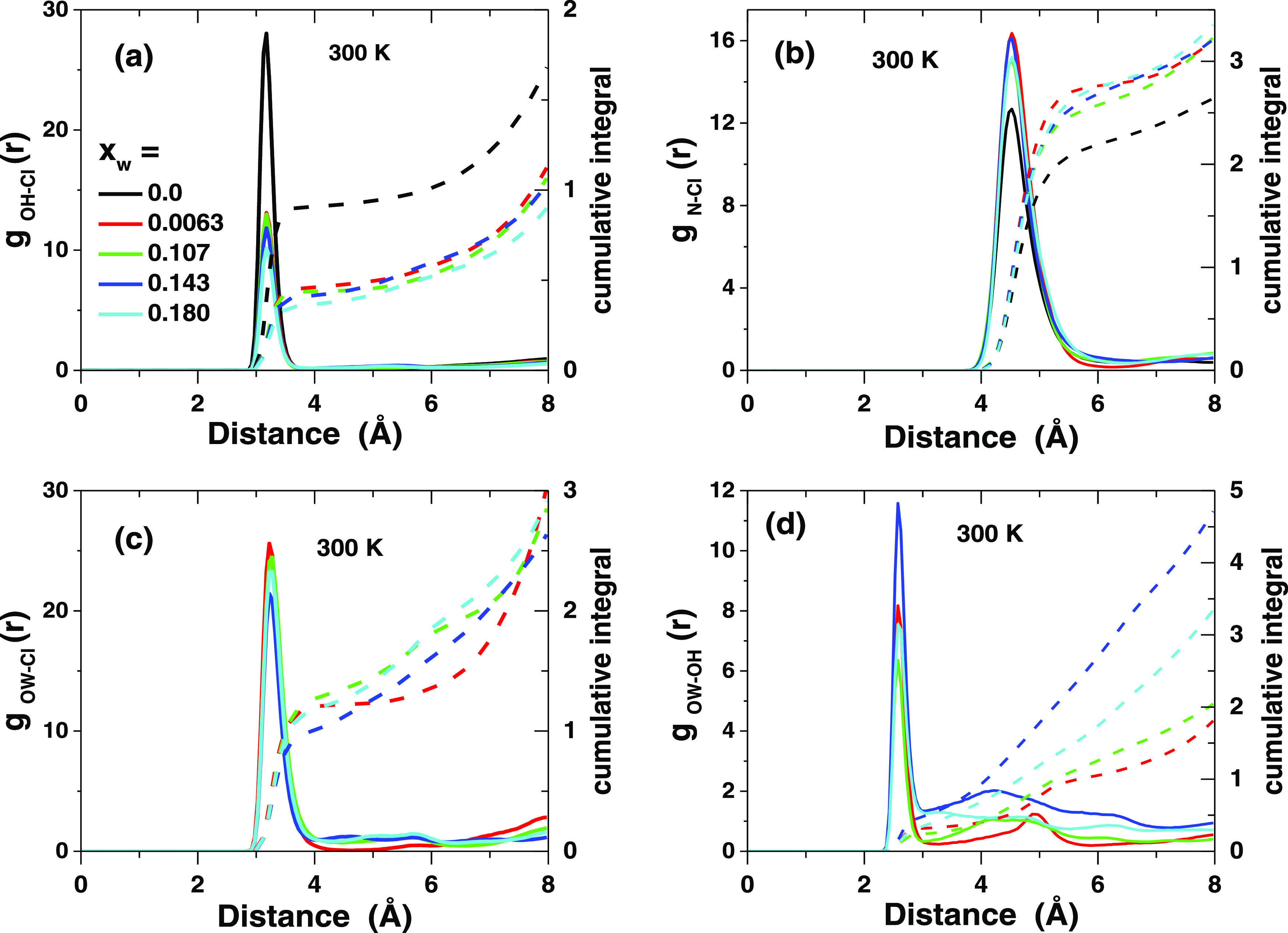
Simulated atom–atom radial distribution functions (a) *g*_OH–Cl_, (b) *g*_N–Cl_, (c) *g*_OW–Cl_, and (d) *g*_OW–OH_ at 300 K (OPLS-AA force field) in DESs with different water fractions (solid lines). The right *y*-axis refers to the value of the cumulative integral (dashed lines).

Water forms a well-defined solvation shell around the chloride anions at 3.1–3.2 Å (Cl-OW, depending on the force field used) and 2.1 Å (Cl-HW) (Figures S18–S21). These values are similar to those reported for Cl^–^ in aqueous solutions (3.1–3.20 and 2.2 Å, respectively),^80,81^ implying effective hydrogen bonding between added water and chloride. On the other hand, hydrogen bonding between the chloride anions and carboxyl hydrogens (OH) is considered the major structural factor for the DES. The *g*_OH–Cl_(*r*) functions display a sharp peak with a maximum at 3.2 Å, independent of temperature or water content, which is well in accordance with the OH···Cl hydrogen bonding (Figures 2a, S22, and S23).^82,83^ In dry DES, there is approximately one chloride around each carboxyl OH, which supports an extensive Cl···H–O hydrogen bonding. When water is present, the chloride coordination around OH drops to ca. 0.5, showing that water profoundly modifies the structure of the DES already at a very low water concentration. On the other hand, the *g*_N–Cl_(*r*) function has a broad peak at 4.5 Å, indicating a variety of possible geometries, and the peak position is independent of temperature and water content (Figures 2b, S24, and S25). The coordination number of chloride around the nitrogen in TBA^+^ is ca. 2.3 in dry DES but increases to ca. 2.7 when water is present. The whole chloride coordination sphere is closer than 6 Å to nitrogen, which shows that chloride penetrates inside the hydrocarbon arms of TBA^+^ (Figure S26) and forms a contact ion pair, similarly as observed in concentrated aqueous solutions of TBABr.^84^ In the latter case, the N–Cl distance peaks at ca. 5 Å but the coordination number is only ca. 1/3. Therefore, the simulations suggest that chloride released from the Cl···H–O hydrogen bonds binds stronger with the positively charged nitrogen, which is supported by the extraction of TBACl into an aqueous phase as an ion pair.^56^ This behavior is contrary to what has been suggested for some choline chloride-based DESs.^67,68,79^ Water also partially penetrates inside the hydrocarbon arms of the TBA^+^ cation but the hydration shell is poorly defined (Figure S27).

Water competes also with the DecA hydroxyl groups for chloride, and a sharp peak at 3.2 Å is observed in the *g*_OW–Cl_(*r*) function (Figure 2c), close to the observed Cl–O distance in concentrated aqueous HCl or solutions of divalent chlorides.^81,85^ The coordination number of Cl around water oxygen is 1.25 ± 0.25, slightly depending on temperature and water content, which suggests that all water molecules have at least one chloride ion in their coordination shell. At the same time, water becomes associated also with the carboxylic acid moieties, and the *g*_OW–OH_(*r*) function shows a sharp peak at 2.6 Å, with some less well-defined structure beyond 3.1 Å, and the average number of OH around a water molecule increases from ca. 0.15 to 0.5 with increasing water content (Figure 2d). All of these changes also modify the carboxylic acid OH–OH self-correlation (Figures S28–S30). In dry DES, the *g*_OH–OH_(*r*) self-correlation function displays a complicated pattern with a maximum at 4.7 ± 0.1 Å. When water is present, this peak grows and the shoulder at 6 Å is suppressed, and the self-coordination of the carboxylic acid groups increases (see also the cluster analysis later).

**Figure 3:**
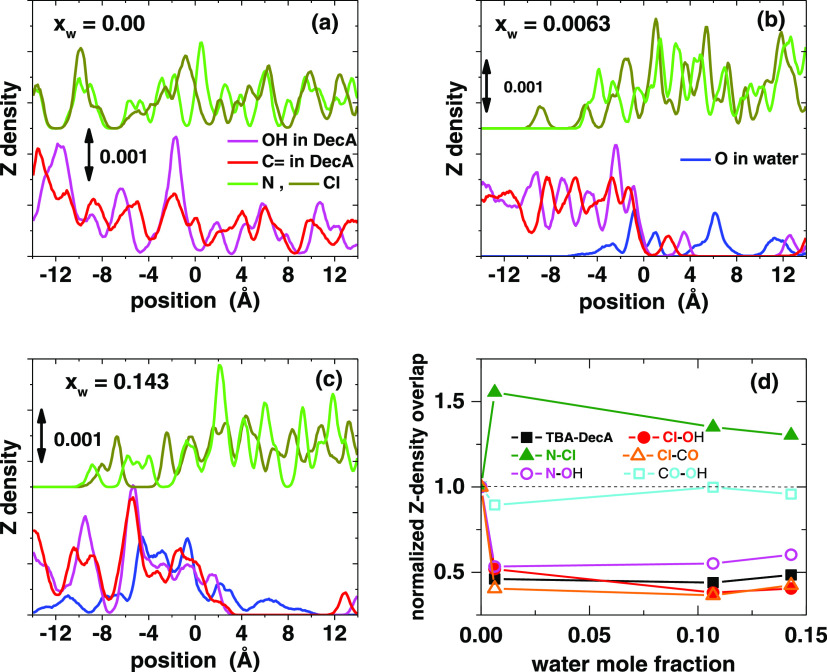
(a–c) Z-density profiles of the components of DES (TBAC/DecA 1:2) at different water fractions. Curves are vertically shifted for clarity. (d) Integrated overlap Z densities of atoms (colored, in bold) normalized by the overlap in the dry DES.

**Figure 4:**
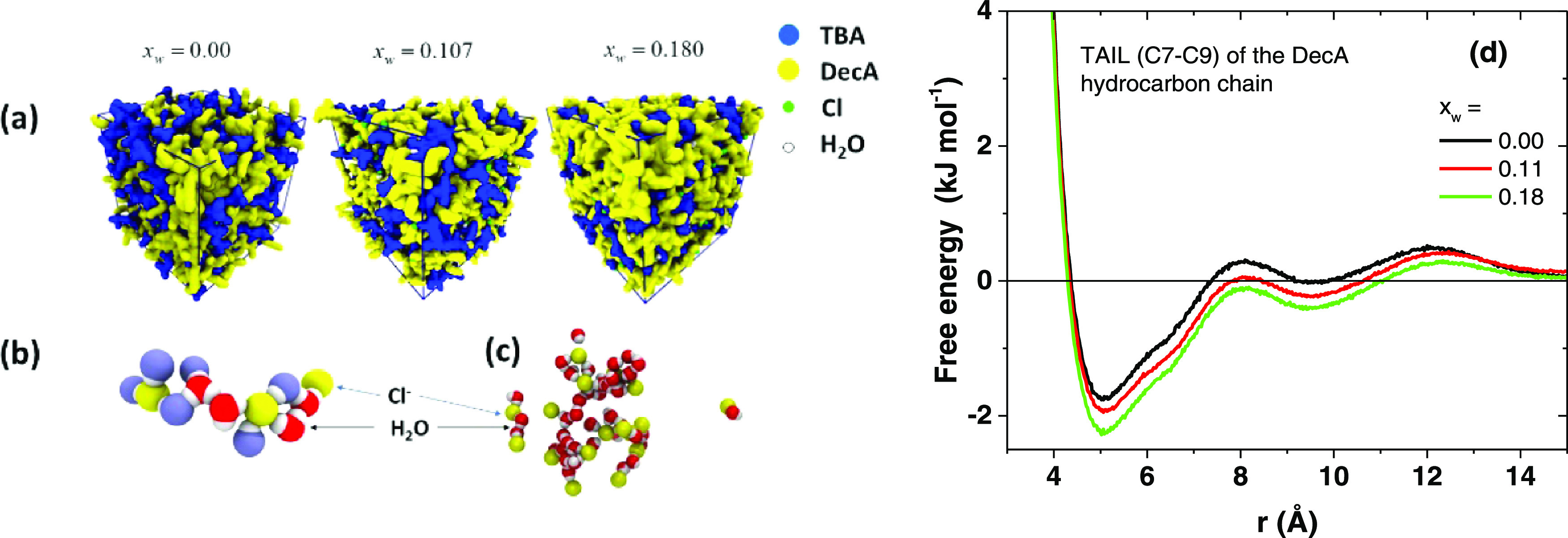
(a) Snapshots of the evolution of the dynamic segregation as a function of the water mole fraction (at 280 K). Yellow areas contain DecA, blue areas contain TBA, and green and white areas contain Cl and water, respectively; (b) dynamic aggregation of water, chloride (yellow) and oxygen from the OH group of DecA (light blue); and (c) a snapshot of the dynamic water and chloride network. The water mole fraction is 0.180 in panels (b) and (c), and other molecules are removed. In panel (d), the calculated potentials of mean force for the association of the mass centers of the tail part (C7–C9) of DecA at different water mole fractions.

In summary, all of these results from the RDF simulations show that already very small amounts of water significantly disturb the hydrogen bonding between the chloride ions and the carboxylic acid groups of DecA because of the strong hydration of the chloride anions and the carboxylic OH, in accordance with water acting as a second HBD in the DES.^79^ This leads also to the enhancement of the TBA^+^Cl^–^ ion pair formation and the clustering of the DecA molecules. An illustrative qualitative view of the effect of water on the structure of DES is provided by the Z-density calculations (Figures 3 and S38). Because the chloride ions avoid the low-polarity hydrocarbon regions, the hydrocarbon chains of DecA tend to be associated with each other even in the dry solvent (Figures 4a and S33), as also recently suggested for another hydrophobic DES.^64,65^ However, in dry DES, the components (TBAC and DecA) are well mixed but already a low water content brings about enhanced separation into larger dynamic TBAC-rich and DecA-rich nanophases (Figure 4). We attribute the nanophase formation to the interplay between the van der Waals interactions of the chains and the weakening Cl···HOOC hydrogen bonding, which makes the association of the hydrocarbon chains thermodynamically more favorable (Figure 4d; all parts of the chain behaved similarly, Figure S34). Water and chloride become associated and tend to form larger connected ribbonlike nanostructures (Figure 4b,c; note that the structures support hydrogen bonding, not dipole–dipole interactions), “wormlike” clusters.^36^ Such dynamic heterogeneity at the molecular level has previously been used to explain experimental observations in neat DESs and at high water content.^31,34,43,86−88^

### Density and Viscosity

**Figure 5:**
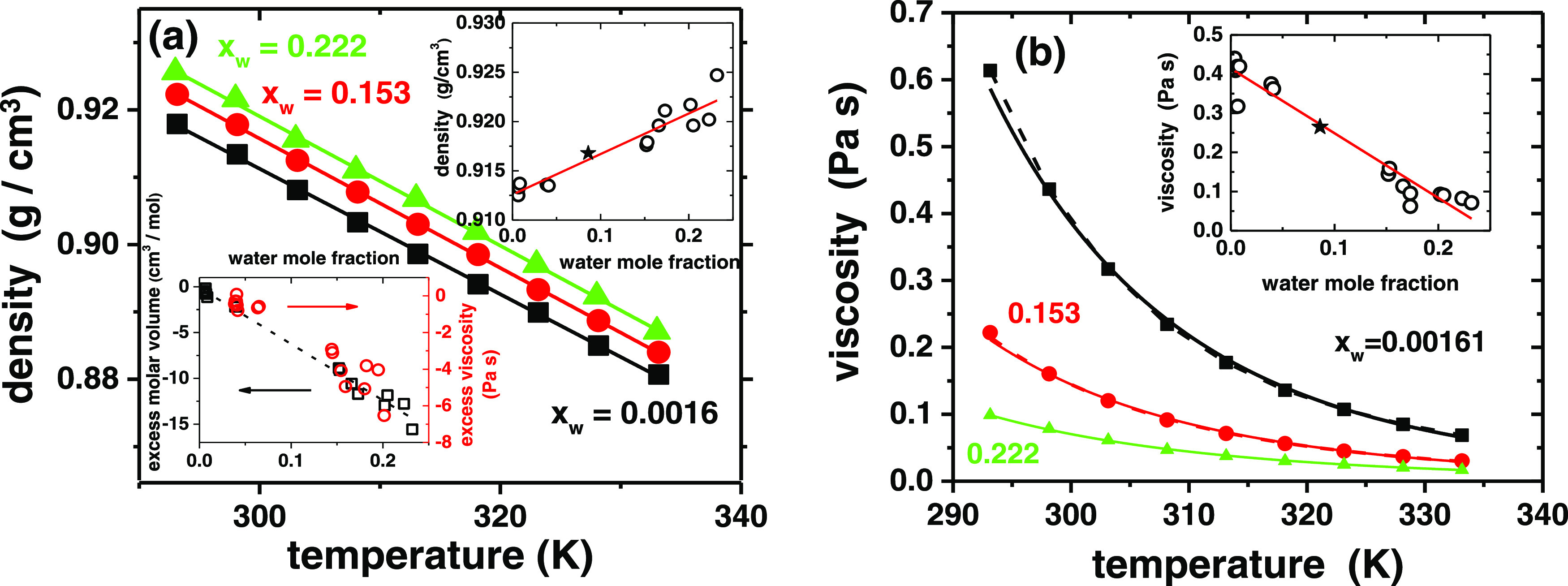
(a) Density and (b) dynamic viscosity (solid and dashed lines are fits to Arrhenius’ law and the VFT equation, respectively) at different water contents as a function of temperature. Insets in panels (a) and (b) show the density and viscosity, respectively, and in panel (a), the excess molar volume and viscosity at 25 °C as a function of water mole fraction (lines are linear fits); (*) is data from van Osch et al.^52^

Density and viscosity of a solvent have a significant effect on other physicochemical properties and potential applications. Figure 5a shows the dependence of density on temperature with three DESs with different water contents. In all cases, the density decreases linearly with temperature with the same slope, and the water content has only a minuscule effect on their thermal expansion coefficient (α = (1.05 ± 0.01) × 10^–3^ K^–1^; Figure S35). Water increases the density of DES approximately linearly within the achievable mole fraction range, which implies that the partial molar volumes of the components do not change markedly with water content. The density predicted by the fit is in excellent accordance with the previously reported value.^52^ The density of the TBAC–DecA DES is clearly lower than that of hydrophilic DESs but comparable to most hydrophobic ones.^1,3,6,52,58^

The viscosities reported in the literature vary widely, but values below ca. 500 mPa s at room temperature can be considered to represent low-viscosity DESs, although values below 100 mPa s are preferable.^1,13,52,58^ The dynamic viscosity of the TBAC/DecA (1:2) DESs with different water contents is shown in Figure 5b as a function of temperature. All samples have relatively low viscosity, comparable to several hydrophilic ones, except the dry solvent at low temperatures. The viscosity generally increases with the size of the DES components, and many hydrophobic solvents containing larger quaternary ammonium cations or large anions have much higher viscosity.^52,58^ However, the comparison with the literature values is complicated because of the marked effect of the water content on the viscosity.^3,41,47,51,58,89−91^ As seen from Figure 5b, the viscosity of the DES studied decreases approximately linearly with the water mole fraction, and again, the linear fit is in excellent accordance with the reported literature value.^52^

Ternary systems of DES and water are not ideal mixtures, and the difference between the observed and ideal thermodynamic properties, an excess quantity, can reveal something about the interactions between the components. The excess molar volume and viscosity for the DES–water mixtures at different water fractions are shown in the inset of Figure 5a (see SI7, eqs S8 and S9). The excess molar volume is negative in all DES–water systems reported and is the largest when the water mole fraction is ca. 0.2–0.3.^51^ However, the excess volume in Figure 5a is larger than with previously reported systems of hydrophilic DESs or ILs, and it changes approximately linearly with the water mole fraction, as expected if the partial molar volumes remain constant. In general, the positive excess volume upon mixing the components of a binary DES (i.e., with no water) is an inspiration for the use of the hole theory of liquids for their description. The free volume formed is considered to consist of holes with a root-mean-square average radius given by 4π⟨*r*^2^⟩ = 3.5 *kT*/γ, where *k*, *T*, and γ are the Boltzmann’s constant, absolute temperature, and surface tension, respectively.^19,22^ The surface tension of the DES formed by the 1:2 ratio of TBAC and DecA is (0.0308 ± 0.0009) N/m at room temperature, independent of the water content (Figure S36), which is in the lower range of the values reported for ILs in the literature.^92,93^ The observed surface tension implies an rms radius of 3.9 Å, large enough to incorporate water molecules in the holes within the solvent structure. However, the theory does not take into account the size and shape of the TBA and DecA molecules, but simulations show a network of irregular cavities, which accommodate water molecules (Figures 4 and S37), and we attribute the large negative excess molar volume to water drawn into these cavities due to strong intermolecular interactions (i.e., interstitially, as suggested for reline).^49^ Similarly, the excess viscosity of water–IL/DES mixtures is usually negative but considerably smaller in the absolute value than observed here.^51^ The large deviation from an ideal behavior implies that water substantially modifies the intermolecular interactions in the DES, as suggested by simulation. The efficient decrease in viscosity even with a very low water content is beneficial to many applications of these solvents.^56^

The temperature dependence of viscosity (Figure 5b) is commonly described by either Arrhenius’ law η = η_0_ exp(*E*_η_/*RT*) or the Vogel–Fulcher–Tammann (VFT) equation η = η_0_ exp[*B*/(*T* – *T*_0_)].^2,13,14^ In the VFT equation, *B* is the fragility parameter and *T*_0_ is the Vogel–Fulcher temperature. In addition to the VFT, other 3-parameter models have also been recently proposed.^94−96^ The term *E*_η_ in Arrhenius’ law can be identified as an activation energy for the viscous flow, and its values for DESs with different water contents are shown in Table 1. They show that water greatly assists the movement of molecules relative to each other. The more viscous the DES, the larger the activation energy, but, interestingly, the activation energies, even in the driest solvent, are within the lower range of those reported for common hydrophilic DESs.^13^ Viscosity of hydrogen-bonding liquids is generally associated with the dynamics of the intermolecular network connectivity.^97^ The simulations show that water in DES interferes with the hydrogen-bonding network, which can be seen as the molecular basis of its effect on the viscosity of the DES, as the rigid hydrogen-bonded network of large molecules becomes more flexible upon the hydration of the components. The simple 2-parameter Arrhenius’ law describes the temperature behavior of viscosity relatively well, but a better description is achieved by the VFT model (Figure 5b and Table S6). The VFT parameters can be used to estimate the fragility *m* of the DES samples (values below ca. 20–25 imply Arrhenius-type behavior).^98^ The DESs with low water content are very fragile, but wet solvent approaches the fragility of typical hydrophilic DESs (Tables 1 and S6).^28^

**Table 1:** Activation Energies for Transport Properties

				*E*_D_ (kJ/mol)^f^		
*x*_w_^a^	*E*_η_ (kJ/mol)^b^	fragility *m*	*E*_κ_ (kJ/mol)^c^	TBA	DecA	DecA(H1)^h^
0.0016	44.4 ± 0.7	98 ± 7	33.2 ± 1.8^d^ (32.2 ± 1.5)^e^	39.3 ± 0.3	41.0 ± 0.7	43.5 ± 0.6
0.153	40.3 ± 0.7	118 ± 8	nd	39.9 ± 0.3	38.4 ± 0.6	53.3 ± 2.7
0.222	36.0 ± 0.4	50 ± 30	29.7 ± 1.7 (29.3 ± 1.5)	nd^i^	nd	nd

a Water mole fraction.

b Activation energy of the viscous flow.

c Activation energy of conductivity (corrected for thermal expansion) according to:

d Arrhenius’ law;

e Modified Arrhenius’ law.

f Self-diffusion activation energy.

h H_2_O + H1 in DecA.

i nd, not determined.

### Diffusion

**Figure 6:**
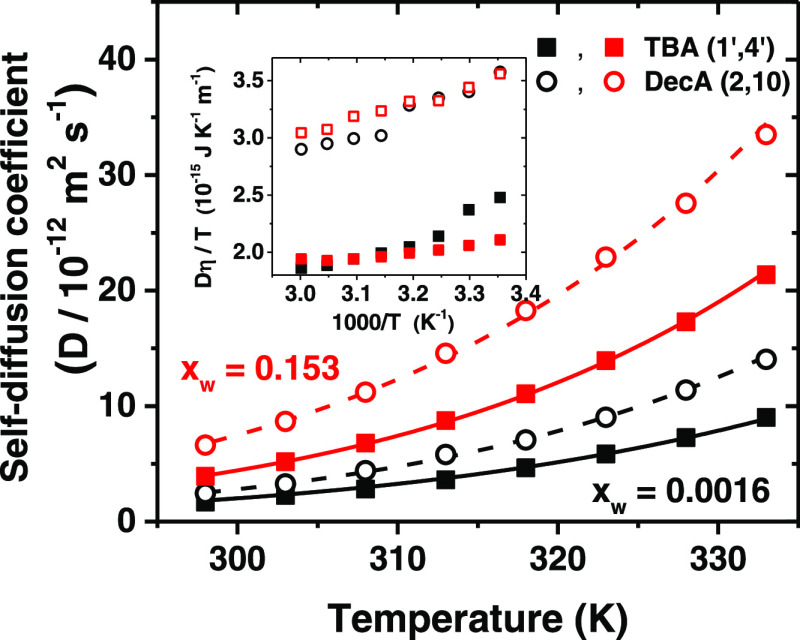
Self-diffusion coefficient of TBA (average of H1′ and H4′) and DecA (average of H2 and H10) as a function of temperature at different water contents. The lines are fits to Arrhenius’ law, and the inset shows the Stokes–Einstein ratio Dη/T as a function of 1/T for TBA and DecA in DES at different water fractions.

NMR is an ideal tool for studying the self-diffusion in liquids. It yields the macroscopic translational self-diffusion coefficients for all distinguishable species in the system. We have used the ^1^H NMR-stimulated echo technique to measure the effective diffusion coefficient of two groups of equivalent protons (H1′ and H4′) in TBA^+^ and three (OH, H2, and H10) in DecA (Scheme 1) in DESs at two different water contents. The protons H1′ and H4′ in TBA^+^ yielded practically identical diffusion coefficients, as did also the protons H2 and H10 (and H1 in the dry DES) in DecA (Figure S39). However, in the wet DES, the COOH proton in DecA showed an anomalously high diffusion coefficient because the signal coincides with that of water. The high increase in the observed diffusion coefficient is in accordance with the behavior reported for exchangeable protons in DESs.^31^ The representative diffusion coefficients have been calculated as averages of the values of protons H1′ and H4′ for TBA (we use TBA here to emphasize that NMR does not allow us to determine whether the values correspond to a TBAC ion pair or a TBA^+^ cation) and protons H2 and H10 for DecA (Figure 6). The diffusion coefficient of DecA is ca. 40–70% higher than that of TBA, and water greatly enhances the diffusion. On the other hand, the diffusion coefficients of TBA and DecA are 1 order of magnitude smaller than those of a smaller choline cation and ethylene glycol in ethaline, a common hydrophilic DES.^23^ Temperature increases diffusion coefficients approximately by 1 order of magnitude in the studied range, and their temperature dependence is well described by Arrhenius’ law. The corresponding diffusional activation energies (*E*_D_) are given in Table 1. The activation energy values for both components are close to each other but that of DecA decreases with water content, implying a smaller need for cooperative arrangement upon translation, while the activation energy of TBA is only slightly affected. The small increase in the latter may reflect the enhanced ion pair formation with chloride when water is present and water penetration within the hydrocarbon sphere, as suggested by simulations. Unfortunately, the very large line width of the ^35^Cl NMR signal, implying variable local environments within the NMR time scale, did not allow the experimental determination of the diffusion coefficient of chloride ions but the literature data and our previous results suggest that the TBA^+^ cation and Cl^–^ anion might migrate together as an ion pair.^13,56^ In the dry DES, the activation energy for the carboxylate proton in DecA was close to the value obtained for protons H2 and H10 but significantly higher in wet DES, where a water signal affects the data. The high activation energy and low value of the effective diffusion constant suggest that a Grotthuss-type mechanism does not markedly contribute to the diffusion of the exchangeable protons.^99^ This is supported by simulations (Figure 4b), which suggest that the water molecules in the DES do not directly network with each other but are hydrogen-bonded mainly to chloride ions and the COOH groups. It has also been previously shown that at low water content the water molecules solvate the chloride anions and become free only at high water content.^31^ At the very low water content of the DES samples studied in this work, water remains tightly bound.^100^

Molecular dynamics offers an alternative view of the diffusion process in DESs (see SI6 and SI9). The trajectories of five different atoms (Cl, N in TBA^+^ and O in water, C=O, and COH) were followed, and the simulated self-diffusion coefficients were obtained from their mean-square displacements (Figures S41 and S42). The simulated values for nitrogen are well in accordance with the experimental results for TBA but show no clear dependence on the water content. The values for the CO and OH oxygens are close to each other (Figure S43), as expected, and increase with water content, but they are markedly higher than the experimental values for DecA. The simulated diffusion coefficients for chlorine are of the same order than those of nitrogen being, however, systematically larger by ca. 25–100%. This suggests that the short-range (see below) diffusion of the TBA^+^ and Cl^–^ ions is, at least partially, decoupled.

Different time scales can explain the differences in the measured and simulated diffusion coefficients. In simulations, the system was followed for 10 ns, whereas the time scale in NMR gradient experiments was approximately 250 ms. Therefore, the simulation probes the localized mobility of the molecules and the NMR technique probes their long-range diffusional transport.^42^ Similarly, quasielastic neutron scattering has revealed two transport phenomena with different time scales in DESs formed from choline chloride and glycerol (glyseline).^42^ The fast process was attributed to a localized motion of a molecule within a cage formed by its neighbors, and the slower process was attributed to its long-range diffusion that requires breaking out of the cage. The corresponding diffusion coefficients differ by ca. 2 orders of magnitude, and the confinement radius of both components was found to increase with temperature, similar to the apparent hydrodynamic radii in this work (Figure S40). In the case of our hydrophobic DES, composed of much larger molecules, the simulated short-range diffusion coefficients of TBA or DecA are ca. 1.5–2 or 5–10 times higher, respectively, than the experimental long-range ones and increase as a function of water content and temperature, indicating increased local mobility.

The Stokes–Einstein equation *D = kT*/*C*η*R*, which binds the diffusion constant (*D*), solution viscosity (η), and the hydrodynamic radius (*R*) of the diffusing species (parameter *C* depends on the boundary conditions), has been shown to be valid under a broad range of conditions, also for molecular species, although it is based on a continuum model for the solvent.^101^ The apparent hydrodynamic radii of TBA and DecA increase with temperature but are practically independent of water content with DecA, whereas with TBA, the behavior is more complex (Figure S40). They are smaller than the hard-sphere radii, which is a common observation, although that of TBA approaches the radius of TBA^+^ (3.29 Å)^30^ at higher temperatures. Abbott et al. have used the correlation length, which describes the decay of structural fluctuations in the medium, instead of the hydrodynamic radius to explain the anomalous diffusion in DESs.^23,102^ They attribute the diffusion in ILs and DESs to a jumping mechanism between voids based on the available free volume per molecule. Other nontrivial explanations of mobility are based on the Adam–Gibbs cooperatively rearranging regions, the fluidized domains, or the random free-energy barrier hopping models.^103−105^ However, generally, any deviation of the ratio *D*η/*T* from a constant value is an indication of a deviation from the Stokes–Einstein relation,^101^ and Figure 6 displays a positive deviation at low temperatures, much larger in the dry DES than in the wet solvent, in accordance with the changes in the apparent radii. It is worthwhile to note that the diffusion coefficients depend linearly on fluidity η^–1^ (Figure S44) but this plot conceals the possible temperature dependence of the hydrodynamic radius. Deviations from the Stokes–Einstein relation are known to occur, especially in supercooled and fragile liquids,^101,106^ and experimental data suggests that the current DES is similar to them. The deviations have been attributed to dynamic heterogeneity in the liquid, which leads to the decoupling of translational diffusion and viscosity.^86,106^ Molecules in regions of rapid dynamics can move large distances, while those in more rigid domains may remain practically immobile during the same time period. In a spatial ensemble average, the rapidly translating molecules dominate and lead to a positive deviation in the self-diffusion coefficient. In glass-forming liquids, this deviation is observed already at temperatures 10–50% above the glass-transition temperature,^107^ and our work, together with reported data for some hydrophilic DESs, shows these solvents to behave like glass-forming liquids.^79^ It is notable that the effect is seen in the dry hydrophobic TBAC–DecA DES already below ca. 310 K. Addition of water does not completely remove the deviation in the temperature range studied but makes it smoother.

### Conductivity

**Figure 7:**
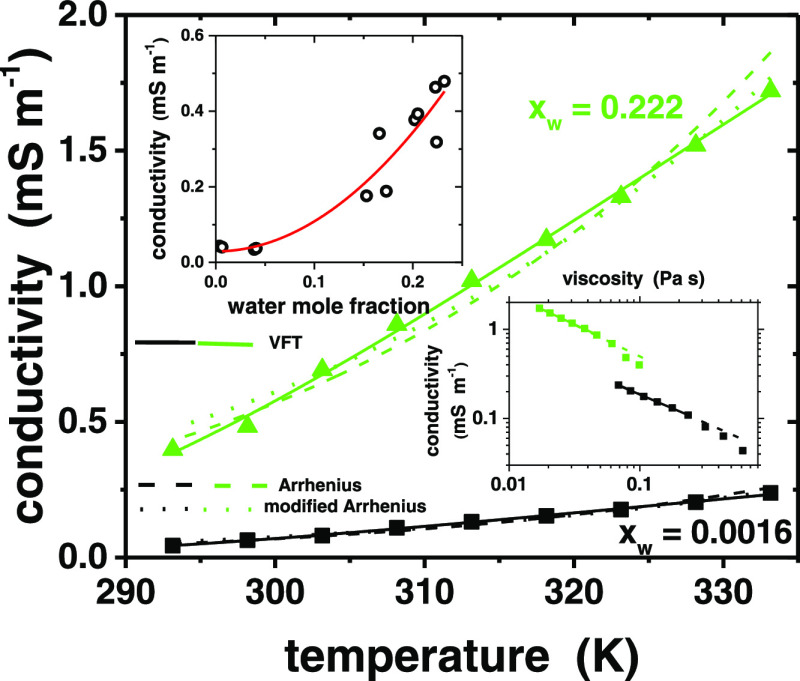
Conductivity of DES (corrected for thermal expansion) as a function of temperature. The lines are fits to different models. The insets show the conductivity at 22 °C as a function of water mole fraction (red curve is a power law fit to data) and the relationship between conductivity and viscosity (linear fits to high-temperature parts shown).

Electrical conductivity is a fundamental transport property, especially important in electrochemical applications. Conductivity depends on both the concentration of the charge carriers and their mobility, and the latter is dependent on diffusion and viscosity. For independent charge carriers with (absolute) charge *z*_*i*_, number density *N*_*i*_, and mobility *u*_*i*_, the total conductivity is given by σ = ∑*z*_*i*_*N*_*i*_*u*_*i*_. Figure 7 shows the temperature dependence of conductivity at 2 water mole fractions, compensated for the dilution of the charge carrier concentration due to thermal expansion. Water clearly enhances ionic conductivity in DES but the effect is nonlinear (inset of Figure 7). The conductivity of ILs and DESs depends on water content in a nontrivial way, and small amounts of water have been shown to greatly increase the conductivity of imidazolium-based ILs.^51,108^ The conductivity changes practically linearly with temperature in the studied range (Figure S45), which has also been observed previously, although a second-order empirical equation has also been used to fit DES conductivity data.^30,109^ If the viscosity followed Arrhenius’ law, and the mobility depended on viscosity by the Stokesian expression *u* = *ze*/*C*η*R*, the conductivity would also be expected to display an Arrhenius-type behavior. However, there is a small deviation of the viscosity from Arrhenius’ law in this case (Figure 5b), and the deviation becomes more obvious in the case of conductivity (Figure 7). In addition, the inset of Figure 7 shows that conductivity and viscosity will become decoupled at temperatures below ca. 310 K. With DESs, a failure of Arrhenius’ law is commonly observed, and the conductivity usually obeys the VFT equation.^20,24,28,110−112^ With ionic liquids, conductivity generally follows the VFT equation as a result of the VFT-type behavior of diffusion and therefore mobility and the weak Arrhenius-like dependence of the charge carrier density.^113^ This is contrary to the studied DES, which displays an Arrhenius’ law dependence for the diffusion coefficients of its components (Figure 6a). A modified Arrhenius’ law ρ = ρ_0_*T*^–1^ exp(−*E*/*RT*) can be used for supercooled glass-forming liquids at low temperatures (actually a Nernst–Einstein equation with constant carrier concentration and Arrhenius’-type diffusion coefficient),^113^ and at higher temperatures, a 4-parameter expression (a product of the Arrhenius’ and VFT equations) is often used.^114^ In fact, assuming constant carrier concentration, the modified Arrhenius’ equation gives a reasonable fit to experimental data (Figure 7). Table 1 shows the activation energies obtained from fits to both 2-parameter Arrhenius’ equations, and the values are similar within the experimental error. They decrease with increasing water content, confirming the general trend that water enhances the transport properties in the DES. However, the best description of the temperature dependence is again given by the VFT model. The conductivity and viscosity depend on each other in a complicated way (Figure 7), which shows that the simplified assumptions behind the commonly used equations are not valid, as suggested for DESs in general.^1^

Several complications can be considered in the case of DESs. First, the number concentration of charge carriers may change with temperature. Changes in the ion pairing between TBA^+^ and Cl^–^ could affect the carrier density and mobility, but simulations suggest that temperature has a negligible effect on either the N–Cl distance or the coordination number of chloride around nitrogen (Figure S24). Dissociation of DecA could be an important factor because of the high mobility of protons, but the equilibrium concentrations in all chemical processes should display an Arrhenius-type temperature dependence. On the other hand, simulations imply (Figure S22) that the association of chloride and carboxylic acids increases with temperature, which should decrease the effective mobility of chloride, and this factor might qualitatively explain the apparent linear temperature dependence of conductivity, although its quantitative assessment is not possible. Second, the observed conductivity is the sum of contributions of all carriers, and their combined effect will not adhere to a simple functional form if the appropriate parameters (e.g., activation energies) are not close to each other. Even though the Arrhenius-type fitting of the conductivity data is not very good, the obtained activation energy values suggest that the charge carriers are more mobile than those species for which diffusion coefficient could be obtained by NMR. In the dry DES, the chloride ion should be the majority carrier, but when water is present, the aqueous nanophase will be acidic because of DecA. In this case, protons and the chloride ions released from the native hydrogen-bonding network in the DES will both contribute to the higher observed conductivity. However, the proper identification of the charge carriers and the assignment of their roles would require frequency-dependent studies. Third, the decoupling between conductivity and viscosity (Figure 7), on the one hand, and diffusion and viscosity (Figure 6), on the other hand, occurs in the same temperature range (ca. 310 K). This suggests that the temperature dependence of conductivity is regulated by the same factors as other transport phenomena. The VFT equation gives the best description of the temperature dependence of conductivity, which suggests the importance of cooperative motions in the solvent.^28^ Interestingly, the Vogel–Fulcher temperatures (*T*_0_) for the wet and dry DES samples (243 ± 10 and 247 ± 5 K, respectively), below which the cooperative motions should cease, are close to the first observed endothermal processes in the DSC, attributed to melting (Figure 1).

### Local Dynamics

The study of the nuclear spin relaxation processes yields information about the local molecular dynamics at the nanosecond time scale.^115^ In liquids, the spin–lattice relaxation due to a locally fluctuating magnetic field is caused by the dipole–dipole interactions or chemical field anisotropy and, in the absence of quadrupole nuclei, the dipole–dipole coupling is normally the most important factor. In this case, the ^13^C *T*_1_ relaxation time dependence on the rotational correlation time τ_c_ is given by the Bloembergen–Purcell–Pound theory (BPP; see eq S15) by 1/*T*_1_ = *A*_0_*Z*(τ_c_), where *Z*(τ_c_) is a second-order function of τ_c_, and *A*_0_ depends only on the local geometry and the properties of the nuclei.^115−117^ The rotational correlation time measures the rate at which the correlation between the molecular orientations decays with time and can roughly be identified as the time the molecule needs to rotate approximately 1 radian. The most effective relaxation occurs when the field fluctuations occur approximately at the nuclear Larmor frequency, corresponding to the maximum of the function *Z*(τ_c_). The T_1_ minimum, if observed, can be used to calculate the factor *A*_0_ and the correlation times for that nucleus as a function of temperature.

**Figure 8:**
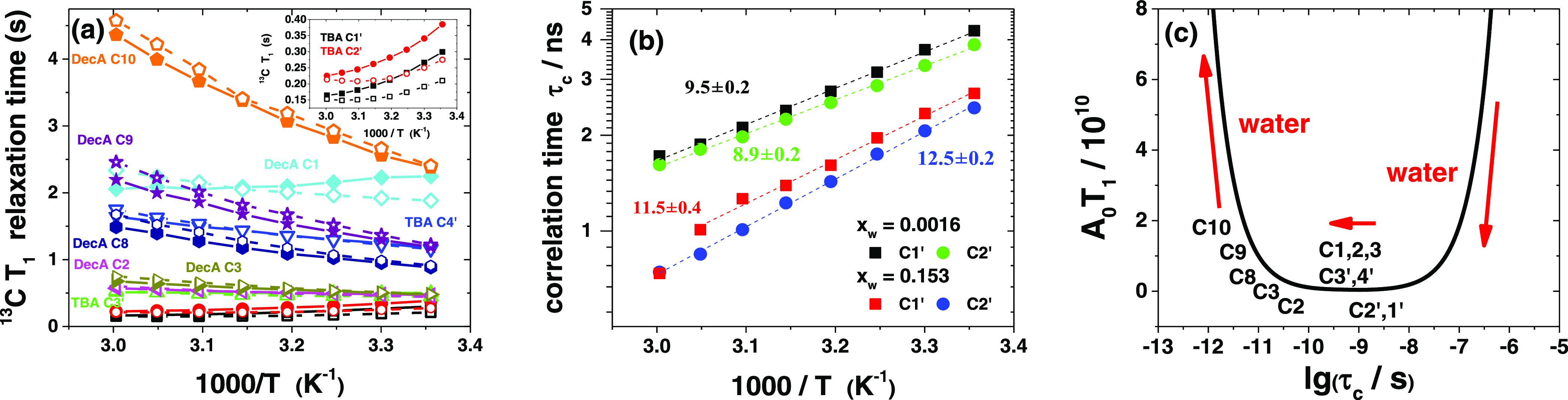
(a) ^13^C spin–lattice relaxation times (*T*_1_) in a 1:2 TBAC/DecA DES as a function of temperature; atom assignments are shown in figures, lines as a guide to the eye (solid lines and symbols, *x*_w_ = 0.0016; dashed lines and open symbols, *x*_w_ = 0.153); the inset shows a close-up for carbons C1′ and C2′; (b) rotational correlation time (τ_c_) for TBA carbons (1′ and 2′) at different water mole fractions; linear fits with activation energies (kJ/mol) shown; and (c) a schematic presentation of the effect of water on the rotational correlation times (calculated correlation time for the C1′ and C2′, location of other nuclei approximate).

The ^13^C *T*_1_ relaxation times are shown in Figure 8a as a function of temperature. A minimum is only seen with the C1′ and C2′ carbons of TBA in the wet solvent (Figures 8a and S48) but, because the factor *A*_0_ depends only on the C–H geometry around the carbon nucleus, the same values can be used for the dry and wet solvents. The resulting equation has two roots but the physically meaningful solution must be a monotonous function of temperature.^118^ The rotational correlation times calculated for the carbon nuclei C1′ and C2′ in TBA are depicted in Figure 8b (see SI11 for details), showing that the correlation times exhibit an Arrhenius-type behavior, in accordance with the absence of decoupling between rotational motion and viscosity.^106^ These local motions do not require cooperational movements of many molecules or breaking out of the cage formed by the neighboring molecules. The correlation times for C2′ are a little lower than those of C1′ in the dry and wet solvents, which may be attributed to enhanced segmental mobility at the C2′ position. This conclusion is supported by the orientational correlation decay of the C3′–C4′ vector, which is faster than that of the C2′–C3′ vector (Figure S51). Water enhances the local dynamics, and the correlation times decrease approximately by a factor of 2 in the wet DESs. The activation energy for rotational motion is lower than observed in imidazolium-based ILs but the correlation times are longer, especially in the dry solvent.^118^ The increase of activation energy in the wet solvent can be tentatively attributed to a higher amount of hydrogen bonds that have to be disturbed to allow rotation.

Water has only a small effect on the ^13^C relaxation times, and it depends on temperature and the carbon atom (Figure 8a). Because the BPP theory predicts a U-shaped functional dependence between *T*_1_ and τ_c_, we can qualitatively discuss the effect of water also in those cases where no minimum is observed (Figure 8c; see SI11 for details). These considerations clearly show that water enhances local dynamics in all cases, which suggests that the ^13^C relaxation times are mostly dependent on the rotational motion of the whole molecule. However, the effect of segmental mobility becomes more pronounced in TBA for carbons close to the chain ends. In addition, with DecA, the segmental mobility increases with distance from the COOH group. This implies that the carboxylic group is anchored due to intermolecular interactions while the chains are more mobile.

None of the ^1^H *T*_1_ relaxation times shows a minimum in the temperature range studied, but, contrary to the ^13^C relaxation times, water considerably lowers the relaxation time in all cases (Figure S52). Similar considerations using the BPP theory suggest that the addition of water hinders the local dynamics in all cases except for the H1′ proton in TBA, which is the most shielded proton with the longest correlation time. This suggests that segmental and other local motions may play a significant role in determining the ^1^H *T*_1_ relaxation times. Water molecules penetrating inside the TBA hydrocarbon sphere probably attenuate these motions, thereby increasing the correlation time (Figure S52b). Therefore, more reliable rotational information is obtained from the ^13^C data, and the behavior of the ^14^N T_2_ relaxation times (Figures S54 and S55) supports the conclusions drawn from the ^13^C relaxation data.

### Effect of DES Stoichiometry

**Figure 9:**
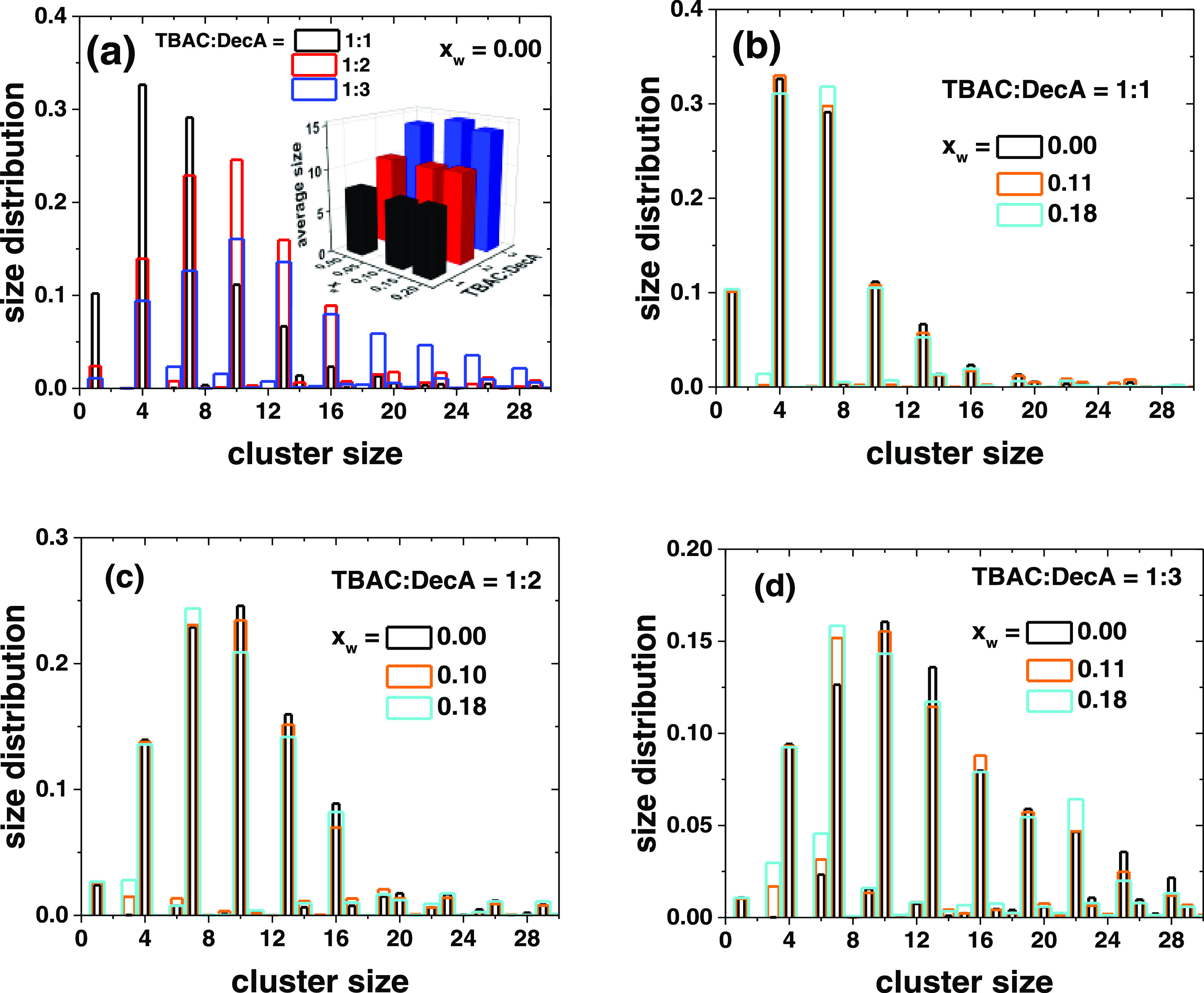
Cluster size analysis of the Cl*_m_*···(HOO)*_n_* aggregates as a function of (a) the TBAC/DecA ratio (dry mixtures) and the water content in the (b) 1:1, (c) 1:2, and (d) 1:3 DES compositions. Unequal bar widths for clarity. The inset in panel (a) shows a three-dimensional (3D) graph of the average cluster size.

**Figure 10:**
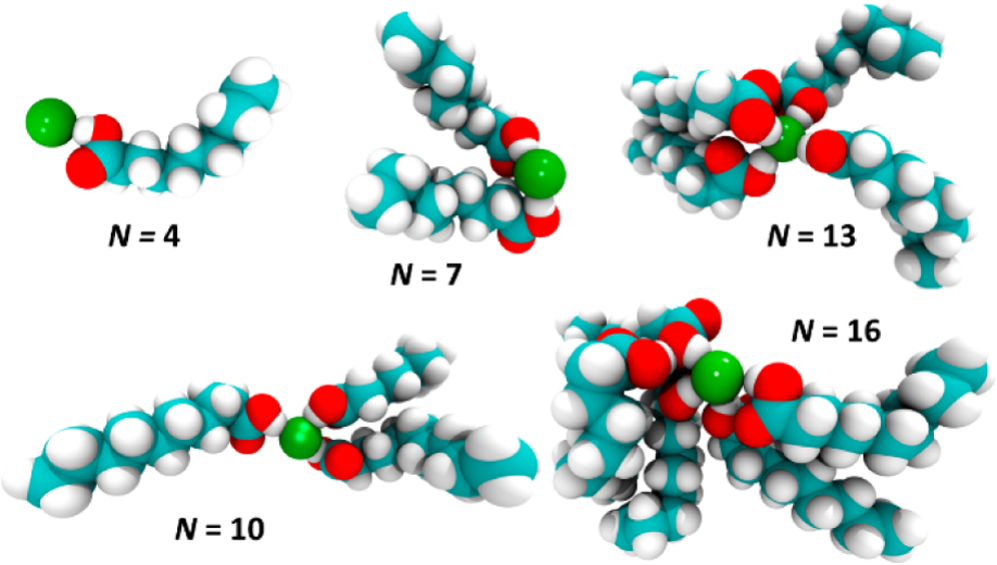
Snapshots of clusters of different sizes; color coding: C (blue), H (white), O (red), and Cl (green). Note that for *N* = 16, one DecA is not directly hydrogen-bonded to Cl.

DESs depend on strong intermolecular interactions, and hydrogen bonding between a halide anion and a HBD is considered essential for their formation.^5,13^ The results presented above have shown that added water interferes with this fundamental interaction but it does not destabilize the solvent because of new interactions between the components. The close association of chloride ions and carboxylic acid moieties reflects the important stabilizing interactions, and we have used cluster analysis to see how it is affected by the TBAC/DecA ratio, varied from 1:1 to 1:3, and the water content. The cluster is formed by the hydrogen and two oxygen atoms in the carboxylic acid groups and the chloride anions if they can be connected by steps less than 3.5 Å from atom to atom. The cluster size is the number of such atoms and is given by *N* = 3*n*__COO__ + *n*_Cl_, where *n*_COO_ and *n*_Cl_ are the number of carboxylic acid groups and chloride ions in the cluster, respectively. The cutoff distance was chosen large enough to include all chloride–HO hydrogen bonds.^82,83^ In aqueous solutions, a chloride ion can be hydrogen-bonded to at least 4, possibly to even more water molecules.^119,120^Figure 9a shows the comparison of dry mixtures of different TBAC/DecA ratios. In the 1:1 mixture, small clusters (*N* = 4 and 7, corresponding to Cl···HOO and Cl···(HOO)_2_ clusters) prevail because of the shortage of carboxylic groups. Specifically, there are many free chloride ions (*N* = 1) not associated with the carboxylic groups, but all COOH groups are bound to chlorides (no clusters with *N* = 3 or 6). A change of the ratio to 1:2 or 1:3 increases the amounts of large clusters with 10, 13, and even 16 atoms. The first is chloride associated with three carboxylic acid groups, and the second represents Cl···(HOO)_4_, but the identification of the larger clusters is not unambiguous. Snapshots from the simulations suggest Cl···H–O distances in the range of 1.9–2.05 Å and angles close to 180°, in accordance with the literature (Figures 10 and S57; see SI12 for other suggested structures).^82,83^ The largest clusters are seen with the TBAC/DecA ratio 1:3, which implies that the eutectic composition may not be very strictly determined, which we attribute to the ability of chloride to hydrogen-bond with several DecA molecules. In fact, different TBAC/DecA stoichiometries have been reported;^121−123^ some DESs show rather wide eutectic minima,^50,59,109^ and a strict stoichiometric proportion is often not a good characteristic of a DES.^4^ The addition of water has very little effect for the 1:1 mixture, but in the 1:2 and 1:3 mixtures, it clearly breaks down larger aggregates and favors the smaller ones, the more the higher the water content (Figure 9b–d). DecA and water have somewhat controversial roles as the increase of the water or DecA fraction both affect the viscosity similarly.^123^ With the 1:2 and 1:3 stoichiometries, the 7-membered cluster Cl···(HOO)_2_ becomes the most important one with water, but we see also individual carboxylic acid groups (*N* = 3) and their dimers (*N* = 6). This is in line with the results from the RDF curves (Figure 2), showing that water competes with chloride for hydrogen bonding with the COOH groups and enhances carboxylic acid self-correlation.

## Conclusions

The water contamination in hydrophobic deep eutectic solvents is generally due to atmospheric absorption, and a low water content is unavoidable when working under ambient atmosphere. This work focuses on the effect of very low concentrations of water in a prototypical hydrophobic DES formed by a 1:2 mixture of tetrabutyl ammonium chloride (TBAC) and decanoic acid (DecA), but the results have wider implications for the use of hydrophobic DESs in applications. Even though the water content is limited to low values (below ca. 2.5 wt % or 0.2 mole fraction), its effects are significant in the hydrophobic solvent. Deep eutectic solvents, even the hydrophobic ones, are in practice ternary mixtures, unless strict precautions are applied to avoid water absorption, and the water content is one of the major reasons for differences in the values reported. In the mole fraction range studied (*x*_w_ < 0.22), the density, viscosity, and the DES–water ternary eutectic melting point change approximately linearly with water content but the electrical conductivity exhibits a nonlinear dependence because of changes in the charge carriers.

Molecular dynamic simulations show the importance of hydration on the chloride–carboxylic acid hydrogen bonding and allow us to identify structural clustering. In dry DES, chloride is bound to DecA by hydrogen bonding and forms ion pairs with TBA^+^ due to the Coulombic interactions. These intermolecular interactions form a network, which restricts the motions of the molecules at different scales. Water breaks the interactions between DecA and chloride and enhances the ion pair formation between Cl^–^ and TBA^+^. Water hydrates the carboxylic acid groups and chloride ions, which leads to a dynamic nanoscale phase segregation. The analysis also suggests that the composition range of the liquid solvent is relatively wide due to the high hydrogen-bonding capacity of the chloride ion.

The water-induced local changes in structure and dynamics enhance local and long-range mobility and lead to a decrease in viscosity and increase in diffusion, while the formation of new charge carriers contributes to the increase in conductivity. A decoupling of all long-range dynamic properties takes place at approximately the same temperature (ca. 310 K). The decoupling between the transport properties is typical for fragile glass-forming liquids, and the studied hydrophobic DES is a very fragile solvent, which can easily be supercooled to a metastable state. Water in the DES softens the decoupling phenomena and decreases the fragility of the solvent. Local dynamics, on the other hand, is not affected by the decoupling. The ^13^C relaxation data is the best indicator of local dynamics and shows that the different parts of the components have different degrees of local dynamical freedom.

In summary, very small water contamination in a hydrophobic DES brings about similar changes as observed in hydrophilic DESs with much higher water content. From the point of view of applications, even a very small water content in a hydrophobic DES has a beneficial effect on its physical properties, especially on the transport properties, i.e., global dynamics. On the other hand, water may compromise the chemical properties of the solvent. The chloride ions are partially freed from the hydrogen bond network, and the solvent becomes corroding due to its acidity and high halide ion concentration in the aqueous nanophase. This may hamper the use of the aqueous DES as a solvent in some organic synthesis and other applications, and the role of water should always be considered.

## Supporting Information Available

DES preparation, mole fraction calculation, details of experimental techniques and modeling, all radial distribution functions, UV–vis, IR, Raman, and NMR spectra, detailed data analysis of density, viscosity, fragility, surface tension, diffusion coefficient, conductivity, and relaxation time measurements, details of cluster analysis, references for SI, and force field parameters used (PDF)
