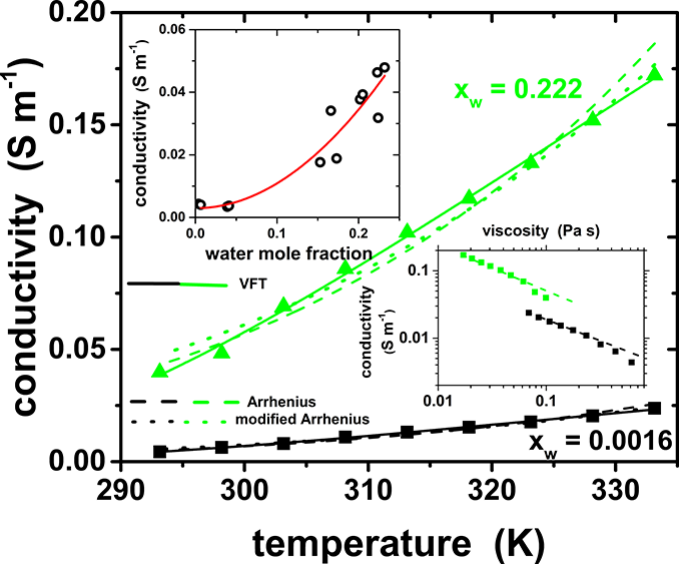# Correction to “The Effect of Water on a Hydrophobic Deep Eutectic Solvent”

**DOI:** 10.1021/acs.jpcb.2c01318

**Published:** 2022-03-10

**Authors:** Henri Kivelä, Mikko Salomäki, Petteri Vainikka, Ermei Mäkilä, Fabrizio Poletti, Stefano Ruggeri, Fabio Terzi, Jukka Lukkari

The conductivity axis in Figure 7 of the paper (and Figures S45 and S46 in the Supporting Information) was erroneous, and the correct figures are shown here and in the updated SI file; the figure captions remain the same. These corrections do not in any way affect the discussion and conclusions presented in the paper.

**Figure 7:**