# Supplementary material for: Correction to “The Effect of Water on a Hydrophobic Deep Eutectic Solvent”
Source: J Phys Chem B. 2022 Mar 10;126(11):2324. doi: 10.1021/acs.jpcb.2c01318 (PMC9060418; doi:10.1021/acs.jpcb.2c01318)
Supplement: Supplementary file 1 — jp2c01318_si_001.pdf [file jp2c01318_si_001.pdf]

Supporting Information for

The Effect of Water on a Hydrophobic Deep Eutectic Solvent

Henri Kivelä [‡,§]; Mikko Salomäki [‡,§]; Petteri Vainikka [‡,†]; Ermei Mäkilä [◊,ǁ]; Fabrizio Poletti [⁋,]; Stefano Ruggeri [⁋]; Fabio Terzi [⁋,†]; Jukka Lukkari [‡,§,*]*

‡ Department of Chemistry, University of Turku, FI-20014 Turku, Finland

◊ Department of Physics and Astronomy, University of Turku, FI-20014 Turku, Finland

§ Turku University Centre for Surfaces and Materials (MatSurf), FI-20014, Turku, Finland

ǁ Doctoral School for Chemical and Physical Sciences, University of Turku, FI-20014 Turku, Finland

⁋ Electrochemical Sensors Group, Department of Chemical and Geological Sciences, University of Modena and Reggio Emilia, Via Giuseppe Campi, 103, I-41125, Modena, Italy

[†] Present Addresses:

PV, Zernike Institute for Advanced Materials, University of Groningen, Nijenborgh 4, 9747 AG, Groningen, Netherlands, and Groningen Biomolecular Sciences and Biotechnology Institute, University of Groningen, Nijenborgh 4, 9747 AG, Groningen, Netherlands;

FT, SICER S.p.a., Via Montegrappa 4/18, I-41042, Modena, Italy

* Corresponding Authors:

Jukka Lukkari, Department of Chemistry, University of Turku, FI-20014 Turku, Finland, https://orcid.org/0000-0002-9409-7995; email: jukka.lukkari@utu.fi

Fabrizio Poletti, Electrochemical Sensors Group, Department of Chemical and Geological Sciences, University of Modena and Reggio Emilia, Via Giuseppe Campi, 103, I-41125, Modena, Italy, https://orcid.org/0000-0001-9646-021X; email: fabrizio.poletti@unimore.it

Contents

1. DES preparation and water saturation S3
    1.A. Calculation of the water content S3

2. Experimental details S4

3. IR, Raman, and NMR spectra S8

4. Water saturation equilibrium S11

5. Differential scanning calorimetry S12

6. Molecular modelling S14
    6.A. General S14
    6.B. Radial distribution functions S16
        Water self-correlation S18
        Water – Cl correlation S22
        OH – Cl correlation S25
        N – Cl correlation S27
        DecA self-correlation S30
        DecA chain self-correlations and potentails of mean force S32

7. Thermal expansion, excess quantities, and surface tension S35

8. Viscosity and fragility S38

9. Self-diffusion coefficients: measurement and modelling S40

10. Conductivity S44

11. Local dynamics from NMR relaxation times S45
    T1 relaxation times S45
    T2 relaxation times S49

12. Cluster analysis S52

13. References S54

Appendix: GROMACS compatible molecular topologies and starting structures S60

1. DES preparation and water saturation

TBAC, the HBA, was weighted in an Erlenmeyer flask; then, DecA, the HBD, was added. Then, the flask was heated in a water bath at 65°C for three hours, periodically mixing the content, until the mixture turned to a homogeneous, clear and colorless fluid. The walls of the flask were rinsed with the liquid in order to ensure that all the weighted solid was in the liquid phase. Figure S1 shows a typical experimental setup, with the water bath used.

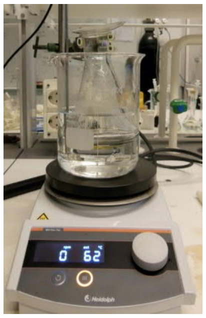

Figure S1. Experimental setup: water bath used to synthesize the DES.

As previously reported, water is a primary component in these solvents. To highlight differences related to the water content, DESs have been synthesized with the procedure described above, adopting however different conditions. Three sets of samples were prepared:

- DESs prepared in ambient conditions, in equilibrium with the ambient atmosphere of known temperature
- DESs prepared inside the glovebox, with water and oxygen only present in traces (< 4 ppm).
- DESs prepared in ambient conditions, then placed in a water-vapor saturated atmosphere at different temperatures (from 30 °C to 60 °C) for several hours in order to equilibrate the solvent with water vapor.

These different synthetic procedures developed allowed to change the water content of the DES.

1.A. Calculation of the water content

For a bicomponent DES with a composition AB_f we have $n_B = fn_A$ (Note: f need not be an integer). If the water-% of the DES (weight-%) is $w\%$ the, the water mole fraction is then given by

$$x_W = \frac{n_W}{n_W + n_A + n_B} = \frac{w\%}{w\% + (100 - w\%)(f+1)\frac{M_W}{(M_A + fM_B)}} \quad \text{(S1)}$$

which, in this particular case ($f = 2$), gives (w% is given in % here)

$$x_W = \frac{w\%}{0.9132w\% + 8.6825} \quad \text{(S2)}$$

2. Experimental details

Density was measured with an Anton Paar DMA 45 densimeter calibrated with air and ultrapure water, ***viscosity*** with an Anton Paar AMVn Automated Microviscometer equipped with a Peltier thermostat, and electric ***conductivity*** by using an Orion 150Aplus conductivity cell (Thermo Electron Co., cell constant was calibrated with solutions of known conductivity), all at several temperatures. ***Surface tension*** measurements were done using a hanging drop method with an Attension Theta Optical Tensiometer (Biolin Scientific). In ***differential scanning calorimetry*** (DSC, Pyris Diamond DSC, Perkin Elmer) the sample was placed on an aluminum DSC pan (Perkin Elmer, capacity of 30 µl). ***Laser scattering*** experiments were performed using a Zetasizer Nano ZS instrument (Malvern). ***IR spectra*** have been acquired using a Bruker Vertex 70 FT-IR spectrometer, equipped with BR4 Diamond attenuated total reflection (ATR) accessory (Harrick) and a RT-DLaTGS detector. ***Raman spectra*** were measured with a Qontor inVia confocal Raman Microscope (Renishaw) using 532 nm excitation. All experiments have been carried out at ambient pressure.

The NMR measurements were carried out with a Bruker Avance-III 500 MHz NMR spectrometer and processed using the TOPSPIN 3.5 software (Bruker). The 1D ^{1}H NMR spectra of the DES preparations were measured with a standard Bruker pulse–acquire sequence "zg30", using a 3.33 µs 30° pulse, 3.3 s acquisition time, and 1.0 s relaxation delay (d1). The 1D ^{13}C NMR spectra were recorded using the sequence "zgpg30" with a 3.33 µs 30° excitation pulse, 1.1 s acquisition time, 2.0 s relaxation delay, and "waltz16" proton decoupling. The 1D ^{14}N NMR spectra were measured using the sequence "zgig" with inverse-gated "waltz16" proton decoupling (decoupling on only during acquisition), using a 10.0 µs 90° excitation pulse, 2.3 s acquisition time, and 6.0 s relaxation delay. No internal chemical shift reference was added to the DES mixtures in order to avoid changing their composition. Instead, the decanoic acid (DecA) methyl protons (denoted H10 in the manuscript) were used for ^{1}H shift referencing, for which the value δ_{H10} = 0.88 ppm was assigned based on literature.[1] The ^{13}C and ^{14}N NMR spectra were then calibrated by using IUPAC's unified referencing scale[2] : the absolute frequencies for their 0-ppm positions were calculated by using Ξ_{13C} = 25.14502, Ξ_{14N} = 7.226317 (these correspond to a TMS and CH_3NO_2 reference, respectively).

The ***self-diffusion coefficients*** D of TBAC and DecA in DES samples at different temperatures (25–60 °C, 298–333 K) were measured with a stimulated echo ^{1}H NMR method by using Bruker's pulse program "ledbpgp2s". During the pulse sequence, pulsed magnetic field gradients G (tesla/m) are applied across the sample in *z*-direction (along the NMR tube axis) as shown on the G_z channel in Figure S1. The result of this diffusion measurement is a ^{1}H NMR spectrum in which the intensity (or integral) I_i of each ^{1}H peak i is attenuated proportional to the applied gradient strength G and the diffusion coefficient D_i of the protons of peak i. The applied gradient strength G was varied and the intensity I of the peak in the ^{1}H spectrum was fitted to the Stejskal-Tanner equation (S1)

$$I = I_0 \exp\left[-DG^2\gamma^2\delta^2\left(\Delta - \frac{\delta}{3}\right)\right] \quad \text{(S3)}$$

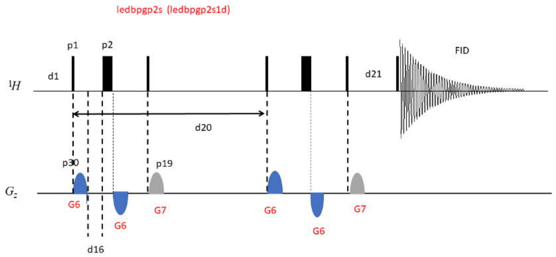

Figure S2. The stimulated echo ^{1}H NMR pulse sequence used in this work for measuring the self-diffusion coefficients. See text for explanations.

Here γ is the magnetogyric ratio of proton ($2.675 \cdot 10^8\,\mathrm{s}^{-1}\mathrm{T}^{-1}$), δ the total duration of the bipolar pulse pair, and Δ the diffusion time. The parameter δ is related to the gradient pulse duration, $\delta = 2 \cdot p30$ (it is the total duration of the bipolar gradient pair G6 and –G6 appearing twice in the pulse program). The diffusion time Δ is the time given to the molecules to diffuse during the pulse program. It is roughly the time between the dephasing gradient pair at the start and the rephasing gradient pair at the end of the program, $\Delta = d20 - d16/2$. The time parameters $d16, d20, p30$ are set by the user during the optimization of the diffusion experiment, and are thus known in the above equation. In this work, the delays were d20 = 250 ms, d16 = 0.5 ms, and the duration of the gradient pulse G6 (p30) was 6 ms for the wet and 11 ms for the dry DES mixture. In practice, the diffusion experiment is repeated multiple times at increasing values of G (in this work, 32 times). According to the equation above, a Gaussian-type decay $I_i \propto \exp(-c_i \times G^2)$ of each peak *i* is seen. The parameter c_i may be obtained from a Gaussian fit, from which the diffusion coefficient D_i for each peak i is obtained ($c_i = D_i\gamma^2\delta^2[\Delta - \delta/3]$). The diffusion datasets were processed by using Bruker's TOPSPIN 3.5 software. The diffusion measurement yields a "pseudo-2D" data matrix with 32 rows: each row corresponds to a ^{1}H NMR spectrum at different G and consists here of 64k intensity points. TOPSPIN's "T1/T2 module" may be used to integrate the user-defined ^{1}H peaks *i* in the diffusion pseudo-2D dataset as a function of G and to perform a Gaussian fit on the resulting I_i vs. G data for the calculation of the diffusion coefficient D_i for each user-defined ^{1}H peak.

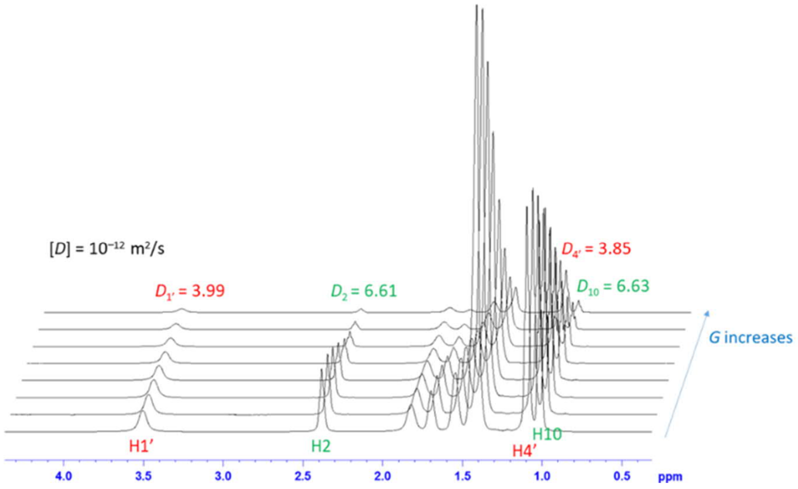

Figure S3. Fourier-transformed data set obtained with a ^{1}H stimulated echo diffusion experiment ledbpgp2 for the wet DES (x_{W} = 0.153) at 298 K, together with the ^{1}H peak assignment (cf. Scheme 1) (red: TBA, green: DecA) and the fitted diffusion coefficients *D*. Only the chemical shift range 0.3–4.4 ppm and only 8 out of the 32 *G*-increments are shown.

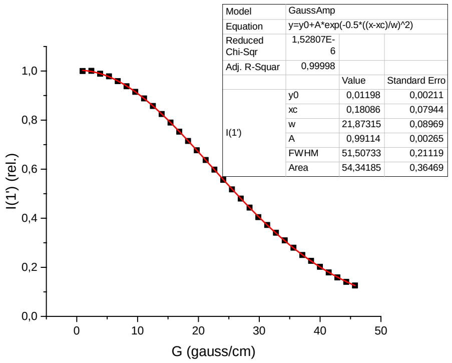

Figure S4. Example of Gaussian data fitting. Integral of the proton 1’ of TBA peak in the wet DES sample at 298 K.

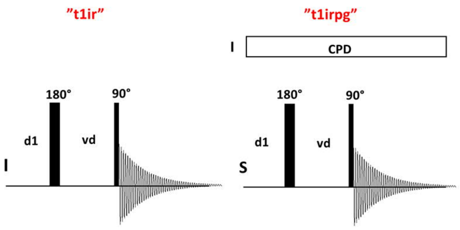

Figure S5. Pulse programs for the measurement of the ^{1}H (left) and ^{13}C (right) T_1 relaxation times. See text for explanation. I, S = rf channels for H and C, respectively, CPD = Composite Pulse Decoupling.

The ^{1}H and ^{13}C T_1-relaxation times of the DES samples, at temperatures 25–60 °C (298–333 K), were measured with a simple inversion recovery pulse sequence (Bruker pulse program "t1ir") in case of protons and with an inversion recovery sequence using power-gated composite-pulse (waltz16) ^{1}H decoupling (Bruker pulse program "t1irpg") in case of carbons. Proton T_1-times were measured by using a total of 18 variable-delay times (vd) (0.001, 0.005, 0.010, 0.020, 0.050, 0.070, 0.100, 0.150, 0.200, 0.300, 0.350, 0.400, 0.500, 0.700, 1.00, 2.00, 4.00, and 8.00 seconds), with an acquisition time of 1.6 seconds and a recycle delay (d1) of 10.0 seconds. For carbon T_1-measurements, 16 vd's were used (0.02, 0.04, 0.08, 0.14, 0.22, 0.32, 0.45, 0.60, 0.75, 0.90, 1.20, 1.70, 3.00, 5.00, 10.0, and 15.0 seconds), with an acquisition time of 1.1 seconds and a d1 of 15.0 seconds. The repetition time of the successive scans (d1+vd+aq) should be at least 5 times the highest T_1 in order to avoid saturation effects and to yield reliable T_1 values. The set of vd's should be "dense" in the time range where most of the relaxation takes place (0 – $2T_1$), while the longest vd should be several times the T_1 which is measured.
The integrals of the ^{1}H and ^{13}C NMR peaks as a function of vd (= t) were fitted to an exponential function with a single time constant (T_1) by using TOPSPIN-software's T1/T2-module:

$$I_i = I_{i,\infty} + P_i e^{-t/T_{1,i}} \qquad \text{(S4)}$$

Examples of the T_1 relaxation plots for the TBA N–CH_2 protons and carbon (i = H1' and C1') in wet DES (x_W = 0.153) at 298 K are shown in Fig. S5 below.

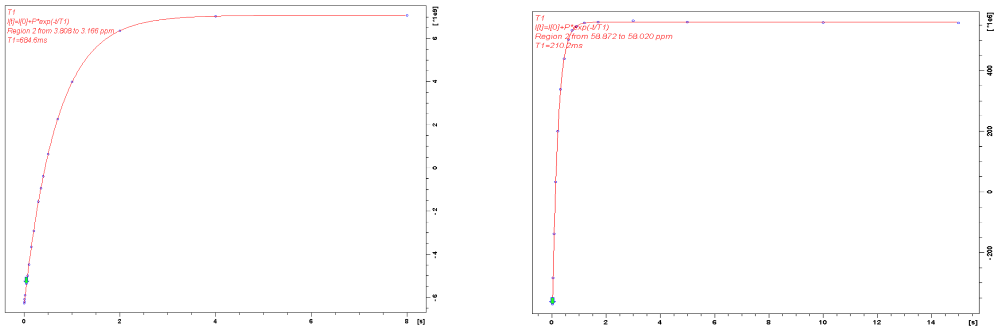

Figure S6. Left, T_1 relaxation of the N–CH_2 protons (H1') of TBA in DES at 298 K, fitted T_1 = 0.685 s; right, T_1 relaxation of the N–CH_2 carbon (C1') of TBA in DES at 298 K, fitted T_1 = 0.210 s. Water mole fraction in DES is 0.153.

3. IR, Raman, and NMR spectra

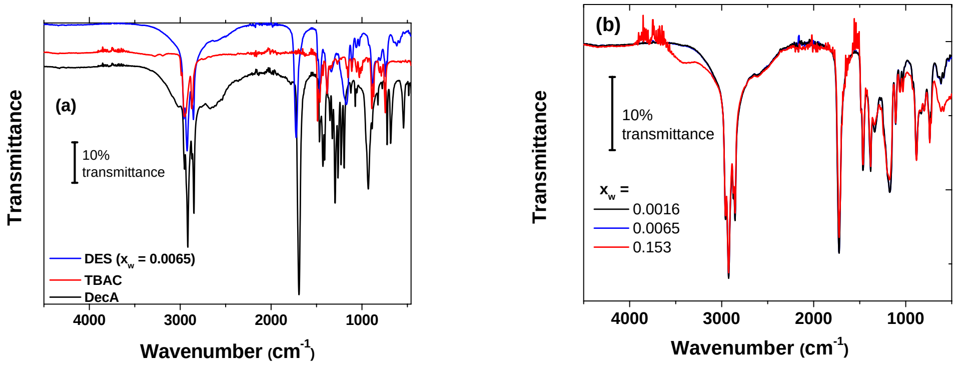

Figure S7. (a), ATR-FTIR spectra of DecA, TBAC, and DES prepared in ambient atmosphere (x_w = 0.0065); (b), superimposition of the IR spectra of DESs with different water contents.

IR spectra were acquired using a Bruker Vertex 70 FT-IR spectrometer, equipped with BR4 Diamond attenuated total reflection (ATR) accessory (Harrick). A room temperature deuterated L-Alanine doped triglycine sulfate (RT-DLaTGS) detector was used. The spectra were measured using both liquid and solid samples without any pretreatment. Adherence to the diamond crystal was controlled with a camera.

The C=O vibration (ca. 1700 cm^{-1}) is shifted higher in DES with respect to DecA by ca. 30 cm^{-1}, in opposite direction than reported earlier.[3] No peaks related to the stretching or the bending of the O-H bond, at ca. 1050 and 650 cm^{-1}, respectively, or peaks typical of the carboxylic acid dimers (at ca. 1400, 1300 and 900 cm^{-1}, and its overtones over 2400 cm^{-1}).[4] Water has negligible effect on the spectra, except for the appearance of a wide band around 3500 cm^{-1} (OH vibrations) at high water content.

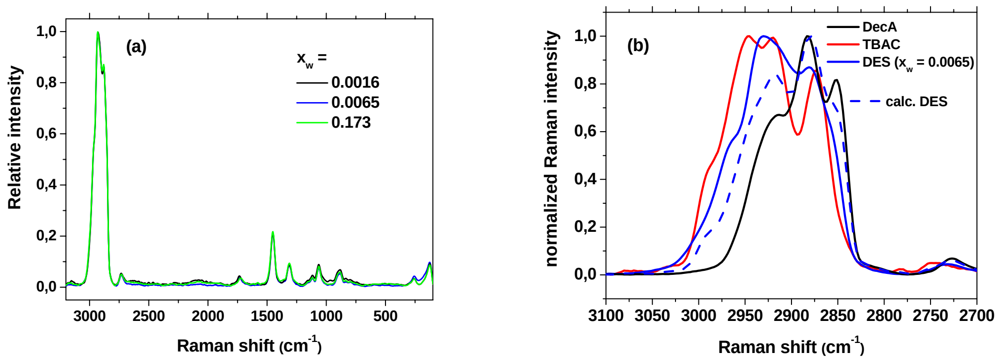

Figure S8. (a), Raman spectra of DESs with different water content; (b), the comparison of the normalized Raman intensity in the C-H region of the DES and its components. Calculated curve is 0.33×TBAC + 0.67×DecA, normalized.

Raman spectra on solid and liquid samples were measured with a Qontor inVia confocal Raman Microscope (Renishaw). The excitation laser wavelength was 532 nm and its power calibration was performed using a Si (111) standard. The maximum power was 50 mW but the laser was set up at 1 % of maximum. Samples were placed on a plastic cap. Water has a negligible effect on the Raman spectra. However, the Raman spectrum of the DES is not exactly the weighted sum of the components, as seen in Fig. S9b. The contribution of TBAC is more pronounced in the DES spectrum with reference to DecA.

Table S1. Assignments of the IR peaks for DES. The component responsible for each peak is shown.

**Wavenumber (cm^{-1})**	**Assignment**	**Component**	**Intensity [a]**
2961	Symm. stretch CH_2	Both	S
2922	Asymm. stretch CH_3	Both	VS
2870	Symm. stretch CH_3	Both	S
2855	Asymm. stretch CH_2	Both	S
1730	Stretch C=O	DecAc	VS
1459	Scissoring CH_3	Both	S
1386	"Umbrella CH_3"	TBAC	M
1334	Wag CH_2	Both	M
1167	Stretching C-N	TBAC	S
1095	Stretching C-O	DecAc	M
1064	Not assigned	/	W
876	Rock CH_2	Both	S
741	Wag C-N	TBAC	M

[a] Intensities of the peaks are reported as VS, S, M, W, which stand for Very Strong, Strong, Medium and Weak, respectively.

Table S2. Assignment of the Raman peaks observed.

**Raman shift in the DES [a] (cm^{-1})**	**Raman shift in DecA [a] (cm^{-1})**	**Raman shift in TBAC [a] (cm^{-1})**	**Assignment**
126 (W)	123 (W)	126 (W)	Lattice vibrations
256 (W)	230 (W)	279 (W)	Bending C-C
885 (W)	895 (W)	895 (W)	Rock CH_2
1062 (W)	1063 (W)	1059 (W)	Stretching C-C
1115 (W)	1125 (W)	1144 (W)	Wag CH_2
1316 (W)	1297 (W)	1327 (W)	Twisting CH_2
1450 (M)	1439 (M)	1485 (M)	Bending CH_2
1730 (W)	1639 (W)	Missing	Stretching C=O
2725 (W)	2726 (W)	2748 (W)	Not assignable
2858 (Sh)	2852 (S)	Missing	Symmetric and asymmetric stretching of CH_2 and CH_3
2880 (S)	2881 (S)	2873 (S)	
2930 (S)	2913 (Sh)	2920 (S)	
2962 (S)	Missing	2946 (S)	
Missing	Missing	2989 (Sh)	

[a] Intensities of the peaks are reported as S, M, W, Sh, which stand for Strong, Medium, Weak and Shoulder, respectively.

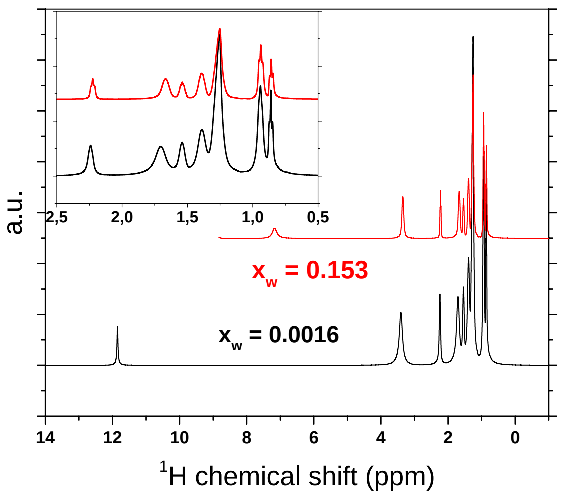

Figure S9. The ^{1}H NMR spectrum of a dry (x_w = 0.0016) and wet (x_w = 0.153) DES at 298K.

The ^{1}H and ^{13}C NMR chemical shifts of the mixture components in a dry and wet DES preparation are given in Table S3, together with their assignment. The ^{14}N NMR spectrum of each preparation consisted of a single broad peak (cf. Fig. S50) due to the TBA nitrogen at −314.5 ppm (dry DES) and −314.7 ppm (wet DES).

Table S3. ^{1}H and ^{13}C NMR chemical shifts δ (ppm) of the TBA and DecA components of dry (x_w = 0.0016) and wet (x_w = 0.153) DES at 298 K. For atom numbering, cf. Scheme 1.

**proton**	**δ in dry DES**	**δ in wet DES**	**carbon**	**δ in dry DES**	**δ in wet DES**
H1’	3.42	3.37	C1’	58.5	58.3
H2’	1.72	1.69	C2’	24.0	23.9
H3’	1.41	1.42	C3’	19.7	19.6
H4’	0.96	0.96	C4’	13.5	13.5
H1	11.87	7.19 [a]	C1	173.8	174.6
H2	2.26	2.25	C2	34.3	34.3
H3	1.56	1.56	C3	25.1	25.0
H4-9	1.27	1.27	C4-7	29.3, 29.4, 29.5, 29.6	29.2, 29.3, 29.5, 29.5
			C8	31.9	31.9
			C9	22.7	22.6
H10	*0.88* [b]	*0.88* [b]	C10	13.9	13.9

[a] Coalesced H1 & H_2O peak. [b] The shift used for chemical shift referencing, see text

4. Water saturation equilibrium

We can define the transfer equilibrium for the transfer of water vapor into DES

$$H_2O(g) \rightleftharpoons H_2O(DES)$$

by (actually, the equilibrium constant here is the inverse of the Henry's law constant)

$$K_W^M = \frac{[H_2O]_{DES}}{p_{H_2O}} \quad \text{or as} \quad K_W^x = \frac{x_{H_2O,DES}}{p_{H_2O}} \; ;$$

the two equations only refer to different standard states. Water concentrations must be corrected to the saturation temperature T by using the volume expansion factors given below.

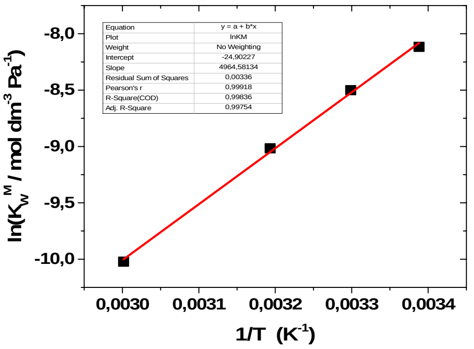

Figure S10. Water saturation of DES as a function of temperature

Because $\Delta_{sat}G^{\circ} = \Delta_{sat}H^{\circ} - T\Delta_{sat}S^{\circ} = -RT\ln K_{sat}$ we have

$$-\Delta_{sat}H^{\circ} \approx -(41 \pm 1)\,\mathrm{kJ\,mol^{-1}}$$

$$\Delta_{sat}S^{\circ} \approx -(207 \pm 4)\,\mathrm{J\,K^{-1}\,mol^{-1}}$$

The saturation process is energetically favorable (exothermic), as can be expected because hydrogen bond formation is usually an exothermic process. On the other hand, it is entropically unfavorable. This is a common case when gases dissolve in liquids, and it causes the gas solubility to decrease with increasing temperature. This factor actually limits the amount of water in DES even though temperature would be raised. Because we assume that at every temperature used the DES is saturated with water vapor the solvents so formed and cooled down to room temperature should, in fact, be oversaturated with water.

5. Differential scanning calorimetry (DSC)

The following sample treatment was applied: 1) equilibration at -55˚C for 10 minutes; 2) heating from -55˚C to 120˚C at 1˚C/min; c) annealing at 120˚C for 10 minutes; d) cooling from 120˚C to -55˚C at 5˚C/min.

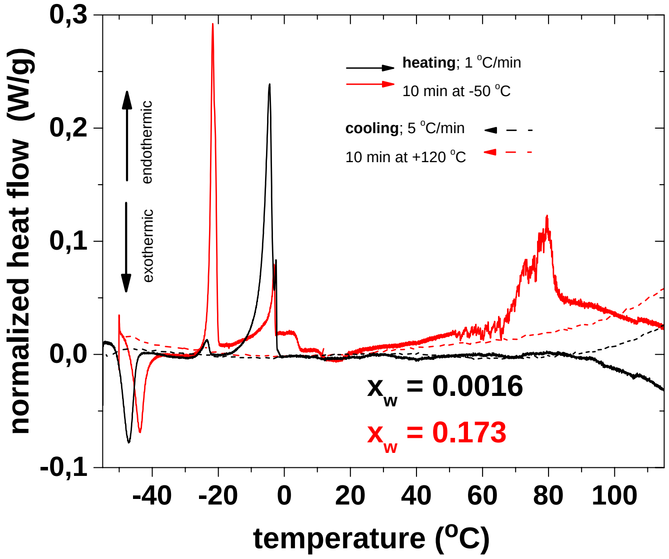

Figure S11. Differential scanning calorimetric scans (heating and cooling) of dry (x_w = 0.0016) and wet (x_w = 0.173) DES samples.

Table S4. Thermal properties of dry and wet DESs [a]

	T_p (°C)<br>[ΔH (J g^{-1})]					
x_w = 0.0016	-47.0<br>[-18.8]	-23.4<br>[+1.6]	-4.5 (-2.5) [b]<br>[+47.6]			
x_w = 0.173	-43.8<br>[-16.4]	-21.7<br>[+32.1]	-3.1<br>[+5.9]	+4.2[c]	+11.1[c]	+65[d]<br>[+40]

[a] T_p = peak temperature upon heating (1 °C / min) after background correction; ΔH, "+" sign exotermic, "-" sign endothermic for enthalpy changes; [b] sharp peak; [c] level change; [d] reaction with the substrate

Van Osch *et al.*, who studied the same DES with the water mole fraction of 0.086, reported a exothermic peak at around -60˚C (that we are not able to see, because of the instrument used), an exothermic peak at around -40˚C (observed in this work, too) and an endothermic peak (attributed to melting) at -11.95˚C.[3] Taking this value into account the temperature of the major endothermic processes depends nearly linearly on the water mole fraction in this range.

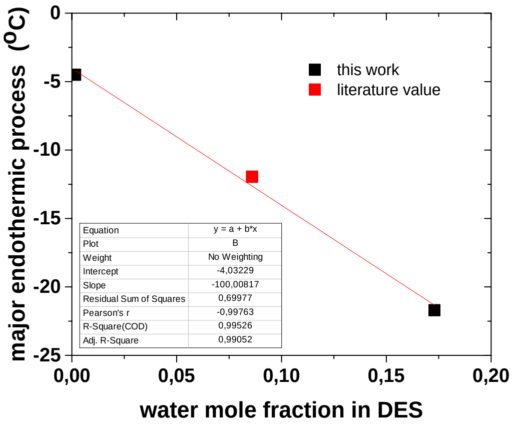

Figure S12. Linear dependence of the melting point (major endothermic peak temperature) of the DES on its water content. Literature value from the work by van Osch *et al*.[3]

6. Molecular modelling

6.A.General

The modelling was applied to TBAC-DecA mixtures (at 1:2 mole ratio) with five different water contents (from dry to close to the observed maximum water content) at different temperatures.

All simulations were performed with GROMACS 2016.1[5–11] using the Bussi-Donadio-Parrinello thermostat (V-rescale)[12]. Simulation pressures were controlled with the Berendsen barostat[13], and with the Parrinello-Rahman barostat[14] during production simulations. Constraints were solved using LINCS[15] with a LINCS order of 4. All production simulations were run with a 1 fs timestep.

Liquid structures were studied by evaluating the radial distribution functions of key (atom) pairs: Water - chloride, central nitrogen of TBA - chloride, and COOH of DecA - chloride. Self-correlations were computed for all components listed before, and for alkyl chains of TBA. Bin volume and density were normalized, and the evaluation was performed on the whole trajectory.

GROMACS compatible molecular topologies and starting structures were obtained from the Automated Topology Builder (ATB)[16]. Corresponding structures are available with the following information: ATB molid 19774 for decanoic acid and 303364 for the tetrabutylammonium-cation. We have provided the parameters for both compounds in the Appendix at the end of the Supporting Information.

OPLS-AA compatible molecular topologies and starting structures were obtained from the LigParGen server.[17–19] Partial atomic charges for the OPLS-AA models were further refined using a Hirshfeld population analysis using the Gaussian09 software and the B3LYP/6-311G basis set.[20] GROMOS54a7 topologies had a compatible Hessian-based analysis and partial charge assignment done within the ATB suite, and further optimization was not deemed necessary.

Simulations, viscosity, density and MSD calculations, RDFs and clustering calculations were performed using GROMACS 5.15 and later migrating to GROMACS 2016.1. No meaningful differences were observed during the version migration. Intramolecular interactions were calculated with harmonic potentials, as demonstrated in the appendix of the SI. Electrostatic interactions were computed with the smooth particle mesh Ewald (PME) method. All systems were **first** minimized using steepest descent scheme, and subsequently equilibrated in a NVT ensemble at 103 K and 303 K, both simulations lasting 500 ps. Final step of the equilibration was performed with a NPT ensemble, lasting 10 ns.

The classical models employed in the computational section of this study are optimized for simulation of organic compounds in the liquid phase, and have been successfully employed in a multitude of studies including similar systems as ours.[19,21]

Initial test simulations were performed with the size of the simulation cell set at 55.884 nm^3, containing 100 decanoic acid and 50 tetrabutylammonium chloride molecules, and either 0, 10, 18, 25, or 33 water molecules, corresponding to water mole fractions in the range 0.0 – 0.18. These

small simulations were performed in order to verify our approach before applying for HPC resources. Later simulations were extended by a tenfold, containing 500 molecules of tetrabutylammonium chloride, 1000 molecules of decanoic acid, and 0-330 molecules of water. These simulations were performed for 30 ns, with the first half excluded as an equilibration period. The atomic radii used : Cl^- : 4.512 Å; R_4-N^+ (TBA^+): 3.32 Å; O(H2): 3.578 Å; O(carbonyl, CO): 3.322 Å; O(H) (carboxylic, OH): 3.445 Å.

Our approach included model validation utilizing two force fields, OPLS-AA and GROMOS54A7, both of which predicted the same behaviour.[22] Furthermore, we observed no change in the measured properties when the system size was increased by a tenfold, suggesting that the simulations have captured a stable state of the system, emerging from the complex interplay of the simplistic potentials used.

Z-density profiles yield qualitative information about the 'microphases' present in our system, by binning the volume of the simulation cell along the z-axis. Each bin has a volume of $0.05 \times x \times y$ Å^3, where 0.05 Å is the length of the bin along the z-axis, x is the length of the x-axis, and y is the length of the y-axis. Dimensions of the cell were (30.9 Å)^2 × 28 Å = 26.735 nm^3.
The resulting graphs clearly indicate how the addition of water drives the system in to a state of 'microphases', where partial density profiles start to locally converge towards zero. The Z densities of two atoms A and B are denoted by Z_A and Z_B, respectively. The "overlap" of atoms i and j from the Z-density calculations is calculated as

$$S_{ij} = \int_{z_1}^{z_2} Z_i Z_j dz \qquad \text{(S5)}$$

where the limits are the z-values of the ends of the simulation cell. This is only a semiquantitative approach because phase segregation can take place in the x-y directions, too. However, the direction of the axis is arbitrary, and a semiquantitative measure of the phase separation can be obtained by comparing the overlap values of the dry DES to those with water added.

The existence of clustered groups was investigated by utilizing a built-in GROMACS analysis tool, which computes distributions of different sized clusters in the simulation cell. The tool is given a set of atoms and a cut-off radius, which is used to define the clusters. In this study the analysis shows (qualitatively) how the ratio of differently sized clusters change as the mole fraction of water is increased. Because the total number of atoms in the clusters equals the number of those atoms in the sample the numbers of clusters observed were normalised by the total number of atoms in the set to obtain the probability distributions of clusters.

The trajectories of five different atoms were followed (O in water, O in C=O, O in COH, N in TBA^+, and Cl) and the simulated self-diffusion coefficients (D_{MD}) were obtained from their mean-square displacements $\left\langle \left|\Delta\mathbf{r}(t)\right|^2 \right\rangle$ by $D_{MD} = \frac{1}{6t}\lim_{t\to\infty}\left\langle \left|\Delta\mathbf{r}(t)\right|^2 \right\rangle$. The results are shown in section SI9.

The small correction due to the finite-size effects under periodic boundary conditions was taken into account in the simulations.[23]

We emphasize that the simulation results should be considered only indicative and semiquantitative if there is no direct experimental evidence. However, the experimental data and simulation results in this work are compatible and support each other.

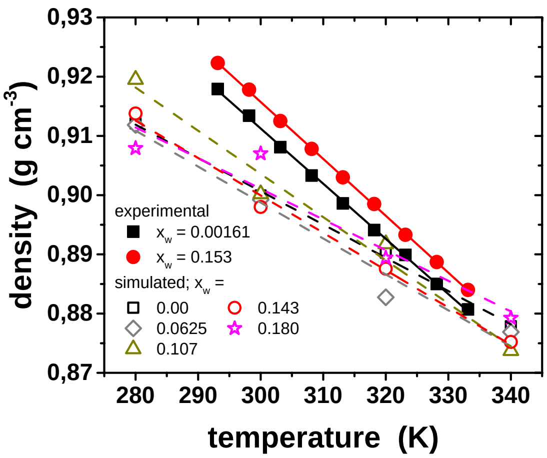

Figure S13. Comparison of experimental and simulated density of DESs with different water fraction as a function of temperature.

6.B. Radial distribution functions

The radial distribution function (RDF) of an atom B around atom A is defined as

$$g_{AB}(r) = \frac{\rho_B(r)}{\langle \rho_B \rangle} \qquad \text{(S6)}$$

where $\rho_B(r)$ is the number density of B at distance r from A, and $\langle \rho_B \rangle$ is the average (bulk) number density of B. The cumulative integral of the RDF allows to calculate the average coordination number of B around A as

$$n_A(B) = 4\pi \langle \rho_B \rangle \int_0^{r_{\min}} g(r) r^2 dr \qquad \text{(S7)}$$

where $r_{\min}$ is the first minimum in RDF after the first peak.

The average bulk atom densities are (the size of the simulation cell was 55.884 nm^3):

$$\langle \rho_{Cl} \rangle = \langle \rho_N \rangle = 8.95 \cdot 10^{-4}\ \text{Å}^{-3} = \langle \rho_{TBA} \rangle$$
$$\langle \rho_{OH} \rangle = 1.79 \cdot 10^{-3}\ \text{Å}^{-3} = \langle \rho_{DecA} \rangle$$

and for water (OW, water oxygen)

$$\langle \rho_{OW} \rangle = 1.79 \cdot 10^{-4}\ \text{Å}^{-3} \text{ when } x_w = 0.0063$$
$$\langle \rho_{OW} \rangle = 3.22 \cdot 10^{-4}\ \text{Å}^{-3} \quad \text{when } x_w = 0.107$$
$$\langle \rho_{OW} \rangle = 4.47 \cdot 10^{-4}\ \text{Å}^{-3} \quad \text{when } x_w = 0.143$$
$$\langle \rho_{OW} \rangle = 5.91 \cdot 10^{-4}\ \text{Å}^{-3} \quad \text{when } x_w = 0.180$$

For HW (water hydrogen) the densities are doubled.

Potentials of mean forces (PMFs) were estimated for the different parts of the DecA carbon chain from the corresponding RDFs using the relation

$$w(r) = RT \ln\left[g(r)\right]$$

where $w(r)$ is the Helmholtz free energy, $g(r)$ the radial distribution function, R the general gas constant, and T the system temperature.

Water self-correlation in DES

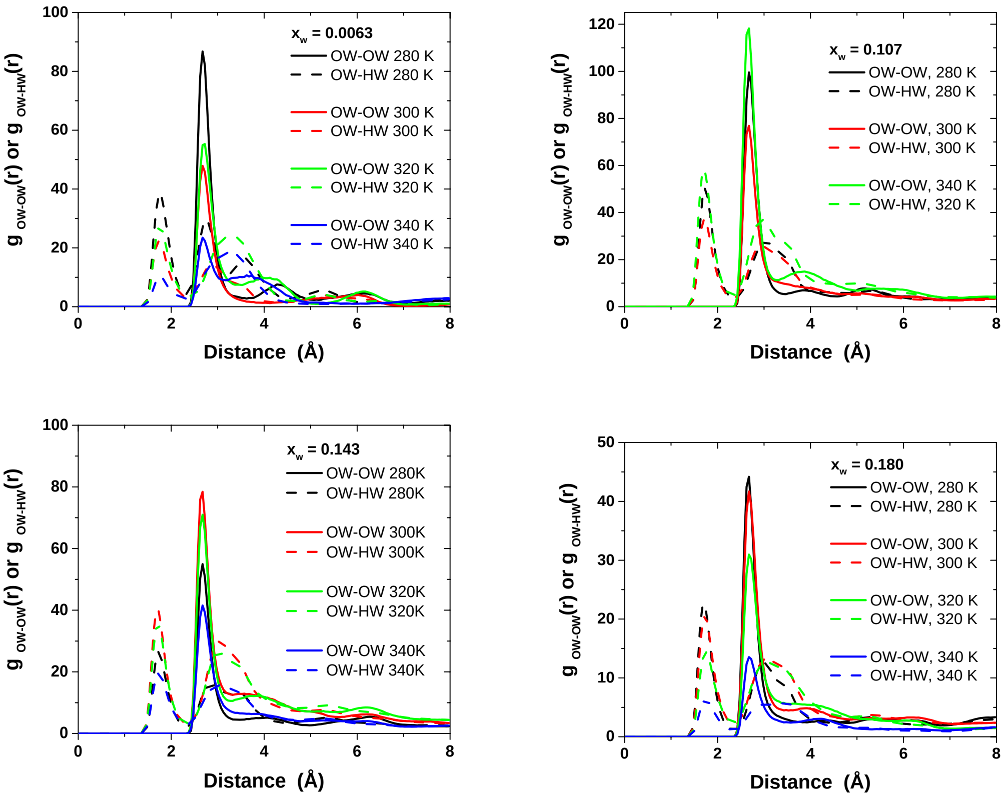

Figure S14. Water self-correlation in DES. Radial distribution functions (RDF) $g_{OW-OW}(r)$ and $g_{OW-HW}(r)$ as a function of temperature at fixed water mole fractions. Cumulative integrals omitted for clarity (see below). OPLS-AA force field.

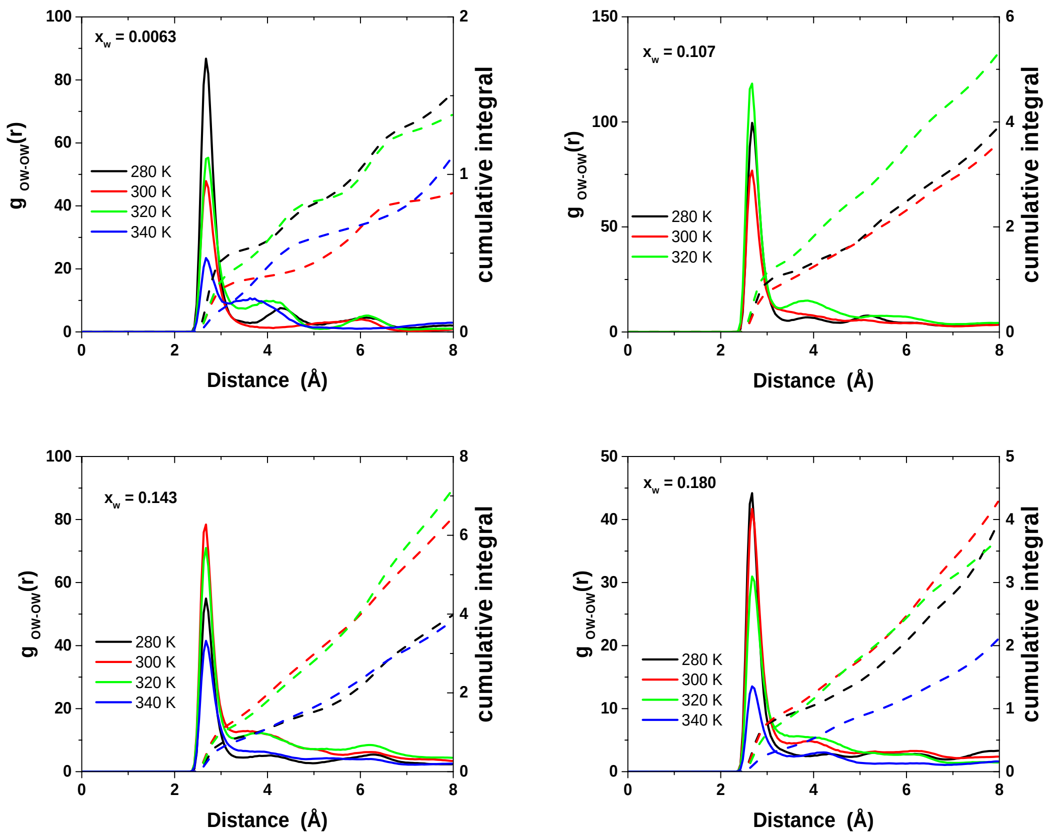

Figure S15. Water self-correlation in DES. Radial distribution function (RDF) $g_{OW-OW}(r)$ and the cumulative integrals as a function of temperature at fixed water mole fractions. OPLS-AA force field.

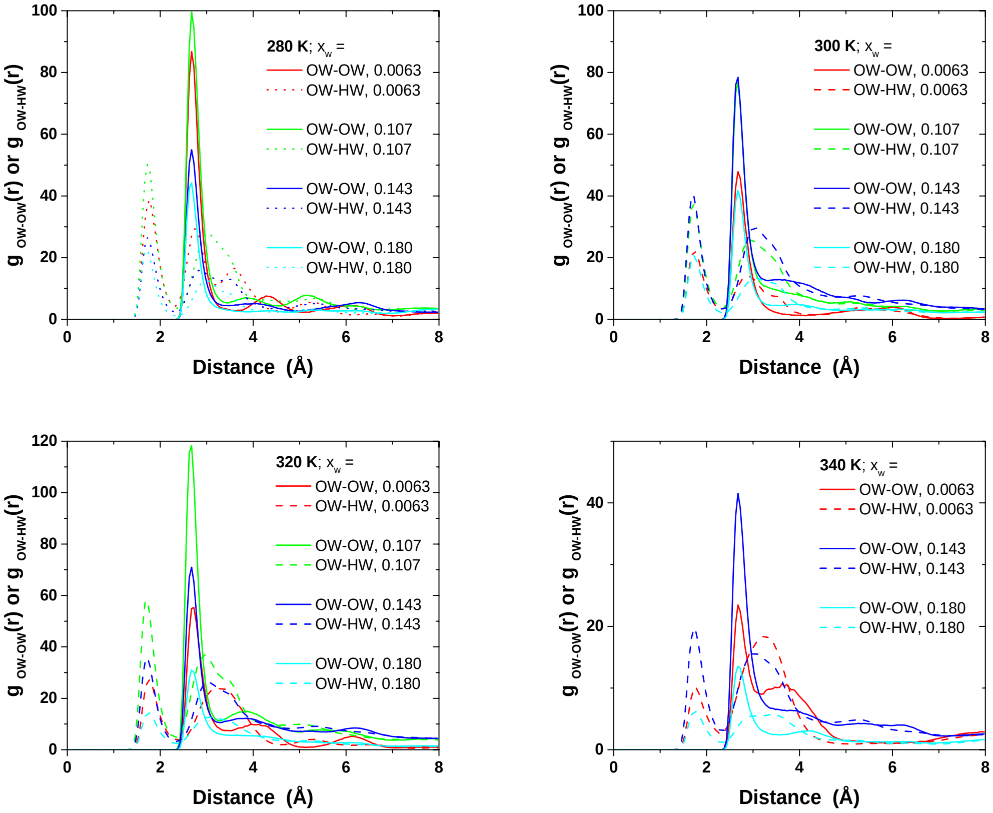

Figure S16. Water self-correlation in DES. Radial distribution functions (RDF) $g_{OW-OW}(r)$ and $g_{OW-HW}(r)$ as a function of water mole fraction at fixed temperature. Cumulative integrals omitted for clarity (see below). OPLS-AA force field.

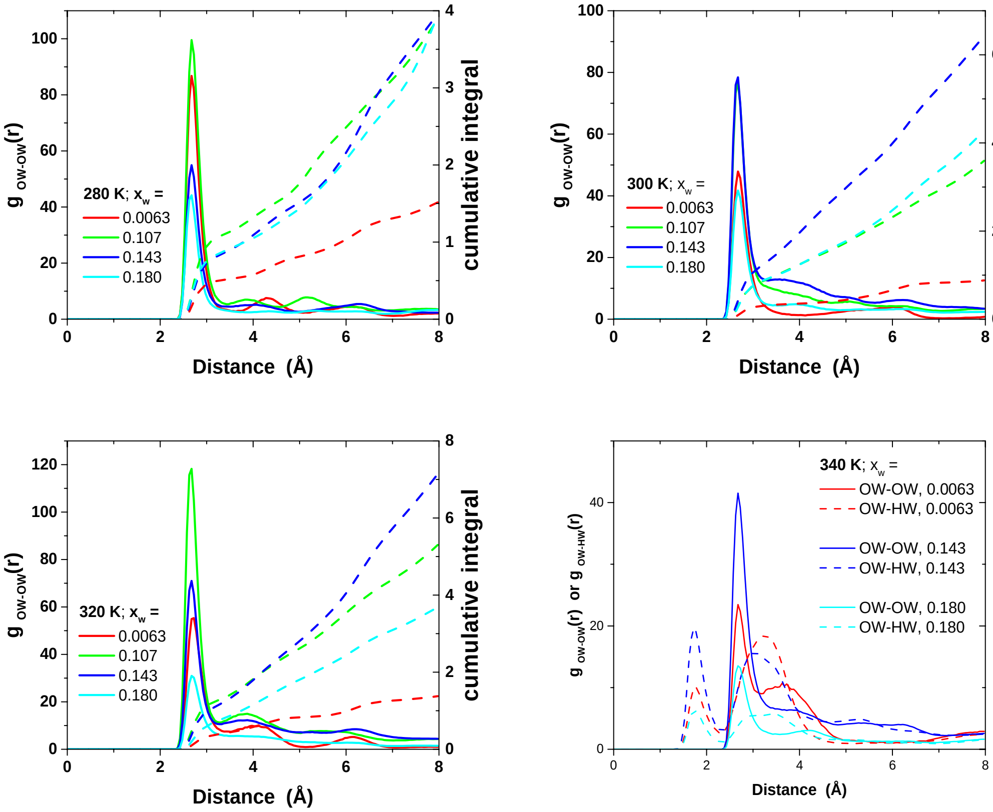

Figure S17. Water self-correlation in DES. Radial distribution function (RDF) $g_{OW-OW}(r)$ and the cumulative integrals as a function of temperature at fixed temperature. OPLS-AA force field.

Conclusions

The OW-OW RDF showed the first coordination sphere peak at 2.7 Å, followed by other peaks with rather complicated behavior. The 2.7 Å peak corresponds to hydrogen-bonded water molecule $O \cdots H - O$.[24] The coordination number (amount of water O in the first solvation sphere) is always lowest at lowest water fraction (x_w = 0.0063) but there seems to be an "optimum" at water fraction 0.1 – 0.15. Temperature broadens the coordination sphere but the behaviour of the coordination number seems rather complicated. In water, the coordination number of OW around OW is approximately 5 but it is much lower (0.2 – 1.3) in DES with different water content and temperature.[24] A low self-coordination number of water (1.5) has been predicted for malicine (1:1 mixture of choline chloride and malic acid) even at water fraction 0.5, and has been interpreted to imply a small degree of water self-clustering.[25]

The OW-HW RDF has a sharp peak at ca. 1.75 Å, which corresponds to the closest hydrogen atom in the hydrogen-bonded water molecule. Another peak at ca. 3 Å is much wider, and corresponds to the other H atom in that water molecule (because the geometry is not locked the distance can vary and produce a wide peak). This peak has a shoulder, which is clearly separated at 280 K in the driest sample (x_w = 0.0063). This suggests two favourable geometries for the closest water molecule. The peak maxima are very close to the values derived from experimental data (1.8 and 3.3 Å),[24] which supports the validity of the method.

Water – Cl correlation in DES

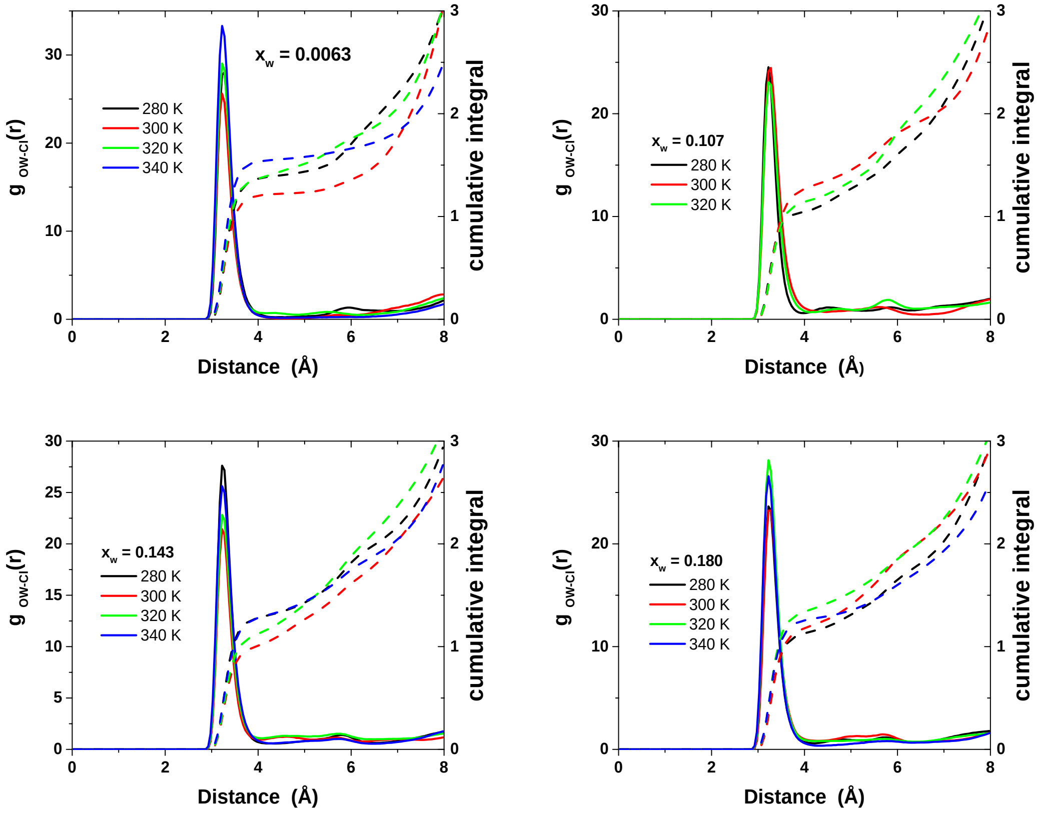

Figure S18. Radial distribution function (RDF) $g_{OW-Cl}(r)$ as a function of temperature at constant water mole fraction. OPLS-AA force field.

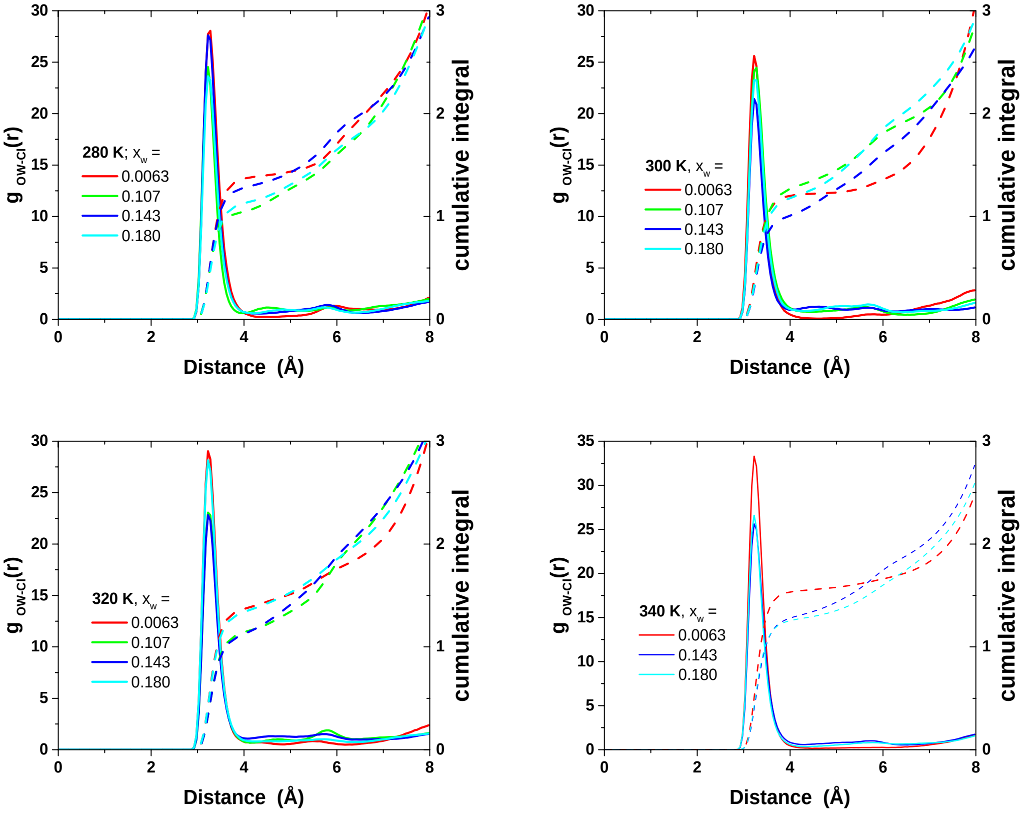

Figure S19. Radial distribution function (RDF) $g_{OW-Cl}(r)$ as a function of water mole fraction at constant temperature. OPLS-AA force field.

The first coordination sphere of Cl around water oxygen (OW) at ca. 3.2 Å contains 1.2 – 1.5 chloride atoms, the position independent of temperature and water content. This is very close to the Cl-O distance reported for aqueous chloride solutions.[26,27] Much smaller broad peaks at ca. 4.5 – 4.9 Å and 5.5 – 5.9 Å. Effect of temperature on coordination number is not clear, the values (below 4 Å) go through a minimum or maximum at different water fractions, being in the range 1.0 – 1.5. When the water fraction in DES is increased from zero to x_w = 0.0063 the coordination number of Cl around OW is 1.2 – 1.5; the same addition of water drops the Cl coordination around OH from 0.9 – 1.0 to 0.45 – 0.6. The coordination of Cl around OW does not significantly change in DESs with higher water content.

Water effectively solvates Cl and "frees" it from OH. As the water concentration is always much smaller than that of OH the hydrogen bonding of Cl to water OH (HW) is favourable to bonding to the carboxylate OH. Because there are more Cl atoms than OW atoms this implies that all water molecules are surrounded by chloride.

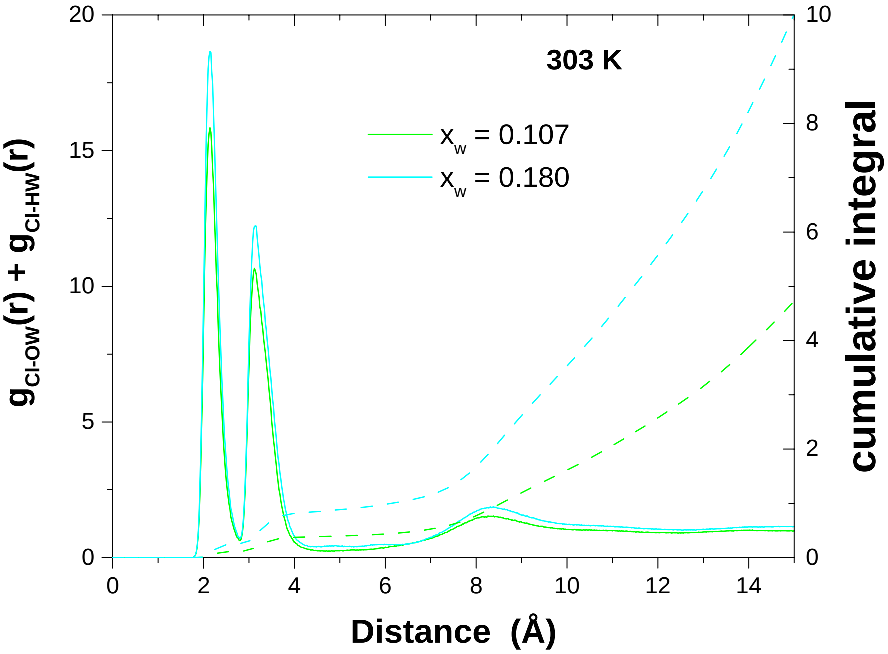

Figure S20. Sum of the radial distribution functions $g_{Cl-OW}(r) + g_{Cl-HW}(r)$ at different water mole fractions (at 303 K). GROMOS force field.

In the first hydration sphere around a chloride ion, the peak at 2.15 Å (cut-off distance 2.80 Å), with coordination number of 0.12 (x_w = 0.107) or 0.39 (x_w = 0.180), is attributed to the closest Cl-**H**OH distance. The second peak at 3.1 Å (cut-off distance 4.70 Å), cumulative coordination numbers 0.26 (x_w = 0.107) or 0.86 (x_w = 0.180), corresponds to the Cl-**O**H_2 distance. Less well-defined coordination sphere at ca. 8.4 Å. The first coordination sphere around Cl^- in aqueous solutions is reported at 3.16 Å[28], or at ca. 3.2 Å[27] (Cl-**O**H_2 distance), with the Cl-**H**OH at ca. 2.25 Å.

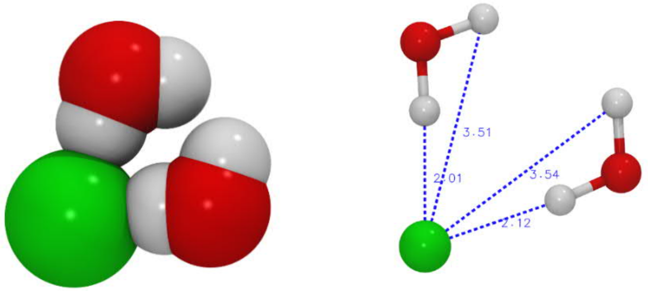

Figure S21. Snapshot of the hydration of the chloride ion. Colors: O, red; H, white; Cl, green.

OH – Cl correlation in DES

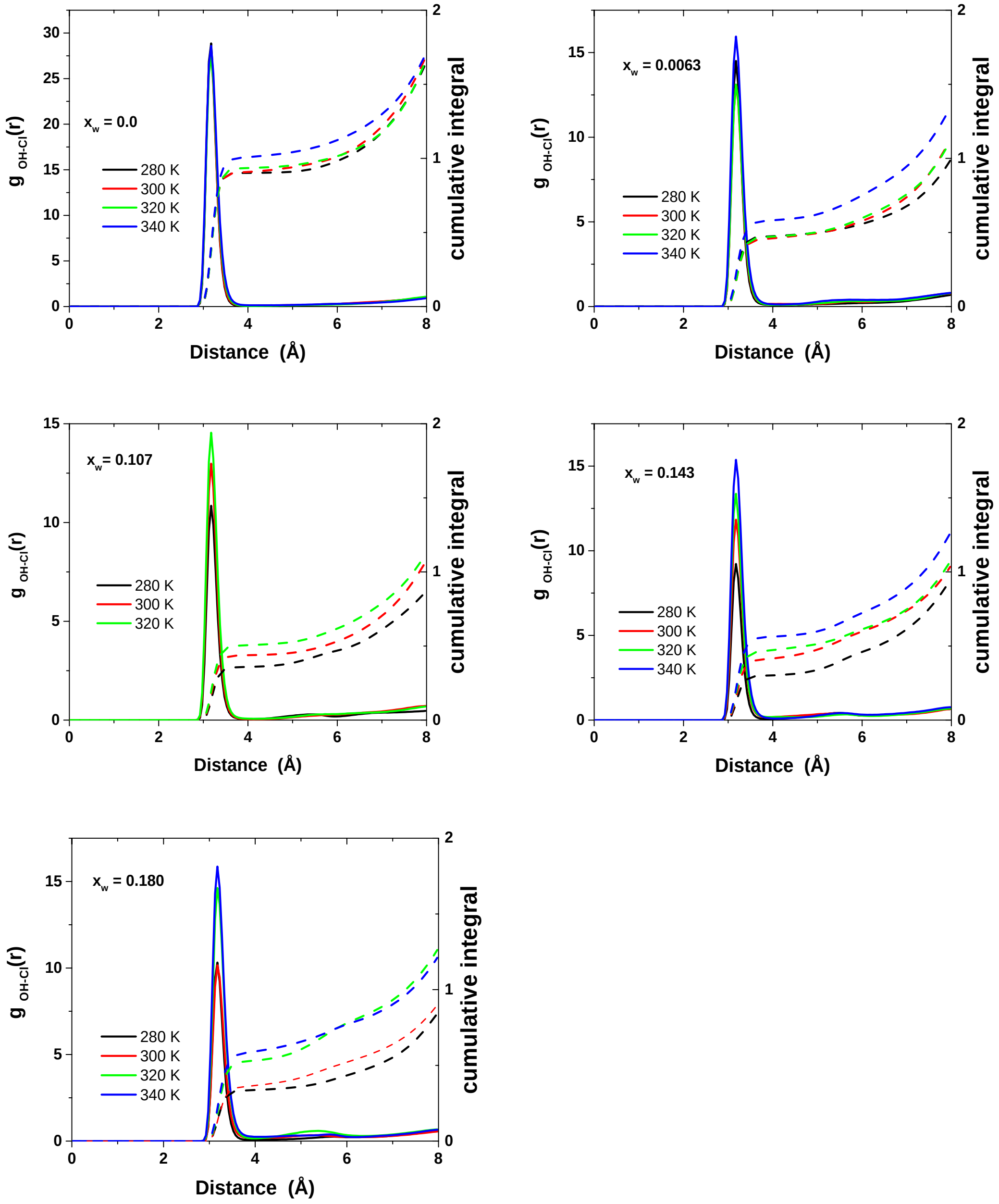

Figure S22. Radial distribution function (RDF) $g_{OH-Cl}(r)$ as a function temperature at fixed water mole fraction. OPLS-AA force field.

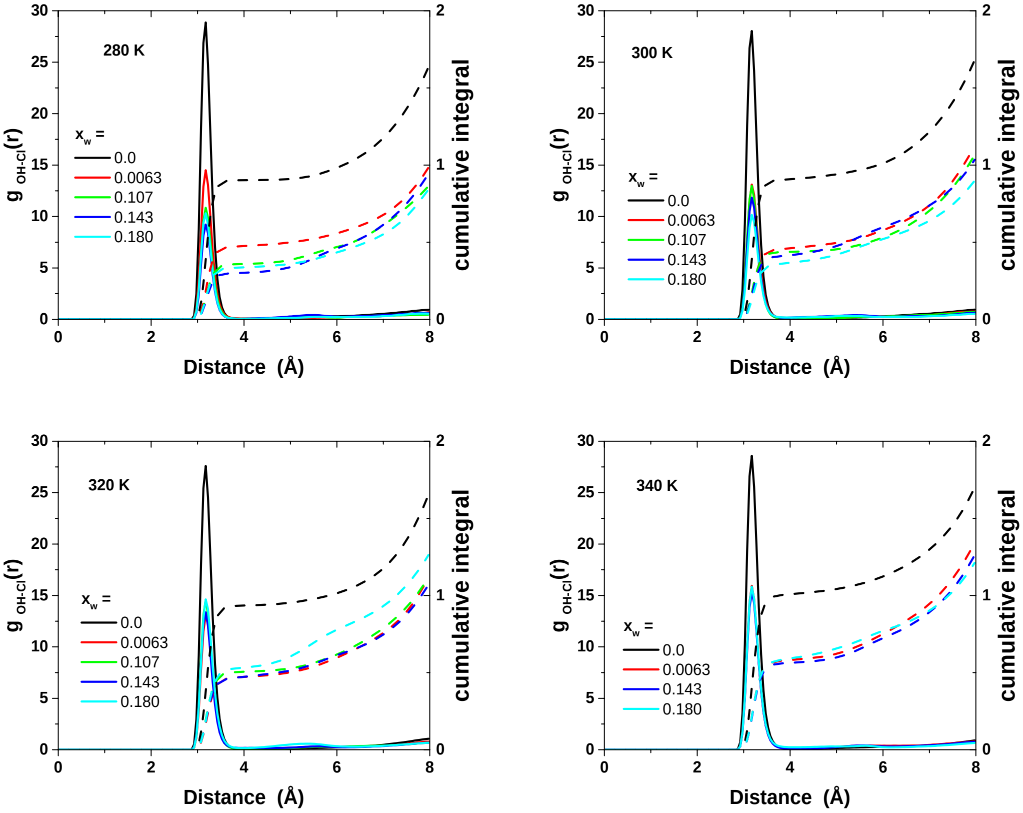

Figure S23. Radial distribution function (RDF) $g_{OH-Cl}(r)$ as a function water mole fraction at constant temperature. OPLS-AA force field.

Conclusions (OH = oxygen in DecA OH group)
One sharp peak at 3.2 Å, very small and broad secondary peak above 5 Å in DES with water (not in dry DES). Coordination number 0.9-1.0 in dry DES, drops to 0.3 – 0.56 in DES with water (the higher the higher the temperature). No significant change with water fraction after the initial introduction of water (when x_w 0.0 → 0.0063), except a small change at 280 K. Temperature may increase the mobility of Cl and allow it to approach OH better at higher temperature.
Water competes with OH for the coordination of Cl, thereby decreasing the coordination number of Cl around OH. The Cl-HO hydrogen bonding is considered the basis of DES formation and, therefore, water effectively disrupts the DES structure.

N – Cl correlation in DES

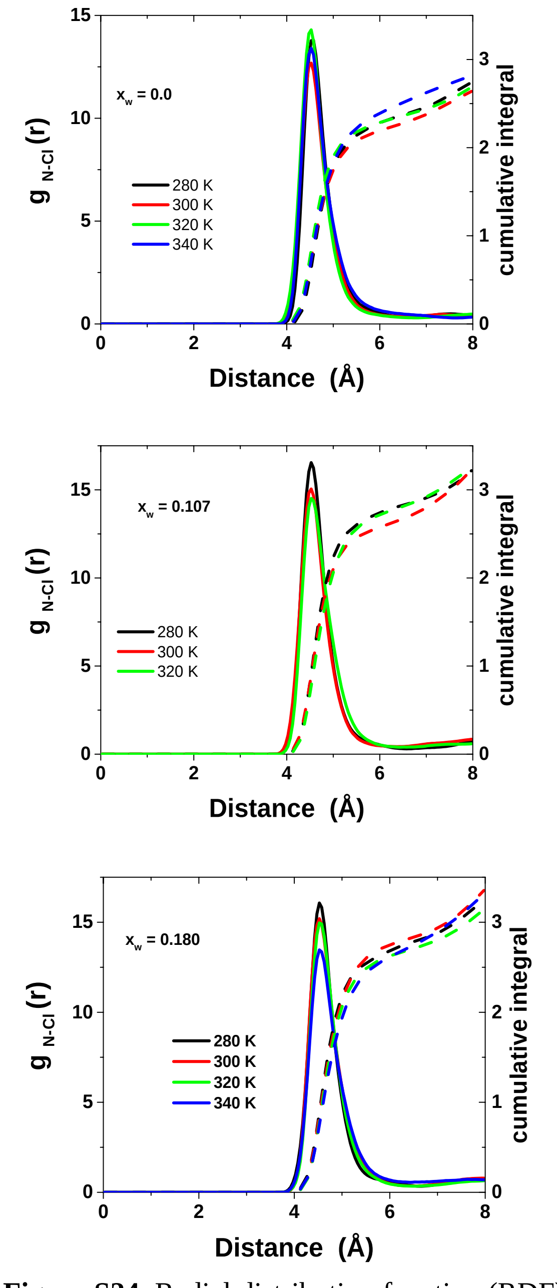

Figure S24. Radial distribution function (RDF) $g_{N\text{–}Cl}(r)$ as a function temperature at fixed water mole fraction. OPLS-AA force field.

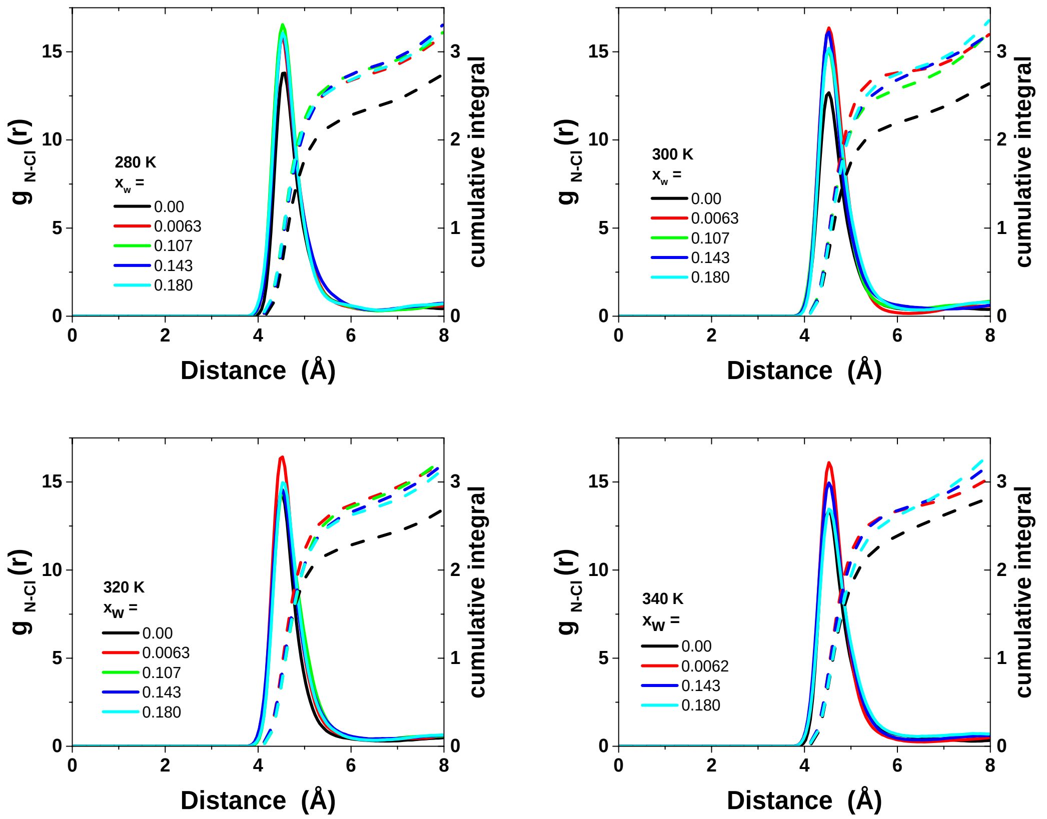

Figure S25. Radial distribution function (RDF) $g_{N-Cl}(r)$ as a function of water mole fraction at constant temperature. OPLS-AA force field.

Conclusions

In all cases there is a single peak at 4.5 Å, and no structure beyond that. This is close to the observed shoulder in the N-Cl RDF for the choline – Cl pair (ca. 5 Å), which has been attributed to chloride ion next to the positively charged quaternary nitrogen, and to the N-Br distance in concentrated aqueous solutions of TBABr (5 Å).[25,29] This suggests that the halide anion penetrates inside the hydrocarbon arms of TBA^+ in DES even more than in the reported cases. The coordination number (Cl around N) is ca. 2.7, except in the dry sample ($x_w = 0.0$), where it is 2.2-2.4. Therefore, addition of a small amount of water increases the average number of Cl atoms (chloride anions) around the N atom (N^+). The temperature has only a negligible effect. In concentrated aqueous TBABr solutions the coordination number of Br around N is only ca. 1/3.[29] The peak at 4.5 Å is rather broad, which implies variable geometry around the nitrogen atom. It is close to the RDF peak 4.7 Å for TEA-BF_4 in acetonitrile.[30] Because of the large size of the TBA^+ ion the N-Cl correlation in DES must represent a contact ion pair with no intervening species, evident also because of the halide penetration inside the hydrocarbon arms of TBA.

Addition of water frees Cl from OH, to which it is hydrogen-bonded in pristine (dry) DES.

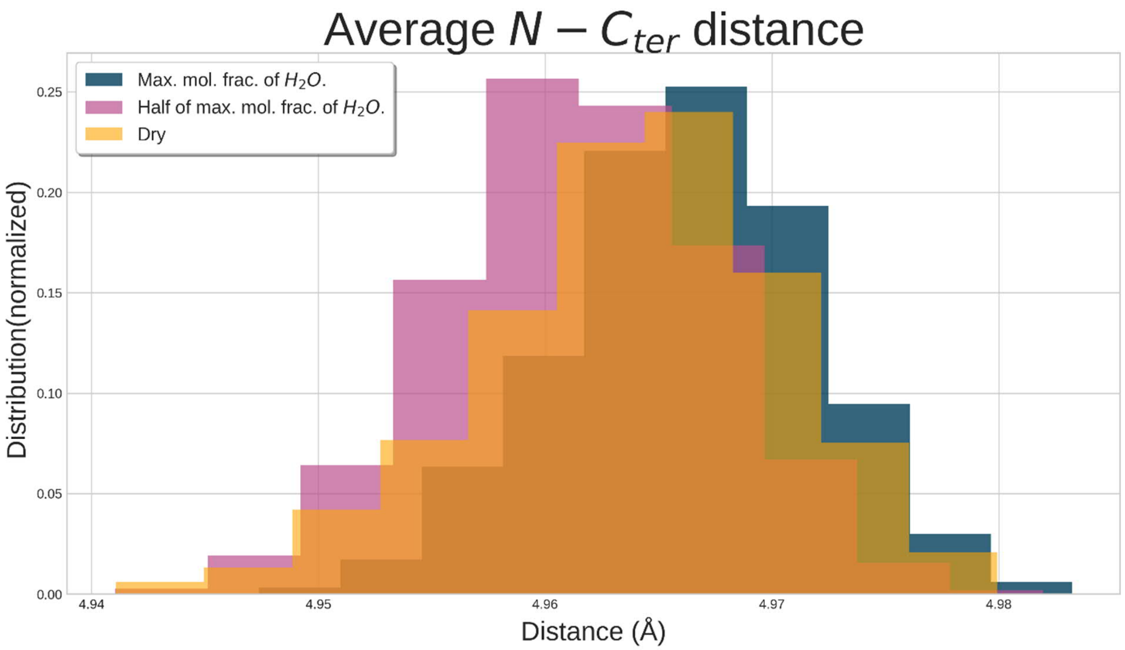

Figure S26. The average distance between the N atom and the C4 atoms of the TBA^+ ion in DESs of different water fractions (maximum water mole fraction $x_w = 0.180$, half-maximum $x_w = 0.107$) based on simulations.

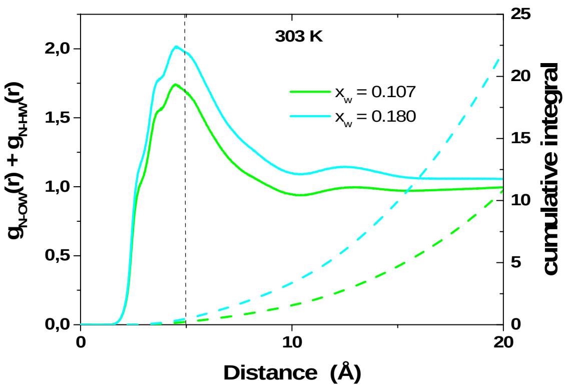

Figure S27. The radial distribution functions $g_{N-OW}(r)+g_{N-HW}(r)$ at 303 K and two different water mole fractions. The hydration sphere around nitrogen is poorly defined, and extends inside the hydrocarbon arms (dashed vertical line is the average N-C4 distance in TBA^+; Fig. S26). Amount of hydration water increases with the water mole fraction in DES. GROMOS force field.

Carboxylic acid COOH self-correlation in DES

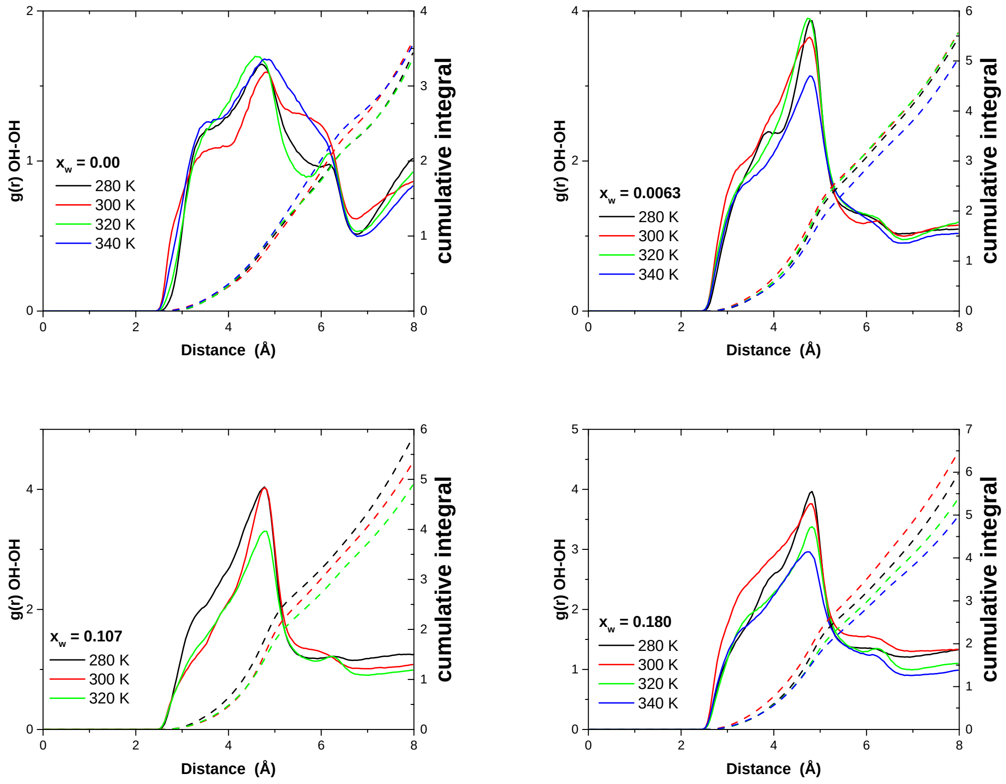

Figure S28. Carboxylic acid self-correlation. Radial distribution function (RDF) $g_{OH-OH}(r)$ as a function temperature at constant water mole fraction. Even a small amounts of water increase the OH-OH self-correlation. OPLS-AA force field.

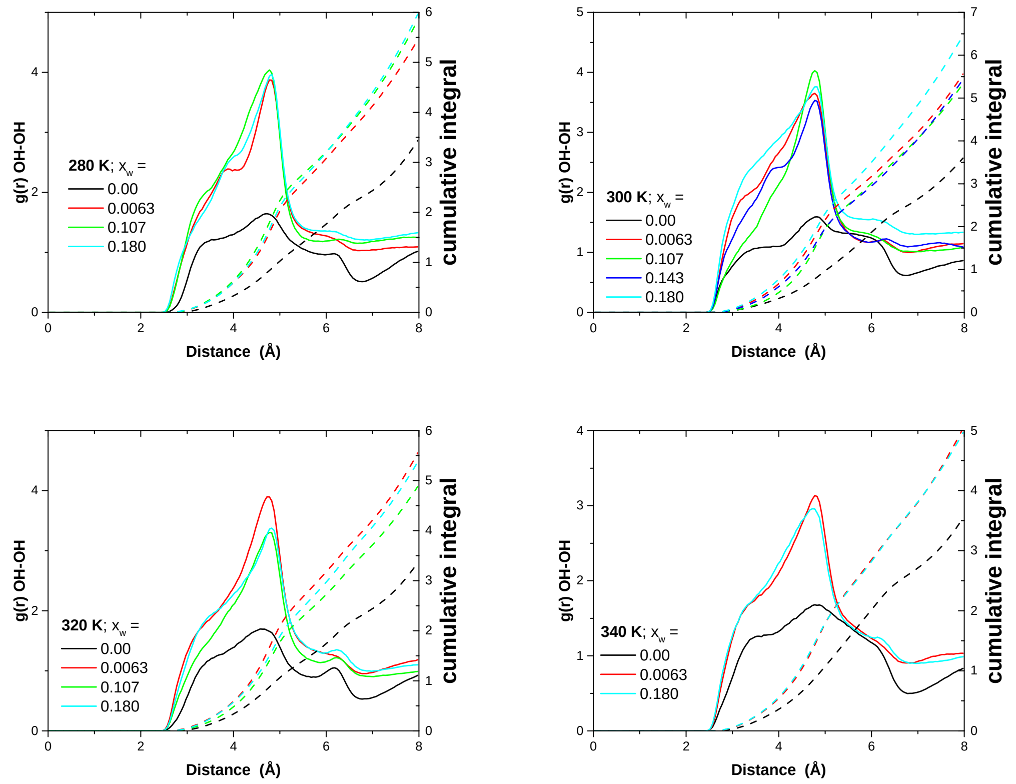

Figure S29. Carboxylic acid self-correlation. Radial distribution function (RDF) $g_{OH-OH}(r)$ as a function water mole fraction temperature at constant temperature. OPLS-AA force field.

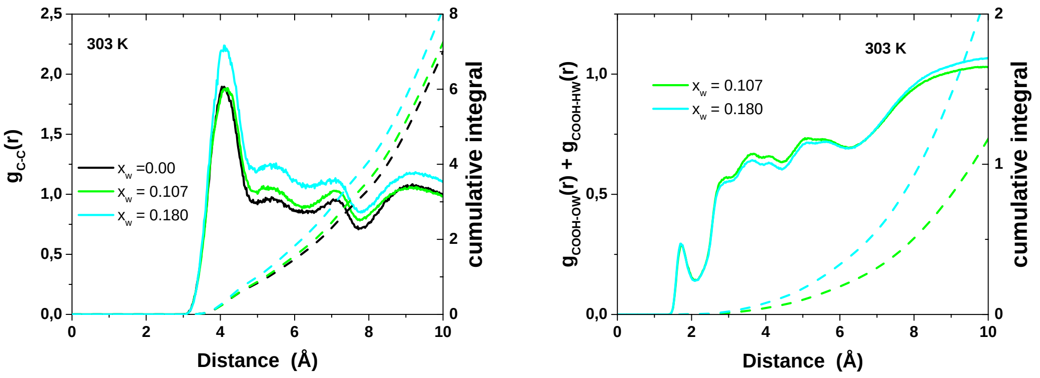

Figure S30. The radial distribution functions $g_{C-C}(r)$ (between the C atoms of the carboxylic acid groups, left) and $g_{C-OW}+g_{C-HW}$ (between the C atom of the carboxylic acid group and O or H atoms of water, right). OPLS-AA force field.

Carboxylic acids form hydrogen-bonded dimers but their structures are considerably less well-defined in solutions than in the gas phase.[31] For acetic acid, in a head-head dimer (COOH-HOOC) the C-C distance is 3.8 Å in the gas phase, and larger (4-4.7 Å) for other dimer conformations. However, in pure or highly concentrated HOAc the maximum in the C-C RDF is at ca. 4.5 Å, which is attributed to a chain of acetic acid molecules (hydrogen bonds C=O --- CH3, C-OH --- O=C). In case of long-chain carboxylic acids, interactions between the chains can modify the dimer geometry in aqueous solutions.[32] The peak at 1.7 Å is the C**O**(OH) – **H**OH (CO – HW) distance. The RDF above resembles the one for acetic acid, except that the peak is at 4.1 Å here. This suggests the presence of other kind of dimers. Less than one DecA around each DecA, except approximately one molecule at high water fraction.

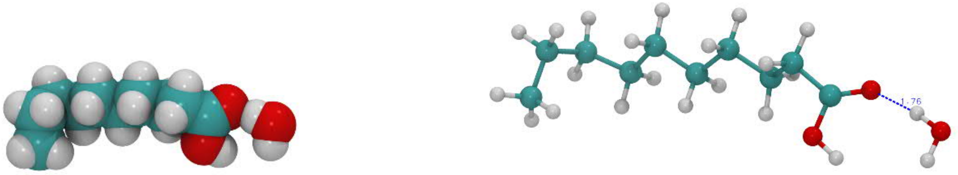

Figure S31. Snapshot of the hydration of the carboxylic acid group. Colors: C, blue; O, red; H, white. The blue dotted distance between HO-H and O=C is 1.76 Å.

DecA carbon chain self correlations and the potentials of mean force (PMFs)

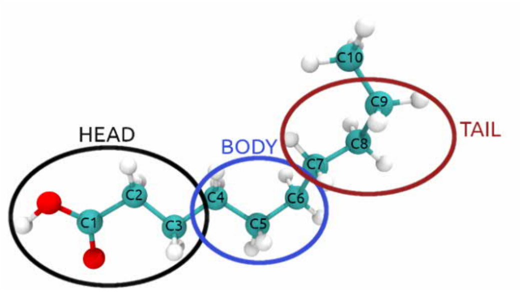

Figure S32. Division of the decanoic acid (DecA) carbon chain into the head (C1-C3), body (C4-C6), and tail (C7-C9) sections.

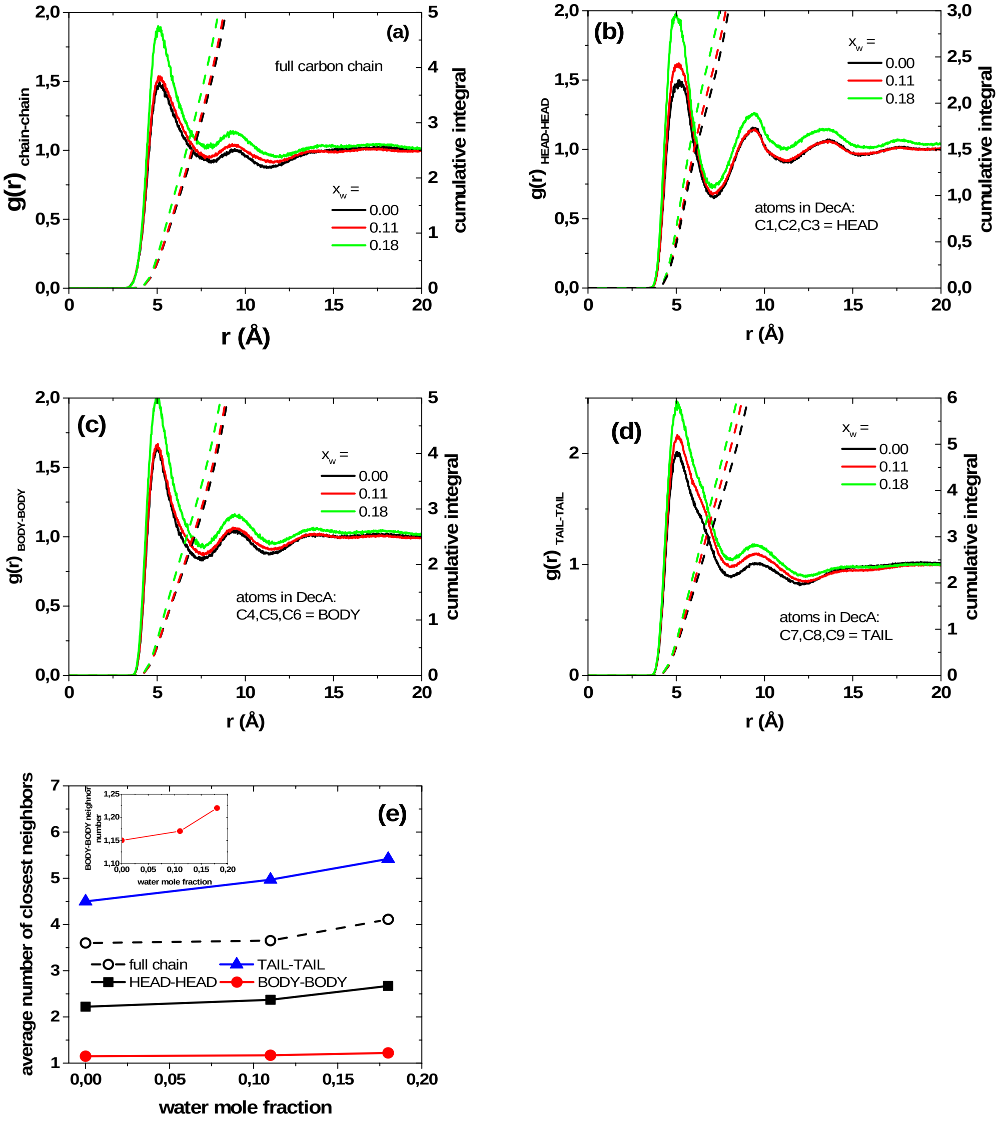

Figure S33. Radial distribution functions (solid lines) and their cumulative integrals (dashed lines) for the mass centers of (a), the DecA carbon chain; (b) the head part; (c), the body part; and (d), the tail part as a function of water mole fraction in the 1:2 TBAC:DecA DES. The first maximum is always at (5.1 ± 0.1) Å, the first minimum at 7.1 – 8.1 Å, the second maximum at 9.4-9.5 Å. The average number of closest neighbors for different parts of the chain is shown in (e).

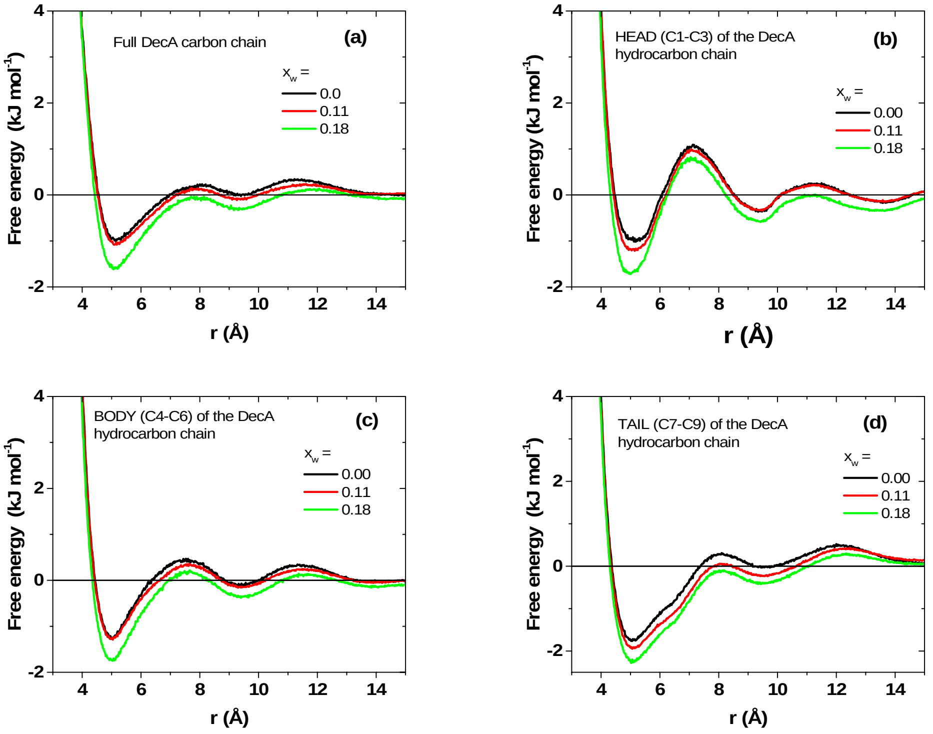

Figure S34. Potentials of mean force (free energies) of interactions between (a), the whole decanoic acid carbon chains; (b), the head parts; (c), the body parts; and (d) the tail parts at different water mole fractions in the 1:2 TBAC:DecA DES. Calculated from the corresponding RDFs using eq. S7.

7. Thermal expansion, excess quantities, and surface tension

Measurements of density and viscosity at different temperatures have been conducted on dry and water-containing DES samples (water-% = 0.014 % w/w, $x_{H_2O} = 0.0016$, and water-% = 1.546 % w/w, $x_{H_2O} = 0.153$, respectively) .

Table S5. Volume expansion factors of DES samples used to normalize the conductivity.

**x_w = 0.00161** Temperature (˚C)	Density (g/cm^3)	volume expansion vs 25 ˚C	**x_w = 0.153** Temperature (˚C)	Density (g/cm^3)	volume expansion vs 25 ˚C
20	0.9179		20	0.9223	
25	0.9134	1	25	0.9178	1
30	0.9081	1.0058	30	0.9125	1.0058
35	0.9033		35	0.9078	
40	0.8986	1.0165	40	0.9030	1.0164
45	0.8941		45	0.8985	
50	0.8899	1.0264	50	0.8933	1.0274
55	0.8850		55	0.8887	
60	0.8807	1.0371	60	0.8840	1.0382

The **thermal expansion coefficient** is given by

$$\alpha = \frac{1}{V}\left(\frac{\partial V}{\partial T}\right)_p = \left(\frac{\partial \ln V}{\partial T}\right)_p \overset{V=m/\rho}{=} \left(\frac{\partial[\ln m - \ln \rho]}{\partial T}\right)_p = -\left(\frac{\partial \ln \rho}{\partial T}\right)_p$$

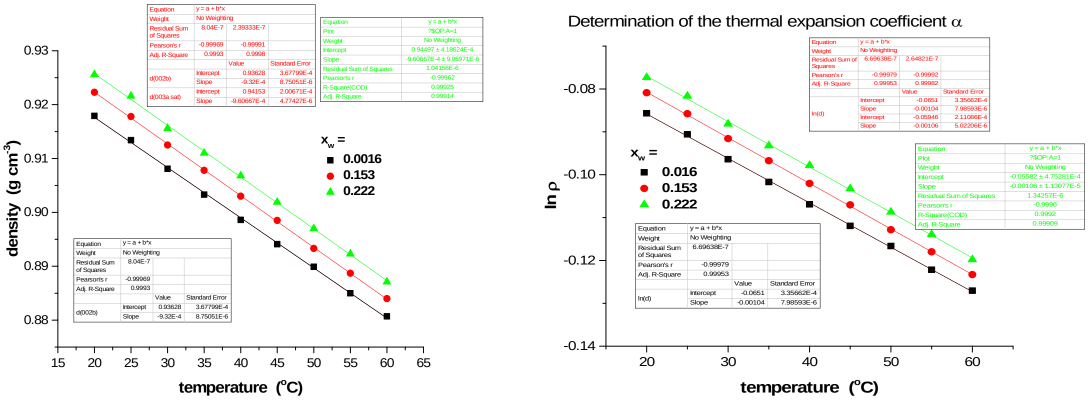

Figure S35. Variation of the density (ρ , left) and its logarithm ($\ln \rho$, right) with temperature of DESs with different water content. The thermal expansion coefficients from the plot are $(1.04 \pm 0.01)\cdot 10^{-3}$, $(1.04 \pm 0.01)\cdot 10^{-3}$, and $(1.06 \pm 0.01)\cdot 10^{-3}$ K^{-1} for water mole fractions x_w = 0.0016, 0.153, and 0.222, respectively.

Thermal expansion coefficients are practically identical; they differ by ca 2 %, i.e., about the difference in water weight- % units.

water-% = 0.014 % w/w, $x_{H_2O} = 0.00161$ $\alpha = (1.040 \pm 0.008) \cdot 10^{-3}\,\mathrm{K}^{-1}$

water-% = 1.546 % w/w, $x_{H_2O} = 0.153$ $\alpha = (1.060 \pm 0.005) \cdot 10^{-3}\,\mathrm{K}^{-1}$

Excess molar volume V^E and excess viscosity η^E; surface tension

The excess quantities are calculated from equations

$$V^E = \frac{x_{DES}M_{DES} + x_x M_w}{\rho_{obs}} - \frac{x_{DES}M_{DES}}{\rho_{DES}} - \frac{x_x M_w}{\rho_w} \qquad \text{(S8)}$$

$$\eta^E = \eta_{obs} - x_{DES}\eta_{DES} - x_w \eta_w \qquad \text{(S9)}$$

The density and viscosity of a pure dry DES have been estimated from the intercept of the linear fits of the corresponding data in Figs. 5a,b, and the effective DES molar mass is taken to be

$$M_{DES} = M_{TBAC} + 2M_{DecA} \qquad \text{(S10)}$$

(the molar mass of a DES is not an unambiguous concept, and some authors have used mole fraction weighed value or the geometrical mean; however, here we regard dry DES as a compound and the wet DES as a mixture TBAC(DecA)$_2$-water).[33]

The scatter in the data for density, viscosity, and conductivity vs. water mole fraction is attributed mostly to the uncertainty of the Karl Fischer titration of small amounts of water.

The surface tension of the DES formed by the 1:2 ratio of TBAC and DecA is (0.0308 ± 0.0009) Nm^{-1} at room temperature, and it is independent of the water content (Fig. S34). This impliesapproximately zero surface excess but, because of the measurement technique, we cannot rule out the possibility of rapid water adsorption on the surface of a dry sessile DES drop.

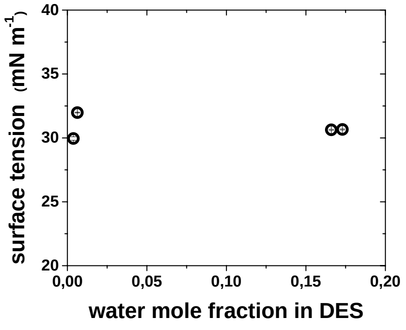

Figure S36. Surface tension of DES, measured using the sessile drop technique, as a function of the water mole fraction.

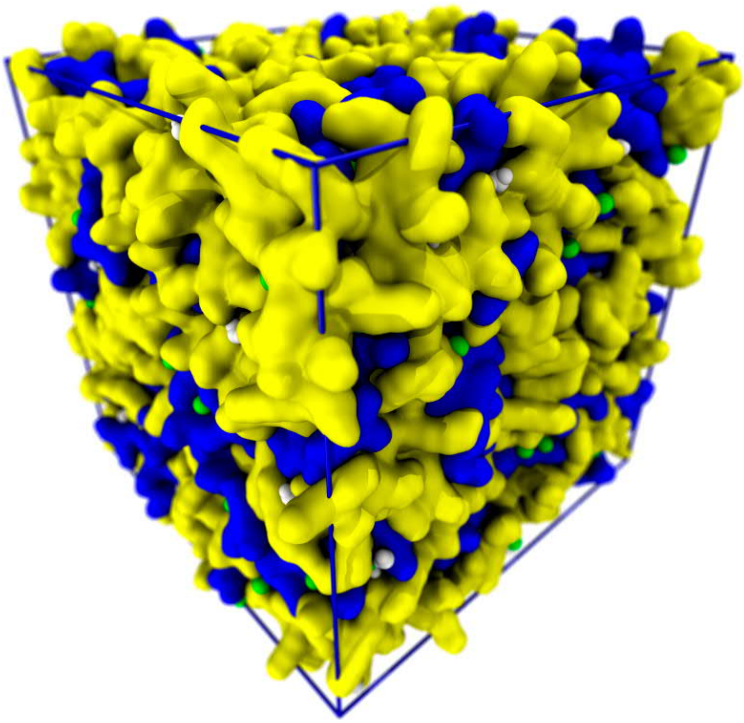

Figure S37. Snapshot of the structure of DES with $x_w = 0.180$ (cf. Fig. 4a). Yellow areas contain DecA, blue areas TBA, chloride and water shown by green and white balls, respectively.

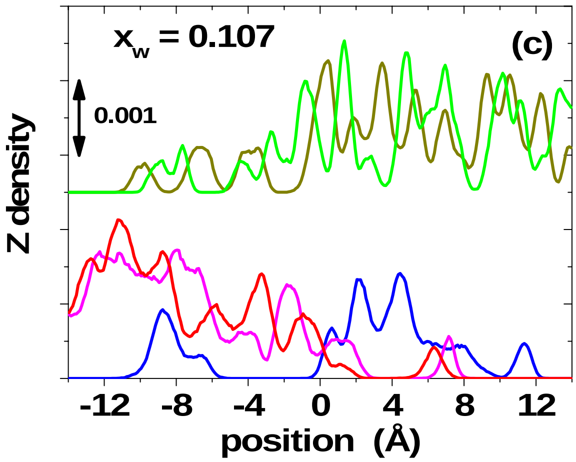

Figure S38. Z-density profiles of the components of DES (TBAC:DecA 1:2) at water mole fraction $x_w = 0.107$ (*cf*. Fig. 3).

8. Viscosity and fragility[34,35]

The Vogel-Fulcher-Tammann (VFT) fits of the temperature dependence of viscosity can be used to obtain fragility parameters for the DES samples. The VFT equation for viscosity is

$$\eta = \eta_0 \exp\left[\frac{B}{T - T_0}\right] \equiv \eta_0 \exp\left[\frac{DT_0}{T - T_0}\right]$$

where D is the Angell fragility parameter.[36] We can also write the VFT equation as

$$\langle\tau\rangle = \tau_0 \exp\left[\frac{B}{T - T_0}\right] \equiv \tau_0 \exp\left[\frac{DT_0}{T - T_0}\right]$$

because $\eta \sim \langle\tau\rangle$, where $\langle\tau\rangle$ = average relaxation time for structural and orientational rearrangements in liquid.

Fragility (also called the steepness index) m is defined using $\langle\tau\rangle$ (or η) as (T_g = the glass transition temperature)[34,35]

$$m = \left[\frac{d\lg\langle\tau\rangle}{d\left(T_g/T\right)}\right]_{T=T_g} \quad \text{where } \lg \equiv \log_{10} \tag{S10}$$

From the VFT equation we find that

$$m = \frac{D}{\ln 10}\frac{T_0}{T_g}\frac{1}{\left(1 - T_0/T_g\right)^2} \tag{S11}$$

It is customary to take T_g to correspond the temperature at which $\langle\tau\rangle_{T=T_g} \sim 100\text{ s}$. The VFT equation yields

$$\lg\langle\tau\rangle = \lg\tau_0 + \frac{D}{\ln 10}\frac{T_0}{T - T_0}$$

Now

$$\lg\langle\tau\rangle_{T=T_g} = \lg\tau_0 + \frac{D}{\ln 10}\frac{T_0}{T_g - T_0} \ (\sim 2)$$

On the other hand, when $T \to \infty$ we have $\lg\langle\tau\rangle = \lg\tau_0$

Physically the minimum fragility (see Fig. 1 in ref. [35]) corresponds to the case when the Arrhenius' law can be used $\langle\tau\rangle = \tau_A \exp\left[\frac{E_{act}}{RT}\right]$

Here E_{act} =apparent activation energy for relaxation (and viscous flow), and the pre-exponential factor has been denoted by τ_A in order to distinguish it from the corresponding term in the VFT equation. Applying the definition of fragility to the Arrhenius' law yields

$$m = \frac{E_{act}}{RT_g \ln 10} = -\lg\tau_A + \lg\left\{\langle\tau\rangle_{T=T_g}\right\} = m_{\min}$$

The last term $\lg\left\{\left\langle \tau \right\rangle_{T=T_g}\right\} = 2$, and experimentally, in systems following the Arrhenius' law, $\log_{10}\tau_A \sim -14$. This represents the minimum of fragility, and correspondingly, $m_{\min} \sim 16$. The Arrhenius's law is obtained from the VFT equation by letting $T_0 \to 0$, and we may equate $\lim_{T\to\infty} \lg\left\langle \tau \right\rangle = \lg \tau_A \sim \lg \tau_0$

From the equation above we have then

$$m_{\min} = \lg\left\langle \tau \right\rangle_{T=T_g} - \lg \tau_0 = \frac{D}{\ln 10}\frac{T_0}{T_g - T_0}$$

Now

$$m = \frac{D}{\ln 10}\frac{T_0}{T_g}\frac{1}{\left(1 - T_0/T_g\right)^2} = \frac{D}{\ln 10}\frac{T_0 T_g - T_0^{\,2} + T_0^{\,2}}{\left(T_g - T_0\right)^2} = \frac{D}{\ln 10}\left[\frac{T_0}{\left(T_g - T_0\right)} + \frac{T_0^{\,2}}{\left(T_g - T_0\right)^2}\right] = \qquad \text{(S12)}$$

$$= m_{\min} + \frac{\ln 10}{D} m_{\min}^{\,2}$$

which is the equation (4b) in ref. [35]. Introducing the numerical values we have

$$m \approx 16 + 590/D \qquad \text{(S13)}$$

which has been used in this work to estimate the fragility.

In glass-forming liquids, the following expression has been put forward to estimate the glass transition temperature from fragility parameters,[34] and this estimate is shown in the last column of the Table S5.

$$\frac{m}{m - 16} = \frac{T_g}{T_0} \qquad \text{(S14)}$$

Table S6. Vogel-Fulcher-Tammann parameters for viscosity data. Estimated fragility m, Agnell fragility parameter$D$, and glass transition temperatureT_g.

**water mole fraction in DES**	$\ln\eta_0$	***B* / K**	**T_0 / K**	***D***	***m***	**estimated T_g / K**
0.0016	-9.7 ± 0.2	1180 ± 50	165 ± 3	7.2 ± 0.5	98 ± 7	ca. 200
0.153	-9.6 ± 0.2	980 ± 40	171 ± 3	5.8 ± 0.4	118 ± 8	ca. 200
0.222	-12 ± 1	1850 ± 500	110 ± 30	17 ± 9	51 ± 30	ca. 165

9. Self-diffusion coefficients

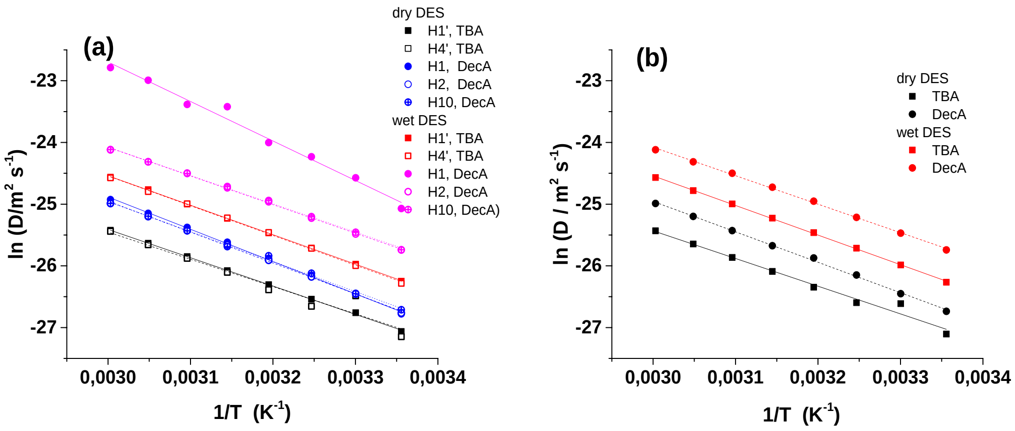

Figure S39. Self-diffusion coefficients measured using (a), the 1' and 4' protons in TBA, and 1 (+H_2O), 2, and 10 protons in DecA as a function of temperature, and (b), the average of protons 1' and 4' in TBA and protons 2 and 10 in DecA as a function of temperature. Lines are the Arrhenius' law fits to data. Water mole fractions 0.0016 and 0.153 in the dry and wet DES sample, respectively.

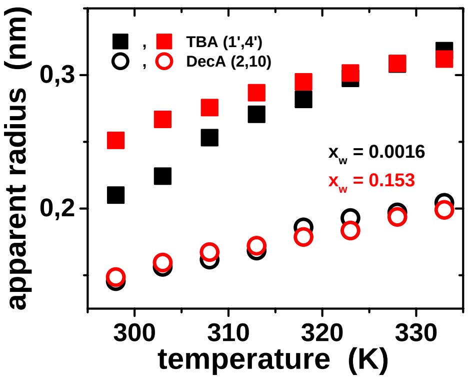

Figure S40. The apparent hydrodynamic radii of TBA and DecA as calculated based on the Stokes-Einstein equation $D = kT/C\eta R$, with $C = 6\pi$ (no-slip boundary conditions) and the experimental diffusion coefficients (from NMR). A change in the physical boundary condition with temperature from slip to no-slip is is improbable, and the values are well below the hard sphere radii.

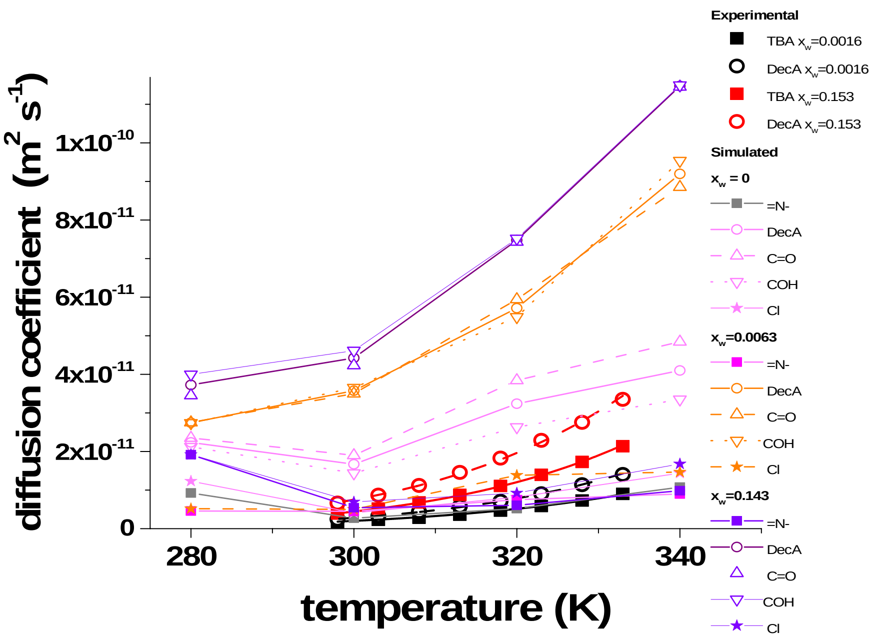

Figure S41. Comparison of the simulated and observed (by NMR) self-diffusion coefficients.

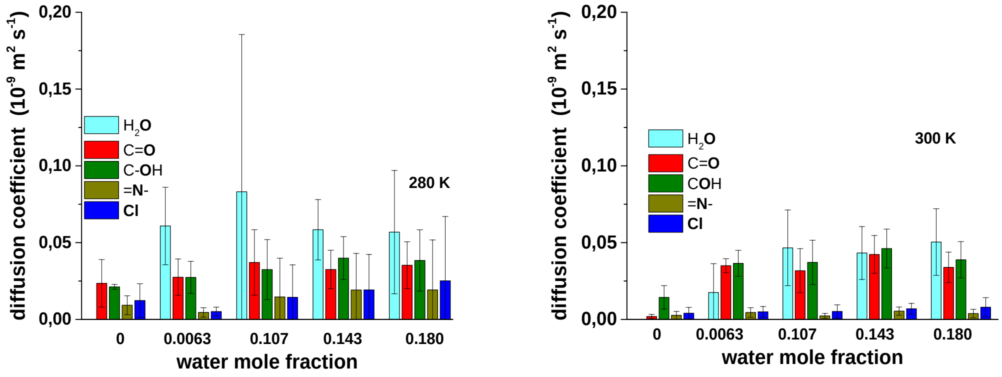

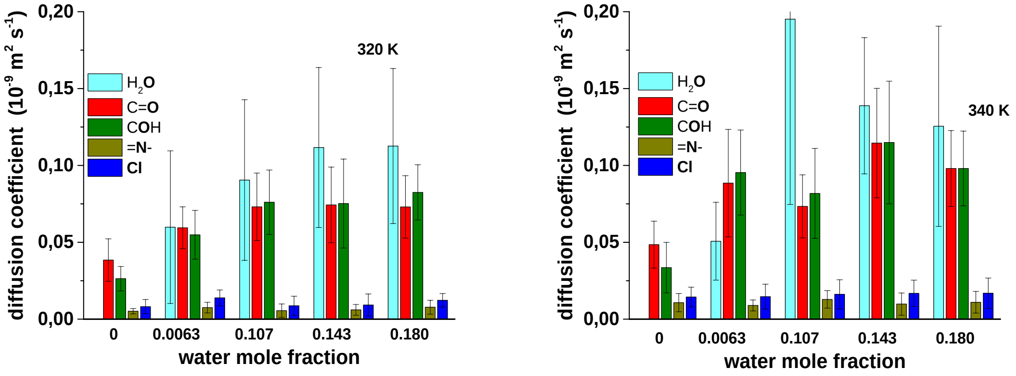

Figure S42. Self-diffusion coefficients for different species (atoms followed in **bold**) from simulations, at different temperature and water mole fraction.

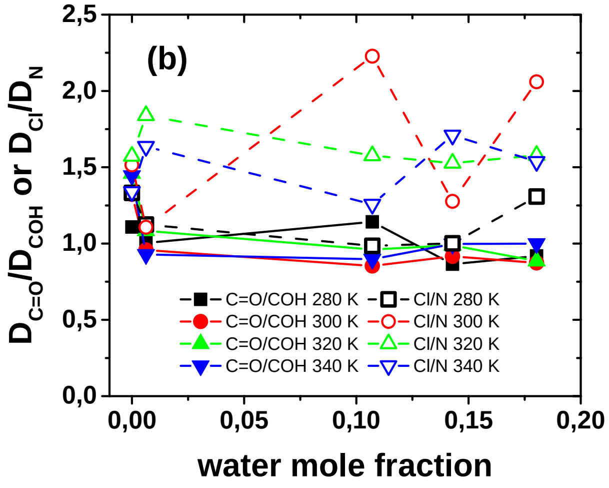

Figure S43. The ratio of the simulated diffusion constants for the C=O and C-OH oxygens (solid lines and symbols) and the chloride ions and quaternary N (dashed lines, open symbols) at different temperatures as a function of water mole fraction in DES. Lines shown only as a guide to the eye. The C=O/COH pair serves as a reference data as both of the groups are in the same molecule.

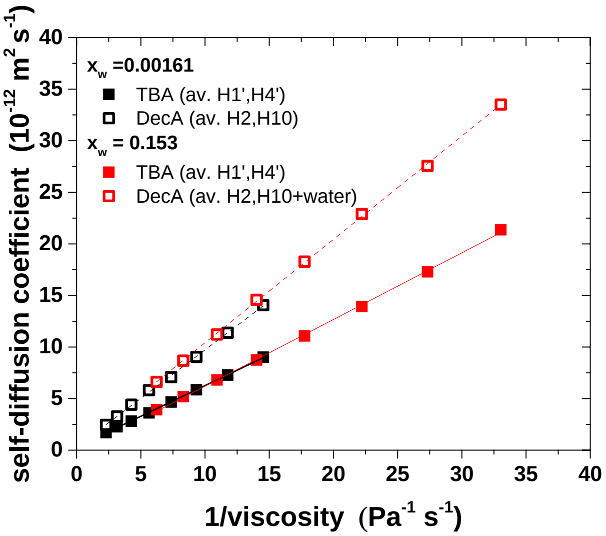

Figure S44. Self-diffusion coefficients of TBA and DecA (averaged for the hydrogens shown) as a function of inverse viscosity in DESs with two different water mole fraction (lines are linear fits to data).

10. Conductivity

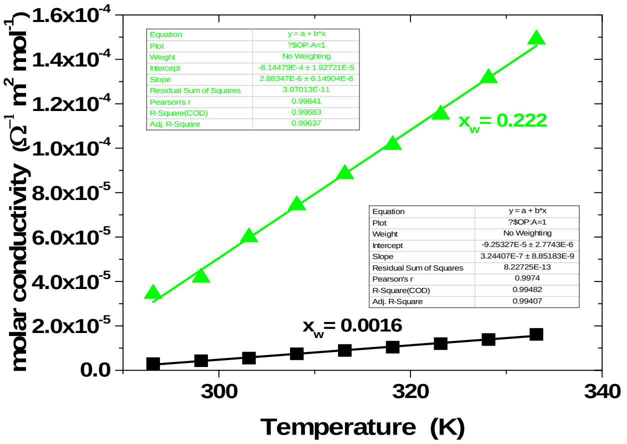

Figure S45. Molar conductivity of DESs (corrected for thermal expansion) with different water content as a function of temperature. Water content $x_w = 0.0016$ (black squares), and $x_w = 0.222$ (green triangles). Note the apparent linear dependence on temperature. **Note**: This figure has been updated in February, 2022; see published Correction to the paper.

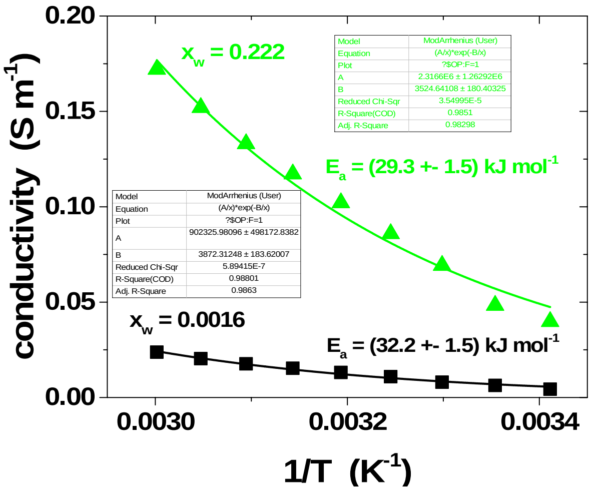

Figure S46. Fits according to the modified Arrhenius' law $\kappa = AT^{-1}\exp\left(-\Delta E_D / RT\right)$ to the conductivity data (conductivity corrected for thermal expansion). **Note**: This figure has been updated in February, 2022; see published Correction to the paper.

11. Local dynamics from NMR relaxation times

T_1 relaxation times

The major relaxation mechanism of the ^{13}C nuclei is dipole-dipole relaxations. According to the Bloembergen-Purcell-Pound (BPP) theory the relaxation rate is given by (assuming only dipole-dipole relaxation)[37,38]

$$\frac{1}{T_1^{DD}} = \frac{3n}{10} S^2 \left(\frac{\gamma_C \gamma_H \hbar}{r_{CH}^{\ 3}} \right)^2 \left[\frac{3\tau_c}{1+\omega_C^{\ 2}\tau_c^{\ 2}} + \frac{\tau_c}{1+\left(\omega_H - \omega_C\right)^2 \tau_c^{\ 2}} + \frac{6\tau_c}{1+\left(\omega_H + \omega_C\right)^2 \tau_c^{\ 2}} \right] = A_0 Z\left(\tau_c\right) \quad \text{(S15)}$$

Here γ_H and γ_C are the gyromagnetic ratios of the ^1H and ^{13}C nuclei, respectively, and r_{CH} is the distance between the relaxing carbon nucleus and the closest hydrogen nucleus (length of the C-H bond), n is 1,2, or 3 depending on the number of hydrogens bonded to the carbon nucleus, and $S^2 \le 1$ is an order parameter. All the T_1 relaxation times are short, << 20 s, and the effect of dissolved oxygen can be neglected.

The minimum ^{13}C T_1 relaxation time (maximum of the factor *Z*) is obtained when the correlation time is τ_c = 1.009 ns (when using a 500 MHz instrument)

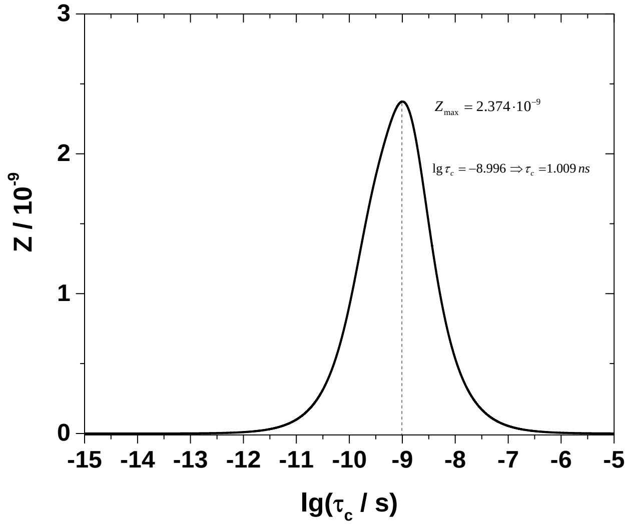

Figure S47. The factor $Z(\tau_c)$ in the eq. S15 for the ^{13}C T_1 relaxation time as a function of rotational correlation time τ_c according to the BPP theory (only dipole-dipole interaction).

The term A_0 in the equation is not the same for all nuclei but depends on the local environment. On the other hand, the factor *Z* determines the position of the minimum T_1 relaxation time on the correlation time axis. If the minimum is observed in the temperature dependence of T_1 that value

can be used to calculate A_0 for that particular nucleus, and the correlation times at all temperatures (at every value of T_1).

In this case, the T_1 relaxation times of the TBA C1' and C2' carbons in wet DES display minima, which allow calculate the factor A_0 for these nuclei. However, because of the strong distance dependence ($\propto r^{-6}$) A_0 can be assumed to be unaffected by the water content, and the same value can also be used for these nuclei in the dry DES sample.

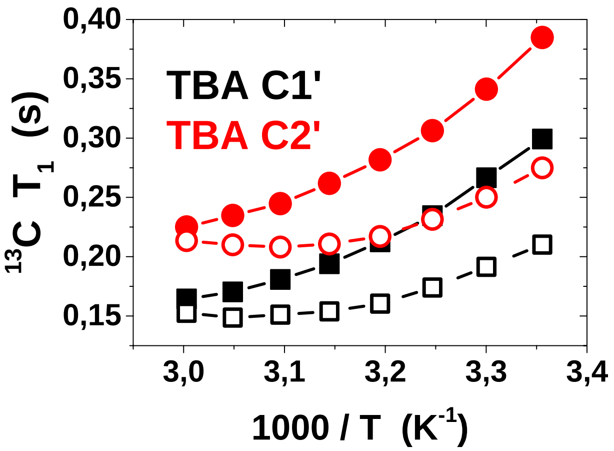

Figure S48. ^{13}C spin-lattice relaxation times for carbons C1' and C2' in TBA as a function of temperature. Solid lines and filled symbols for water mole fraction x_W = 0.0016 (dry DES), dashed lines and open symbols for x_W = 0.153 (wet DES). Lines are shown only as a guide to the eye.

For each nucleus, the factor A_0 is calculated from

$$A_0 = \left(Z_{\max} T_1^{(\min)}\right)^{-1} \qquad \text{(S16)}$$

The correlation time is then obtained from the equation[39]

$$G(\tau_c) = \left[\frac{3\tau_c}{1+\omega_C{}^2\tau_c{}^2} + \frac{\tau_c}{1+(\omega_H-\omega_C)^2\tau_c{}^2} + \frac{6\tau_c}{1+(\omega_H+\omega_C)^2\tau_c{}^2}\right] - \frac{1}{A_0 T_1^{DD}} = 0 \qquad \text{(S17)}$$

for each value of T_1 (corresponding to different temperatures). This equation has two roots, which cross at the T_1 minimum temperature. The two roots are illustrated below in the plots showing $G(\tau_c)$ at different temperatures for the carbons C1' and C2'.

The roots are plotted in the figure below as a function of temperature. Even though any observed T_1 relaxation time can, in principal, correspond to either of the roots, its temperature behavior sets the requirements for the physically meaningless roots in this temperature series; there is no reason for the correlation time to show nonmonotonous behavior as a function of temperature (at least in the absence of phase transitions). Therefore, even though the upper and lower roots cross at the temperature of the minimum T_1, the set of values showing an increase with decreasing temperature has to be chosen.[39]

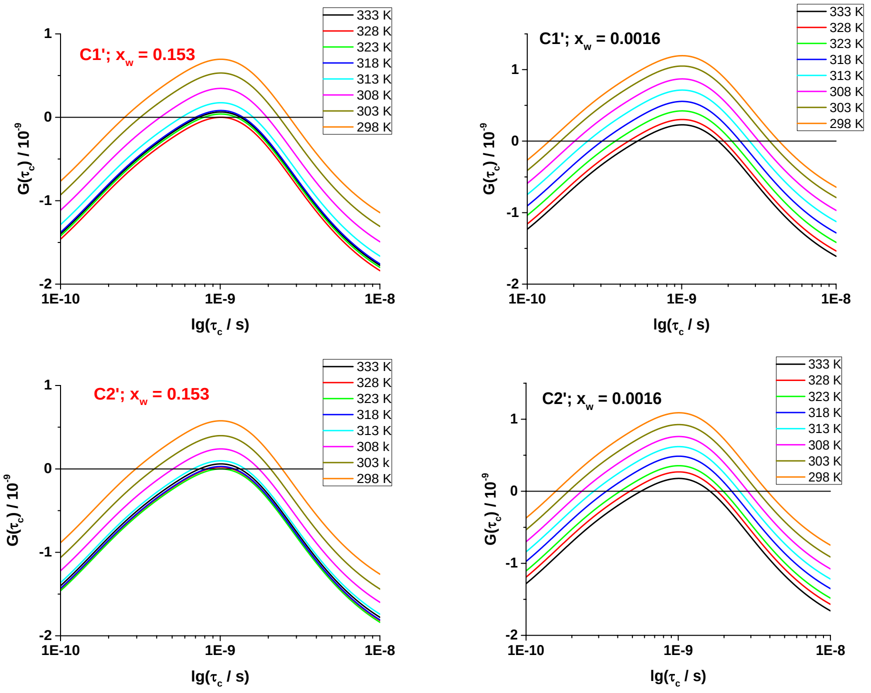

Figure S49. Function $G(\tau_c)$ for carbons C1' and C2' (in TBA) at different temperatures and water contents.

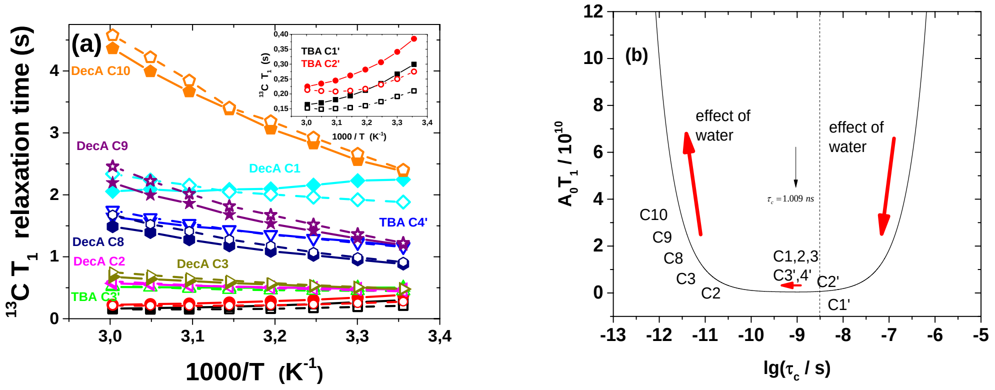

Figure S50. (a) ^{13}C spin-lattice relaxation times (T_1) in DES based on a 1:2 mixture of TBAC:DA as a function of temperature. **Solid lines** and **filled symbols** for water mole fraction x_W = 0.0016 (dry DES), ***dashed lines*** and ***open symbols*** for x_W = 0.153 (wet DES). Atom assignments shown in figures (primed atoms refer to TBA), lines are shown only as a guide to the eye.

(b) Schematic presentation (based on eq. S15) of the effect of water on the rotational correlation times based on the temperature behavior of the ^{13}C relaxation times. The dashed line at τ_c = 2 ns (approximate correlation time for the C1' and C2'). Location of atoms not accurate.

We have modelled the local dynamics by simulating the autocorrelation function of 4 bond vectors in TBA at different water contents. Integration of the autocorrelation function $\langle \mathbf{G}(t)\cdot\mathbf{G}(0)\rangle = \exp(-t/\tau)$ yields the average correlation time τ.

(a)

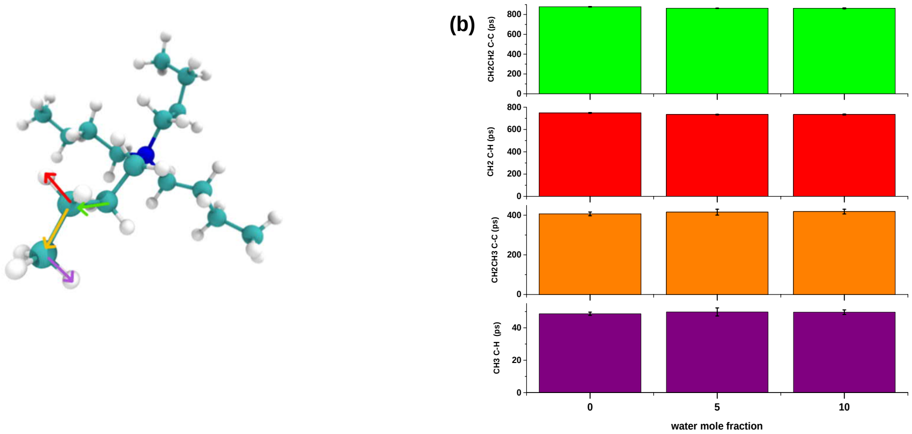

Figure S51. Simulation of C-C and C-H bond vector correlations; (a) the C2’-C3’, C3’-C4’, C3’-H, and C4’-H bond vectors; (b) average correlation times (in ps, same color coding). Standard deviation of simulation results shown.

According to the BPP theory the ^{1}H relaxation time is given by (only dipole-dipole relaxation)

$$\frac{1}{T_1^{DD}} = \left(\frac{3}{10}\gamma_H{}^4\hbar^2\sum_i \frac{1}{r_i^6}\right)\left(\frac{\tau_c}{1+\omega_H{}^2\tau_c{}^2}+\frac{4\tau_c}{1+4\omega_H{}^2\tau_c{}^2}\right) = A_0\left(\frac{\tau_c}{1+\omega_H{}^2\tau_c{}^2}+\frac{4\tau_c}{1+4\omega_H{}^2\tau_c{}^2}\right) = A_0 Z \tag{S18}$$

where γ_H is the gyromagnetic ratio of the ^{1}H nucleus, r_i’s are the H-H separations (because of the $\propto r^{-6}$ distance dependence only the closest neighbours have an effect), $\omega_H = 2\pi\nu_H$ ($\nu_H = 500$ MHz) and τ_c = rotational correlation time. The expression in the brackets (Z) has a maximum, when τ_c = 0.318 ns (500 MHz instrument), and T_1 has the maximum at this value of the rotational correlation time.

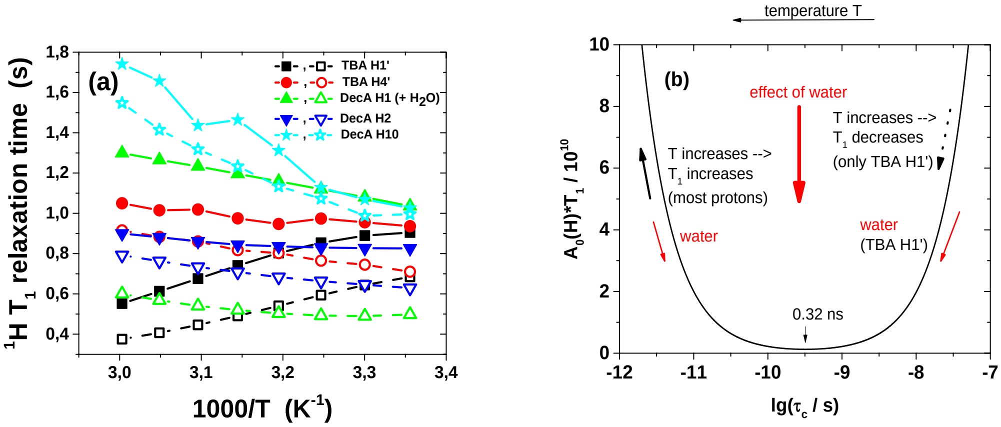

Figure S52. (a) ^{1}H spin-lattice relaxation times (T_1) in DES based on a 1:2 mixture of TBAC:DA as a function of temperature. ***Solid lines*** and ***filled symbols*** for water mole fraction x_W = 0.0016 (dry DES), ***dashed lines*** and ***open symbols*** for x_W = 0.153 (wet DES). Atom assignments shown in figures (primed atoms refer to TBA), lines are shown only as a guide to the eye. (b) Schematic presentation of the effect of water on the rotational correlation times based on the temperature dependence of the ^{1}H relaxation times. Exact positions of the different protons unknown.

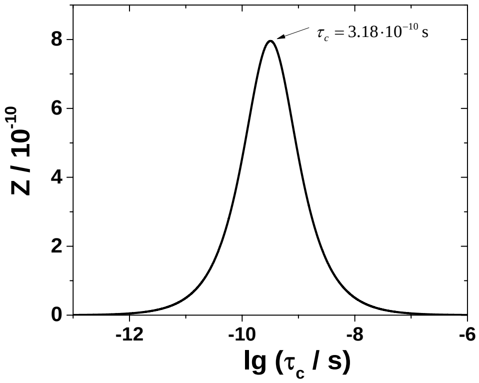

Figure S53. The factor *Z* (in parenthesis) in the equation (S18) for ^{1}H T_1 relaxation time as a function of rotational correlation time τ_c according to the BPP theory (only dipole-dipole interaction).

T_2* relaxation times

The T_2* relaxation times affect the NMR line widths and include contribution from both the spin-spin relaxation and magnetic field inhomogeneity (eq. S20), and they can be calculated from the full width at half height (FWHH) of the peak by

$$FWHH = \left(\pi T_2^{*}\right)^{-1} \qquad \text{(S19)}$$

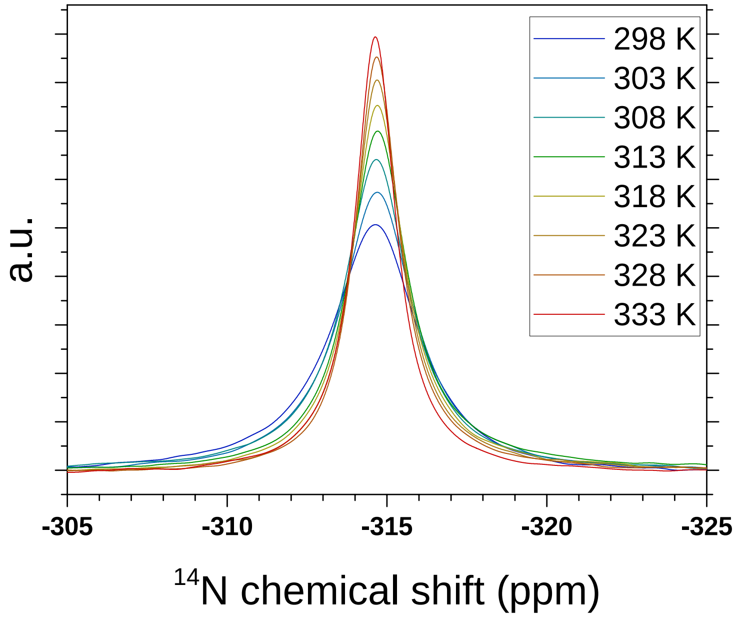

Figure S54. Effect of temperature on the ^{14}N peak of TBA in dry DES. The spectra are at 5 K intervals from 298 K (light pink) to 333 K (blue). The peak position remains constant within 0.1 ppm.

The peak width is caused by the natural line width and the inhomogeneity broadening, and the true T_2 relaxation time and the observed T_2* are related by

$$\frac{1}{T_2^*} = \frac{1}{T_2} + \gamma \Delta B_{\text{inhomog}} \qquad \text{(S20)}$$

The field inhomogeneity effects all nuclei but the line widths of the ^{13}C peaks are of the order of ~ 1 Hz, while the line width of the ^{14}N peaks varies between 13 – 81 Hz. Therefore, we can make the approximation in case of ^{14}N peaks

$$T_2 \approx T_2^*$$

The T_2 relaxation time is a decreasing function of the rotational correlation time τ_c,[37] and the higher ^{14}N T_2^* times in the wet DES suggest that water in DES enhances local dynamics by reducing the rotational correlation time of TBA.

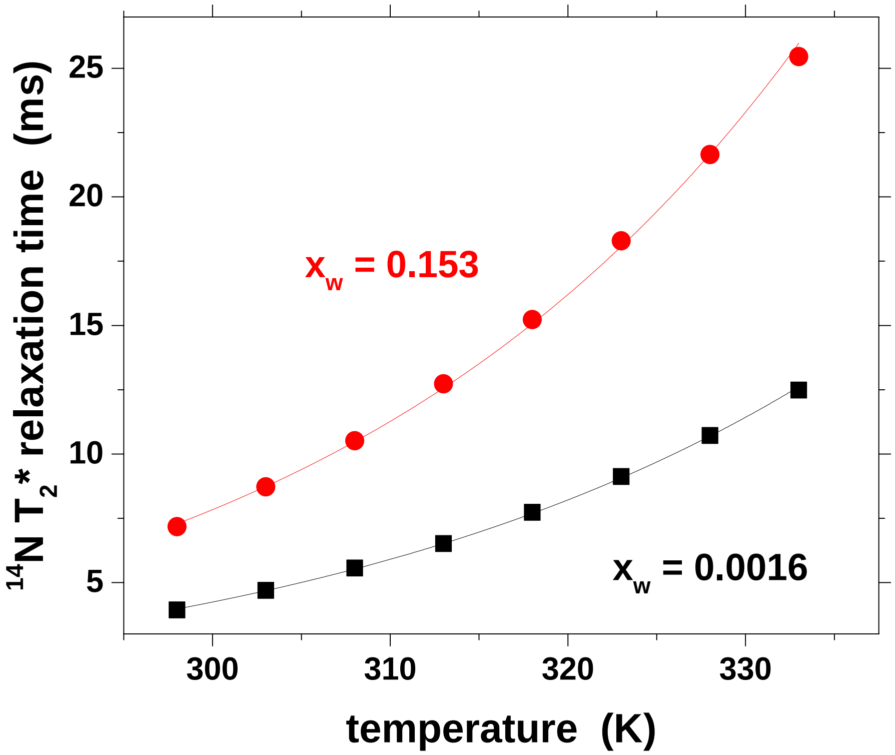

Figure S55. Effect of water content and temperature on the ^{14}N T_2^* relaxation time ($\approx T_2$) in DES. The lines are exponential fits to data.

12. Cluster analysis

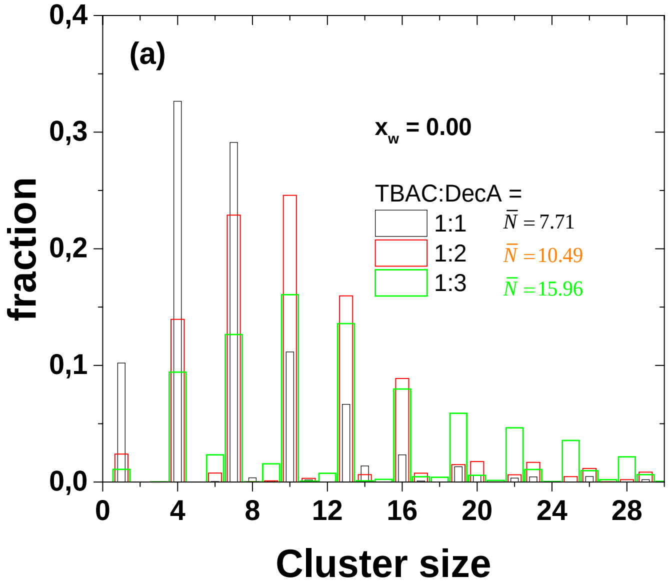

Figure S56. Distribution of the Cl···HOO cluster sizes in dry mixtures of TBAC and DecA at different mole fractions. The cluster sizes are given by $N = n_{Cl} + 3n_{COOH}$, where n_{Cl} and n_{COOH} are the number of chlorine ions and carboxylic groups associated within a cluster, respectively. Unequal bar widths for clarity. See below for typical cluster structures (clusters with *N* > 13 have many possible structures).

Table S7. Possible cluster structures (some cluster structures are speculative):

N	**structure**
**1**	Cl
**3**	(C)OOH
**4**	Cl-(C)OOH
**6**	$[(C)OOH]_2$
**7**	Cl –$[(C)OOH]_2$
**8**	$[Cl-(C)OOH]_2$ (minor)
**9**	Cl_3 –$[(C)OOH]_2$ (minor)
**10**	Cl-$[(C)OOH]_3$
**11**	Cl_2- $[(C)OOH]_3$ (minor)
**12**	Cl_3-$[(C)OOH]_3$ (minor)
**13**	Cl – $[(C)OOH]_4$
**16**	Cl – $[(C)OOH]_5$

Table S8. Effect of stoichiometry and water content on the average cluster size

**TBAC : DecA →**<br><br>**water mole fraction**<br>↓	**1 : 1**	**1 : 2**	**1 : 3**
**0.00**	7.71	10.49	15.96
**0.11**	7.54	10.47	15.08
**0.18**	7.67	10.63	14.33

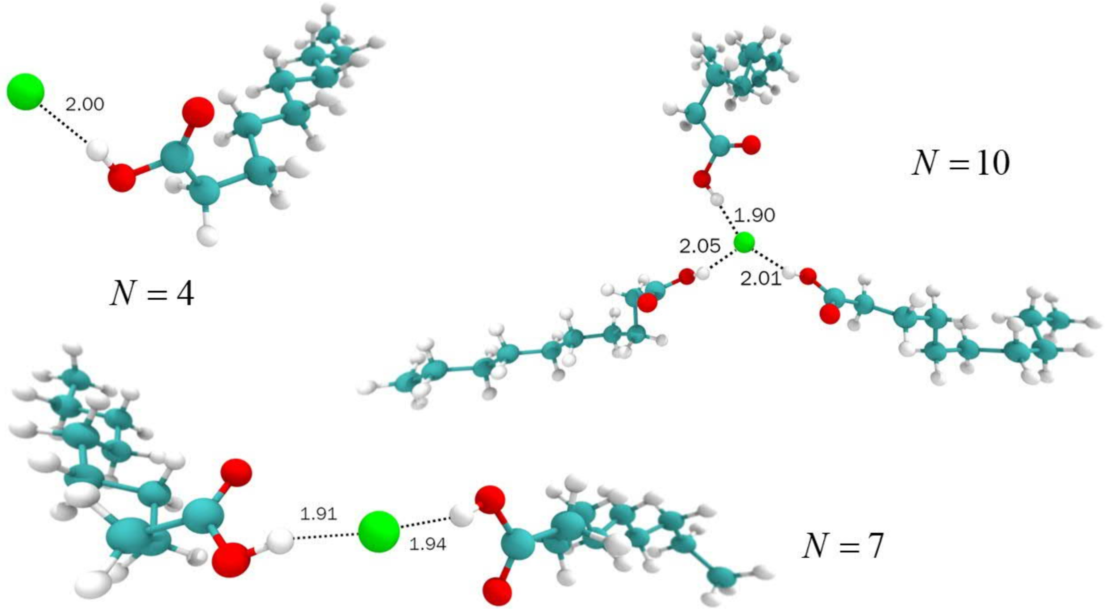

Figure S57. Snapshots of typical Cl ··· HOO- clusters of size N = 4, 7, and 10. Distances (in Å) from Cl to the carboxylic hydrogen shown. Color coding: Cl, green; C, blue; O, red; H, white.

13.References

(1) Biological Magnetic Resonance Data Bank (BMRB) https://bmrb.io/metabolomics/mol_summary/show_data.php?id=bmse000370 (accessed May 19, 2021). https://doi.org/10.13018/BMSE000370.

(2) Harris, R. K.; Becker, E. D.; Cabral De Menezes, S. M.; Granger, P.; Hoffman, R. E.; Zilm, K. W. Further Conventions for NMR Shielding and Chemical Shifts (IUPAC Recommendations 2008). *Pure and Applied Chemistry*. Walter de Gruyter GmbH 2008, pp 59–84. https://doi.org/10.1351/pac200880010059.

(3) Osch, D. J. G. P. van; Zubeir, L. F.; Bruinhorst, A. van den; Rocha, M. A. A.; Kroon, M. C. Hydrophobic Deep Eutectic Solvents as Water-Immiscible Extractants. *Green Chem.* **2015**, *17* (9), 4518–4521.

(4) Silverstein, R. M.; Webster, F. X.; Kiemle, D. J.; Bryce, D. L. *Specrometric Identification of Organic Compounds*, 8th ed.; Wiley, 2014.

(5) Abraham, M. J.; Murtola, T.; Schulz, R.; Páll, S.; Smith, J. C.; Hess, B.; Lindah, E. GROMACS: High Performance Molecular Simulations through Multi-Level Parallelism from Laptops to Supercomputers. *SoftwareX* **2015**, *1–2*, 19–25. https://doi.org/10.1016/J.SOFTX.2015.06.001.

(6) Páll, S.; Abraham, M. J.; Kutzner, C.; Hess, B.; Lindahl, E. Tackling Exascale Software Challenges in Molecular Dynamics Simulations with GROMACS. *Lect. Notes Comput. Sci. (including Subser. Lect. Notes Artif. Intell. Lect. Notes Bioinformatics)* **2014**, *8759*, 3–27. https://doi.org/10.1007/978-3-319-15976-8_1.

(7) Pronk, S.; Páll, S.; Schulz, R.; Larsson, P.; Bjelkmar, P.; Apostolov, R.; Shirts, M. R.; Smith, J. C.; Kasson, P. M.; van der Spoel, D.; et al. GROMACS 4.5: A High-Throughput

and Highly Parallel Open Source Molecular Simulation Toolkit. *Bioinformatics* **2013**, *29*, 845–854. https://doi.org/10.1093/bioinformatics/btt055.

(8) Hess*, B.; Kutzner, C.; Spoel, D. van der; Lindahl, E. GROMACS 4: Algorithms for Highly Efficient, Load-Balanced, and Scalable Molecular Simulation. **2008**. https://doi.org/10.1021/CT700301Q.

(9) Van Der Spoel, D.; Lindahl, E.; Hess, B.; Groenhof, G.; Mark, A. E.; Berendsen, H. J. C. GROMACS: Fast, Flexible, and Free. *J. Comput. Chem.* **2005**, *26* (16), 1701–1718. https://doi.org/10.1002/jcc.20291.

(10) Lindahl, E.; Hess, B.; van der Spoel, D. GROMACS 3.0: A Package for Molecular Simulation and Trajectory Analysis. *Mol. Model. Annu. 2001 78* **2001**, *7* (8), 306–317. https://doi.org/10.1007/S008940100045.

(11) Berendsen, H. J. C.; van der Spoel, D.; van Drunen, R. GROMACS: A Message-Passing Parallel Molecular Dynamics Implementation. *Comput. Phys. Commun.* **1995**, *91* (1–3), 43–56. https://doi.org/10.1016/0010-4655(95)00042-E.

(12) Bussi, G.; Donadio, D.; Parrinello, M. Canonical Sampling through Velocity Rescaling. *J. Chem. Phys.* **2007**, *126* (1), 014101. https://doi.org/10.1063/1.2408420.

(13) Berendsen, H. J. C.; Postma, J. P. M.; Van Gunsteren, W. F.; Dinola, A.; Haak, J. R. Molecular Dynamics with Coupling to an External Bath. *J. Chem. Phys.* **1984**, *81*, 3684–3690. https://doi.org/10.1063/1.448118.

(14) Parrinello, M.; Rahman, A. Polymorphic Transitions in Single Crystals: A New Molecular Dynamics Method. *J. Appl. Phys.* **1998**, *52* (12), 7182. https://doi.org/10.1063/1.328693.

(15) Hess, B. P-LINCS: A Parallel Linear Constraint Solver for Molecular Simulation. *J. Chem. Theory Comput.* **2007**, *4* (1), 116–122. https://doi.org/10.1021/CT700200B.

(16) Malde, A. K.; Zuo, L.; Breeze, M.; Stroet, M.; Poger, D.; Nair, P. C.; Oostenbrink, C.; Mark, A. E. An Automated Force Field Topology Builder (ATB) and Repository: Version 1.0. *J. Chem. Theory Comput.* **2011**, *7* (12), 4026–4037. https://doi.org/10.1021/CT200196M/SUPPL_FILE/CT200196M_SI_001.PDF.

(17) Jorgensen, W. L.; Tirado-Rives, J. Potential Energy Functions for Atomic-Level Simulations of Water and Organic and Biomolecular Systems. *Proc. Natl. Acad. Sci.* **2005**, *102* (19), 6665–6670. https://doi.org/10.1073/PNAS.0408037102.

(18) Dodda, L. S.; Vilseck, J. Z.; Tirado-Rives, J.; Jorgensen, W. L. 1.14∗CM1A-LBCC: Localized Bond-Charge Corrected CM1A Charges for Condensed-Phase Simulations. *J. Phys. Chem. B* **2017**, *121* (15), 3864–3870. https://doi.org/10.1021/ACS.JPCB.7B00272/SUPPL_FILE/JP7B00272_SI_002.XLS.

(19) Dodda, L. S.; De Vaca, I. C.; Tirado-Rives, J.; Jorgensen, W. L. LigParGen Web Server: An Automatic OPLS-AA Parameter Generator for Organic Ligands. *Nucleic Acids Res.* **2017**, *45* (W1), W331–W336. https://doi.org/10.1093/NAR/GKX312.

(20) M. J. Frisch, G. W. Trucks, H. B. Schlegel, G. E. Scuseria, M. A. Robb, J. R. Cheeseman, G. Scalmani, V. Barone, G. A. Petersson, H. Nakatsuji, X. Li, M. Caricato, A. Marenich, J. Bloino, B. G. Janesko, R. Gomperts, B. Mennucci, H. P. Hratchian, J. V. Ort, and D. J. F. Gaussian09. Gaussian, Inc., Wallingford CT, 2016 2016.

(21) Mainberger, S.; Kindlein, M.; Bezold, F.; Elts, E.; Minceva, M.; Briesen, H. Deep Eutectic Solvent Formation: A Structural View Using Molecular Dynamics Simulations with Classical Force Fields. *Mol. Phys.* **2017**, *115* (9–12), 1309–1321. https://doi.org/10.1080/00268976.2017.1288936.

(22) Schmid, N.; Eichenberger, A. P.; Choutko, A.; Riniker, S.; Winger, M.; Mark, A. E.; Van

Gunsteren, W. F. Definition and Testing of the GROMOS Force-Field Versions 54A7 and 54B7. *Eur. Biophys. J.* **2011**, *40* (7), 843–856. https://doi.org/10.1007/s00249-011-0700-9.

(23) and, I.-C. Y.; Hummer*, G. System-Size Dependence of Diffusion Coefficients and Viscosities from Molecular Dynamics Simulations with Periodic Boundary Conditions. **2004**. https://doi.org/10.1021/JP0477147.

(24) Head-Gordon, T.; Hura, G. Water Structure from Scattering Experiments and Simulation. *Chem. Rev.* **2002**, *102*, 2651–2670. https://doi.org/10.1021/CR0006831.

(25) Hammond, O. S.; Bowron, D. T.; Jackson, A. J.; Arnold, T.; Sanchez-Fernandez, A.; Tsapatsaris, N.; Garcia Sakai, V.; Edler, K. J. Resilience of Malic Acid Natural Deep Eutectic Solvent Nanostructure to Solidification and Hydration. *J. Phys. Chem. B* **2017**, *121* (31), 7473–7483. https://doi.org/10.1021/acs.jpcb.7b05454.

(26) Botti, A.; Bruni, F.; Imberti, S.; Ricci, M. A.; Soper, A. K. Ions in Water: The Microscopic Structure of a Concentrated HCl Solution. *J. Chem. Phys.* **2004**, *121* (16), 7840. https://doi.org/10.1063/1.1801031.

(27) Bruni, F.; Imberti, S.; Mancinelli, R.; Ricci, M. A. Aqueous Solutions of Divalent Chlorides: Ions Hydration Shell and Water Structure. *J. Chem. Phys.* **2012**, *136* (6), 064520. https://doi.org/10.1063/1.3684633.

(28) Mancinelli, R.; Botti, A.; Bruni, F.; Ricci, M. A.; Soper, A. K. Hydration of Sodium, Potassium, and Chloride Ions in Solution and the Concept of Structure Maker/Breaker. *J. Phys. Chem. B* **2007**, *111* (48), 13570–13577. https://doi.org/10.1021/jp075913v.

(29) Bhowmik, D.; Malikova, N.; Mériguet, G.; Bernard, O.; Teixeira, J.; Turq, P. Aqueous Solutions of Tetraalkylammonium Halides: Ion Hydration, Dynamics and Ion–Ion Interactions in Light of Steric Effects. *Phys. Chem. Chem. Phys.* **2014**, *16* (26), 13447–

13457. https://doi.org/10.1039/C4CP01164C.

(30) Feng, G.; Huang, J.; Sumpter, B. G.; Meunier, V.; Qiao, R. Structure and Dynamics of Electrical Double Layers in Organic Electrolytes. *Phys. Chem. Chem. Phys.* **2010**, *12* (20), 5468. https://doi.org/10.1039/c000451k.

(31) Zhang, M.; Chen, L.; Yang, H.; Ma, J. Theoretical Study of Acetic Acid Association Based on Hydrogen Bonding Mechanism. *J. Phys. Chem. A* **2017**, *121* (23), 4560–4568. https://doi.org/10.1021/acs.jpca.7b03324.

(32) Jianhan Chen, ‡; Charles L. Brooks, III,*, A.; Harold A. Scheraga*, §. Revisiting the Carboxylic Acid Dimers in Aqueous Solution: Interplay of Hydrogen Bonding, Hydrophobic Interactions, and Entropy†. *J. Phys. Chem.* **2008**, *112*, 242–249. https://doi.org/10.1021/JP074355H.

(33) Abbott, A. P.; Harris, R. C.; Ryder, K. S. Application of Hole Theory to Define Ionic Liquids by Their Transport Properties. *J. Phys. Chem. B* **2007**, *111* (18), 4910–4913.

(34) Böhmer, R.; Angell, C. A. Correlations of the Nonexponentiality and State Dependence of Mechanical Relaxations with Bond Connectivity in Ge-As-Se Supercooled Liquids. *Phys. Rev. B* **1992**, *45* (17), 10091–10094. https://doi.org/10.1103/PhysRevB.45.10091.

(35) Böhmer, R.; Ngai, K. L.; Angell, C. A.; Plazek, D. J. Nonexponential Relaxations in Strong and Fragile Glass Formers. *J. Chem. Phys.* **1993**, *99* (5), 4201–4209. https://doi.org/10.1063/1.466117.

(36) Angell, C. A. Perspective on the Glass Transition. *J. Phys. Chem. Solids* **1988**. https://doi.org/10.1016/0022-3697(88)90002-9.

(37) Levitt, M. H. *Spin Dynamics: Basics of Nuclear Magnetic Resonance, Second Edition*, 2nd ed.; Wiley: New York, 2009; Vol. 37. https://doi.org/10.1118/1.3273534.

(38) Dugoni, G. C.; Di Pietro, M. E.; Ferro, M.; Castiglione, F.; Ruellan, S.; Moufawad, T.; Moura, L.; Costa Gomes, M. F.; Fourmentin, S.; Mele, A. Effect of Water on Deep Eutectic Solvent/β-Cyclodextrin Systems. *ACS Sustain. Chem. Eng.* **2019**, *7* (7), 7277–7285. https://doi.org/10.1021/acssuschemeng.9b00315.

(39) Matveev, V. V.; Markelov, D. A.; Brui, E. A.; Chizhik, V. I.; Ingman, P.; Lähderanta, E. 13C NMR Relaxation and Reorientation Dynamics in Imidazolium-Based Ionic Liquids: Revising Interpretation. *Phys. Chem. Chem. Phys.* **2014**, *16* (22), 10480–10484. https://doi.org/10.1039/c4cp00637b.

Appendix: GROMACS compatible molecular topologies and starting structures

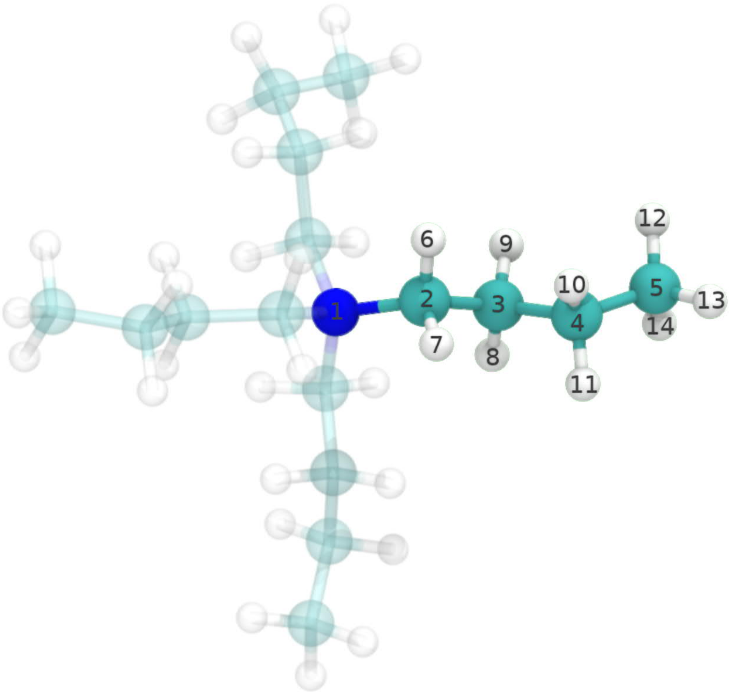

Figure SIApp1: The all-atom structure of tetrabutylammonium-cation. Bonded and non-bonded terms of each butyl-chain are identical, and parameters for the transparent parts are omitted.

Table SIApp1: General descriptors of the tetrabutylammonium-cation.

**General information for tetrabutylammonium-cation**			
**Number**	**Atomtype**	**Charge**	**Mass**
1	NL	0.032	14.0067
2	C	-0.126	12.0010
3	C	-0.182	12.0010
4	C	-0.158	12.0010
5	C	-0.217	12.0010
6	HC	0.135	1.0080
7	HC	0.135	1.0080
8	HC	0.103	1.0080

9	HC	0.103	1.0080
10	HC	0.091	1.0080
11	HC	0.091	1.0080
12	HC	0.089	1.0080
13	HC	0.089	1.0080
14	HC	0.089	1.0080

Table SIApp2: Bond terms for tetrabutylammonium-cation. Atom numbers refer to those presented in Figure S[X] Bond type refers to the GROMACS bond types, which can be found in the reference manual, table 5.5. (table 5.14. in newer versions). Backbone bonds are highlighted in gray, and heavy atoms are indicated by a bold font.

**Bond terms for tetrabutylammonium-cation**				
Atom 1	Atom 2	Bond type	Bond length (nm)	Force constant ($kJ \times mol^{-1} \times nm^{-4}$)
**1**	**2**	G96 bond	0.1510	3.7279×10^{6}
**2**	**3**	G96 bond	0.1520	5.4300×10^{6}
**3**	**4**	G96 bond	0.1520	5.4300×10^{6}
**4**	**5**	G96 bond	0.1510	3.7279×10^{6}
**2**	6	G96 bond	0.1130	7.0483×10^{6}
**2**	7	G96 bond	0.1130	7.0483×10^{6}
**3**	8	G96 bond	0.1120	3.7000×10^{7}
**3**	9	G96 bond	0.1120	3.7000×10^{7}
**4**	10	G96 bond	0.1120	3.7000×10^{7}
**4**	11	G96 bond	0.1120	3.7000×10^{7}
**5**	12	G96 bond	0.1120	3.7000×10^{7}
**5**	13	G96 bond	0.1120	3.7000×10^{7}
**5**	14	G96 bond	0.1120	3.7000×10^{7}

Table SIApp3: Angle terms for tetrabutylammonium-cation. Backbone angles are highlighted in gray, and heavy atoms are indicated by a bold font.

Angle terms for tetrabutylammonium-cation					
Atom 1	Atom 2	Atom 3	Angle type	Angle size (deg.)	Force constant (kJ×mol^{-1})
**3**	**2**	**1**	G96 angle	115.00	610.00
**4**	**3**	**2**	G96 angle	109.50	520.00
**5**	**4**	**3**	G96 angle	111.00	530.00
6	**2**	**1**	G96 angle	107.00	2726.16
7	**2**	**1**	G96 angle	107.00	2726.16
7	**2**	6	G96 angle	108.00	465.00
**3**	**2**	6	G96 angle	109.00	1680.51
**3**	**2**	7	G96 angle	109.00	1680.51
8	**3**	**2**	G96 angle	111.00	530.00
9	**3**	**2**	G96 angle	111.00	530.00
9	**3**	8	G96 angle	108.00	465.00
**4**	**3**	8	G96 angle	109.00	1680.51
**4**	**3**	9	G96 angle	109.00	1680.51
10	**4**	**3**	G96 angle	109.60	450.00
11	**4**	**3**	G96 angle	109.60	450.00
**5**	**4**	**3**	G96 angle	111.00	530.00
**5**	**4**	10	G96 angle	109.60	450.00
**5**	**4**	11	G96 angle	109.60	450.00
11	**4**	10	G96 angle	107.57	484.00
12	**5**	**4**	G96 angle	110.00	4763.46
14	**5**	**4**	G96 angle	110.00	4763.46
12	**5**	13	G96 angle	108.53	443.00

12	**5**	14	G96 angle	108.53	443.00
13	**5**	14	G96 angle	108.53	443.00

Table SIApp4: Dihedral terms for tetrabutylammonium-cation. Backbone dihedrals are highlighted in gray, and heavy atoms are indicated by a bold font.

**Dihedral terms for tetrabutylammonium-cation**							
Atom 1	Atom 2	Atom 3	Atom 4	Dihedral type	Angle size (deg)	Force constant (kJ $\times$mol^{-1})	Multiplicity
**4**	**3**	**2**	**1**	Proper	0.00	5.92	3
**5**	**4**	**3**	**2**	Proper	0.00	5.92	3
13	**5**	**4**	**3**	Proper	0.00	5.92	3

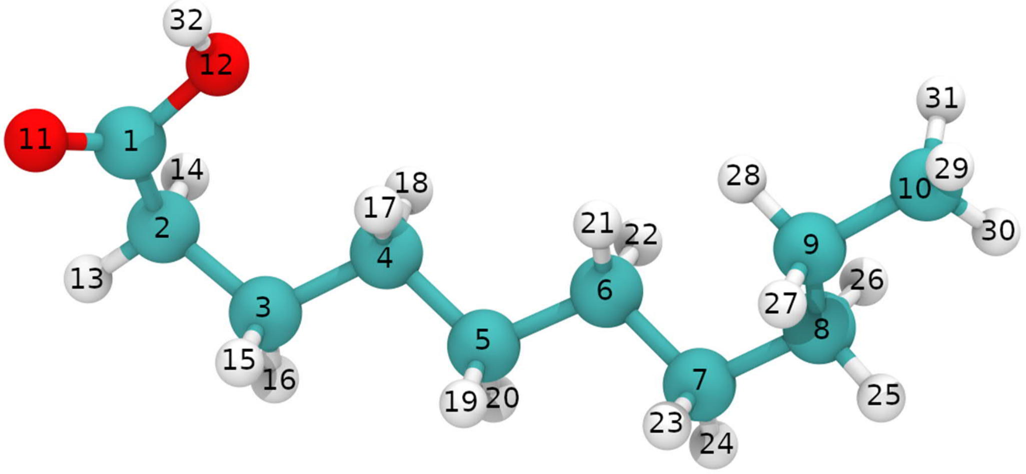

Figure SIApp2: The all-atom structure of decanoic acid.

Table SIApp5: General descriptors of decanoic acid.

General information for decanoic acid			
**Number**	**Atomtype**	**Charge**	**Mass**
1	C	0.740	14.0067
2	C	-0.121	12.0010
3	C	-0.009	12.0010
4	C	-0.007	12.0010
5	C	-0.038	12.0010
6	C	0.120	12.0010
7	C	-0.095	12.0010
8	C	-0.001	12.0010
9	C	0.140	12.0010

10	C	-0.301	12.0010
11	O	-0.585	15.9994
12	OA	-0.623	15.9994
13	HC	0.067	1.0080
14	HC	0.067	1.0080
15	HC	0.012	1.0080
16	HC	0.012	1.0080
17	HC	0.007	1.0080
18	HC	0.007	1.0080
19	HC	0.008	1.0080
20	HC	0.008	1.0080
21	HC	-0.027	1.0080
22	HC	-0.027	1.0080
23	HC	0.012	1.0080
24	HC	0.012	1.0080
25	HC	0.003	1.0080
26	HC	0.003	1.0080
27	HC	-0.023	1.0080

28	HC	-0.023	1.0080
29	HC	0.069	1.0080
30	HC	0.069	1.0080
31	HC	0.069	1.0080
32	HS14	0.455	1.0080

Table SIApp6: Bond terms for decanoic acid. Atom numbers refer to those presented in Fig. SIApp2. Bond type refers to the GROMACS bond types, which can be found in the reference manual, table 5.5. (table 5.14. in newer versions). Backbone bonds are highlighted in gray, and heavy atoms are indicated by a bold font.

Bond terms for decanoic acid				
Atom 1	Atom 2	Bond type	Bond length (nm)	Force constant (kJ×mol^{-1}×nm^{-4})
**1**	**2**	G96 bond	0.1520	5.4300×10^{6}
**2**	**3**	G96 bond	0.1530	7.1500×10^{6}
**3**	**4**	G96 bond	0.1530	7.1500×10^{6}
**4**	**5**	G96 bond	0.1530	7.1500×10^{6}
**5**	**6**	G96 bond	0.1530	7.1500×10^{6}
**6**	**7**	G96 bond	0.1530	7.1500×10^{6}
**7**	**8**	G96 bond	0.1530	7.1500×10^{6}
**8**	**9**	G96 bond	0.1530	7.1500×10^{6}
**9**	**10**	G96 bond	0.1530	7.1500×10^{6}
**1**	**11**	G96 bond	0.1220	2.2843×10^{7}
**1**	**12**	G96 bond	0.1360	1.0200×10^{7}
**2**	13	G96 bond	0.1090	1.2300×10^{7}
**2**	14	G96 bond	0.1090	1.2300×10^{7}

**3**	15	G96 bond	0.1090	1.2300×10^7
**3**	16	G96 bond	0.1090	1.2300×10^7
**4**	17	G96 bond	0.1100	1.2100×10^7
**4**	18	G96 bond	0.1100	1.2100×10^7
**5**	19	G96 bond	0.1100	1.2100×10^7
**5**	20	G96 bond	0.1100	1.2100×10^7
**6**	21	G96 bond	0.1100	1.2100×10^7
**6**	22	G96 bond	0.1100	1.2100×10^7
**7**	23	G96 bond	0.1100	1.2100×10^7
**7**	24	G96 bond	0.1100	1.2100×10^7
**8**	25	G96 bond	0.1100	1.2100×10^7
**8**	26	G96 bond	0.1100	1.2100×10^7
**9**	27	G96 bond	0.1090	1.2300×10^7
**9**	28	G96 bond	0.1090	1.2300×10^7
**10**	29	G96 bond	0.1090	1.2300×10^7
**10**	30	G96 bond	0.1090	1.2300×10^7
**10**	31	G96 bond	0.1090	1.2300×10^7
**12**	32	G96 bond	0.0972	1.9581×10^7

Table SIApp7: Angle terms for decanoic acid. Backbone angles are highlighted in gray, and heavy atoms are indicated by a bold font.

**Angle terms for decanoic acid**					
Atom 1	Atom 2	Atom 3	Angle type	Angle size (deg.)	Force constant ($kJ \times mol^{-1}$)
**1**	**2**	**3**	G96 angle	111.00	530.00
**2**	**3**	**4**	G96 angle	111.00	530.00
**3**	**4**	**5**	G96 angle	111.00	530.00
**4**	**5**	**6**	G96 angle	111.00	530.00

**5**	**6**	**7**	G96 angle	111.00	530.00
**6**	**7**	**8**	G96 angle	111.00	530.00
**7**	**8**	**9**	G96 angle	111.00	530.00
**8**	**9**	**10**	G96 angle	111.00	530.00
**12**	**1**	**11**	G96 angle	124.00	730.00
**12**	**1**	**2**	G96 angle	111.00	530.00
**11**	**1**	**2**	G96 angle	126.00	640.00
**1**	**2**	13	G96 angle	107.60	507.00
**1**	**2**	14	G96 angle	107.60	507.00
13	**2**	14	G96 angle	106.75	503.00
13	**2**	**3**	G96 angle	111.00	530.00
14	**2**	**3**	G96 angle	111.00	530.00
**2**	**3**	15	G96 angle	109.50	450.00
**2**	**3**	16	G96 angle	109.50	450.00
15	**3**	16	G96 angle	106.75	503.00
15	**3**	**4**	G96 angle	109.60	450.00
16	**3**	**4**	G96 angle	109.60	450.00
**3**	**4**	17	G96 angle	109.50	450.00
**3**	**4**	18	G96 angle	109.50	450.00
17	**4**	18	G96 angle	106.75	503.00
17	**4**	**5**	G96 angle	109.50	450.00
18	**4**	**5**	G96 angle	109.50	450.00
**4**	**5**	19	G96 angle	109.50	450.00
**4**	**5**	20	G96 angle	109.50	450.00
19	**5**	20	G96 angle	106.75	503.00
19	**5**	**6**	G96 angle	109.50	450.00

20	**5**	**6**	G96 angle	109.50	450.00
**5**	**6**	21	G96 angle	109.50	450.00
**5**	**6**	22	G96 angle	109.50	450.00
21	**6**	22	G96 angle	106.75	503.00
21	**6**	**7**	G96 angle	109.50	450.00
22	**6**	**7**	G96 angle	109.50	450.00
**6**	**7**	23	G96 angle	109.50	450.00
**6**	**7**	24	G96 angle	109.50	450.00
23	**7**	24	G96 angle	106.75	503.00
23	**7**	**8**	G96 angle	109.50	450.00
24	**7**	**8**	G96 angle	109.50	450.00
**7**	**8**	25	G96 angle	109.50	450.00
**7**	**8**	26	G96 angle	109.50	450.00
25	**8**	26	G96 angle	106.75	503.00
25	**8**	**9**	G96 angle	109.50	450.00
26	**8**	**9**	G96 angle	109.50	450.00
**8**	**9**	27	G96 angle	109.50	450.00
**8**	**9**	28	G96 angle	109.50	450.00
27	**9**	28	G96 angle	106.75	503.00
27	**9**	**10**	G96 angle	109.50	450.00
28	**9**	**10**	G96 angle	109.50	450.00
**9**	**10**	30	G96 angle	111.40	532.00
**9**	**10**	31	G96 angle	111.40	532.00
**9**	**10**	32	G96 angle	111.40	532.00
29	**10**	30	G96 angle	107.57	484.00
29	**10**	31	G96 angle	107.57	484.00

30	**10**	31	G96 angle	107.57	484.00
32	**12**	**1**	G96 angle	109.50	450.00

Table SIApp8: Dihedral terms for decanoic acid. Backbone dihedrals are highlighted in gray, and heavy atoms are indicated by a bold font.

**Dihedral terms for decanoic acid**							
Atom 1	Atom 2	Atom 3	Atom 4	Dihedral type	Angle size (deg)	Force constant (kJ $\times$mol^{-1})	Multiplicity
**1**	**2**	**3**	**4**	Proper	0.00	5.92	3
**2**	**3**	**4**	**5**	Proper	0.00	5.92	3
**3**	**4**	**5**	**6**	Proper	0.00	5.92	3
**4**	**5**	**6**	**7**	Proper	0.00	5.92	3
**5**	**6**	**7**	**8**	Proper	0.00	5.92	3
**6**	**7**	**8**	**9**	Proper	0.00	5.92	3
**7**	**8**	**9**	**10**	Proper	0.00	5.92	3
**11**	**1**	**2**	**3**	Proper	180.00	1.00	6
**8**	**9**	**10**	29	Proper	0.00	5.92	3
32	**12**	**1**	**2**	Proper	180.00	16.70	2
